# Learning Curves in Prospective Life Cycle Assessment

**DOI:** 10.1021/acs.est.5c03870

**Published:** 2025-07-30

**Authors:** Mitchell K. van der Hulst, Mara Hauck, Selwyn Hoeks, Rosalie van Zelm, Mark A. J. Huijbregts

**Affiliations:** † Department of Environmental Science, Radboud Institute for Biological and Environmental Sciences, P.O. Box 9010, Nijmegen 6500 GL, The Netherlands; ‡ Circularity & Sustainability Impact Department, TNO, P.O. Box 80015, Utrecht 3508 TA, The Netherlands; § Technology, Innovation & Society, Department of Industrial Engineering & Innovation Sciences, Eindhoven University of Technology, P.O. Box 513, Eindhoven 5600 MB, The Netherlands

**Keywords:** LCA, environmental footprint, ex ante, technological change, emerging technology, technological learning, photovoltaics, experience curve

## Abstract

Environmental learning curves have great potential to predict future changes in environmental footprints of technologies as part of prospective life cycle assessments. However, concrete guidance is currently missing on how to integrate environmental learning curves into prospective life cycle assessments. Here, we propose a method to combine (i) process-specific environmental learning curves for key technology parameters and (ii) projections from integrated assessment models to include relevant changes in background processes, such as expected decarbonization of the electricity grid. Our method enables process contribution analyses, uncertainty and sensitivity analyses, and flexibility in the assessment of various impact categories. Application of our proposed method is demonstrated in a case study assessing various environmental footprints of producing monocrystalline silicon photovoltaic panels. We showed that environmental footprints reduce 21–80% between 2020 and 2050 through a synergy of (i) and (ii). Footprint reductions were mostly driven by background changes when decarbonization is extensive, whereas process-specific environmental learning curves become the major driver for footprint reductions when developments in background processes follow a similar trajectory as charted by the past. Our method may also be used in the assessment of emerging technologies by applying process-specific environmental learning curves to mature parts of their supply chain.

## Graphical abstract

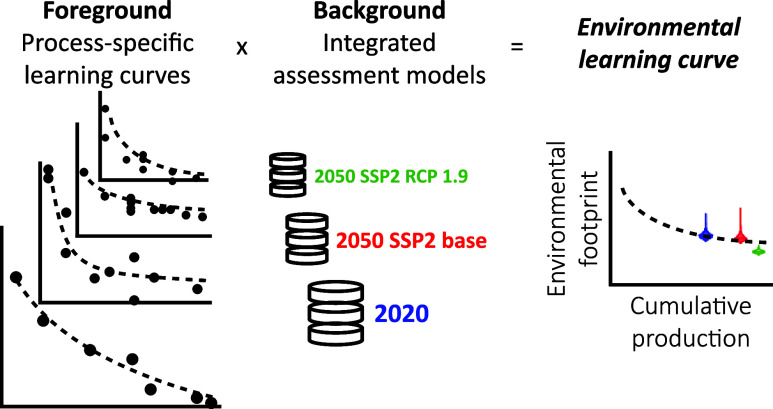

## 1 Introduction

Human activities are increasingly under scrutiny for their detrimental effects on the natural environment. Life cycle assessment (LCA) is a method that aims to quantify the impacts of human activity, such as extraction of resources from nature and emissions of pollutants and wastes to nature.^1^ LCA is traditionally applied to assess the current environmental performance of human activities. However, recent years have seen a growth in the number of prospective LCA studies assessing the future environmental performance of human activities.^2^ These prospective studies distinguish themselves from ordinary LCA in that they explicitly try to quantify environmental performance at a future point in time, typically with the aim to inform and guide technology developers and policy makers toward high environmental performance.

Such future-oriented assessments require extrapolation from the current state to what is a likely future state. Literature studies such as Buyle et al.^3^ have identified diverse procedures that LCA practitioners can use to perform these extrapolations in a systematic and scientific way. One of these is learning curves, which are statistical representations of learning processes observed in human activities that follow a power law known as Wright’s law.^4^ In simple terms, the more a task is repeated, the better the outcome of the task as a result of learning. Improvements are initially rapid but gradually slow down as knowledge and efficiency reach a maximum. Thus, improvements as a function of learning follow a curved path, i.e., the learning curve. In economics, learning curves have long been used to project decreases in production cost due to learning induced increases in efficiency. However, environmental impacts were also observed to reduce due to increased efficiency.^5^ Learning curves related to environmental parameters have therefore raised the interest of the prospective LCA community, as they can easily be extrapolated to forecast the future environmental performance of a human activity.

Louwen et al.,^6^ for example, used LCA studies from 1989 to 2013 to derive product-specific environmental learning curves for the greenhouse gas (GHG) footprint of solar panel production as a function of cumulative installed photovoltaic (PV) capacity and extrapolated GHG footprints for 2040. Similarly, van Nielen et al.^7^ used historical data for the GHG footprint of copper production. While both studies demonstrate that the environmental performance of products follows trends that can be extrapolated, the applicability of their approach in prospective LCA is limited. For one, it is limited to impact categories for which results are reported or for which learning rates can be estimated by referring to another impact category, cost, or performance indicator. Caduff et al.^8^ presented a solution by combining life cycle inventory (LCI) data from various LCA studies to construct environmental learning curves, thereby enabling the assessment of a wide array of impact categories. However, this is only possible for products that have been extensively studied over a wide timespan, while being intrinsically impossible for new products that are often studied in prospective LCA.^9^ Bergesen and Suh^10^ presented a solution with their framework for technological learning in the supply chain. Instead of using multiple LCA studies, they collected time-series data for consumption of key material and energy sources used in the supply chain. Process-specific learning curves for individual processes are then created, which are applied to the LCI. This approach is especially useful in prospective LCA because one could use time-series data for any relevant process parameter that exhibits learning behavior. For example, increased efficiency of electric motors can be converted to process-specific learning curves for the kilowatt-hours of electricity consumed in processes using electric motors. Time-series data for process parameters are found in a wider variety of data sources than solely LCA studies. A further benefit is that process-specific learning curves enable process contribution analyses and uncertainty and sensitivity analyses. Application of the framework is demonstrated by Bergesen and Suh^10^ and Koj et al.^11^ Both applied learning curves to account for endogenous changes in the foreground system. However, exogenous changes in the background system were excluded by Koj et al.,^11^ while Bergesen and Suh^10^ included learning in only a hand full of background process, applying a single estimated learing rate (LR) to all parameters. A more comprehensive inclusion of exogenous changes is demonstrated by Fozer et al.,^12^ but they base endogenous changes on explorative normative scenarios instead of learning curves.

In our previous work,^13^ we proposed to couple endogenous changes in the foreground system using learning curves with exogenous changes in the background system using projections from integrated assessment models (IAMs) when conducting a prospective LCA of a mature, industrially produced technology. An IAM is a model applied to analyze the interactions between socio-economic developments and the environment in order to provide insights about the impacts of different decisions and policies. The use of IAMs to comprehensively model changes in the background system of LCAs was comprehensibly demonstrated by Mendoza Beltran et al.^14^ and has since been made more widely available by Sacchi et al.^15^ through their *premise* framework. While inclusion of background changes using IAMs is starting to become commonplace in prospective LCA, its coupling with foreground changes through the application of learning curves and the added value of such coupling are yet to be demonstrated.

Here, our goal is to develop and apply a method for the combined use of (i) process-specific environmental learning curves for key parameters of the technology that is to be evaluated and (ii) projections from IAMs to model changes in background processes, such as expected decarbonization of the electricity grid. The application of our method is demonstrated with a case study for the production of monocrystalline silicon solar panels. We selected this mature technology, instead of an emerging technology, to showcase the use of diverse data sources and strategies, as well as to enable comparison with an existing environmental learning curve based on empirical data as reported by Louwen et al.^6^ Considering the uncertain nature of prospective LCA, we quantified uncertainty introduced by the application of learning curves. Based on lessons learned from the case study, a discussion of challenges and limitations and an outlook are provided for using our method in prospective LCA.

## 2 Methods

### 2.1 Integration of Learning in Foreground and Background Processes within Prospective LCA

Our proposed method consists of four consecutive phases depicted as a flowchart in Figure 1 and explained in detail in Table 1.

**Figure 1:**
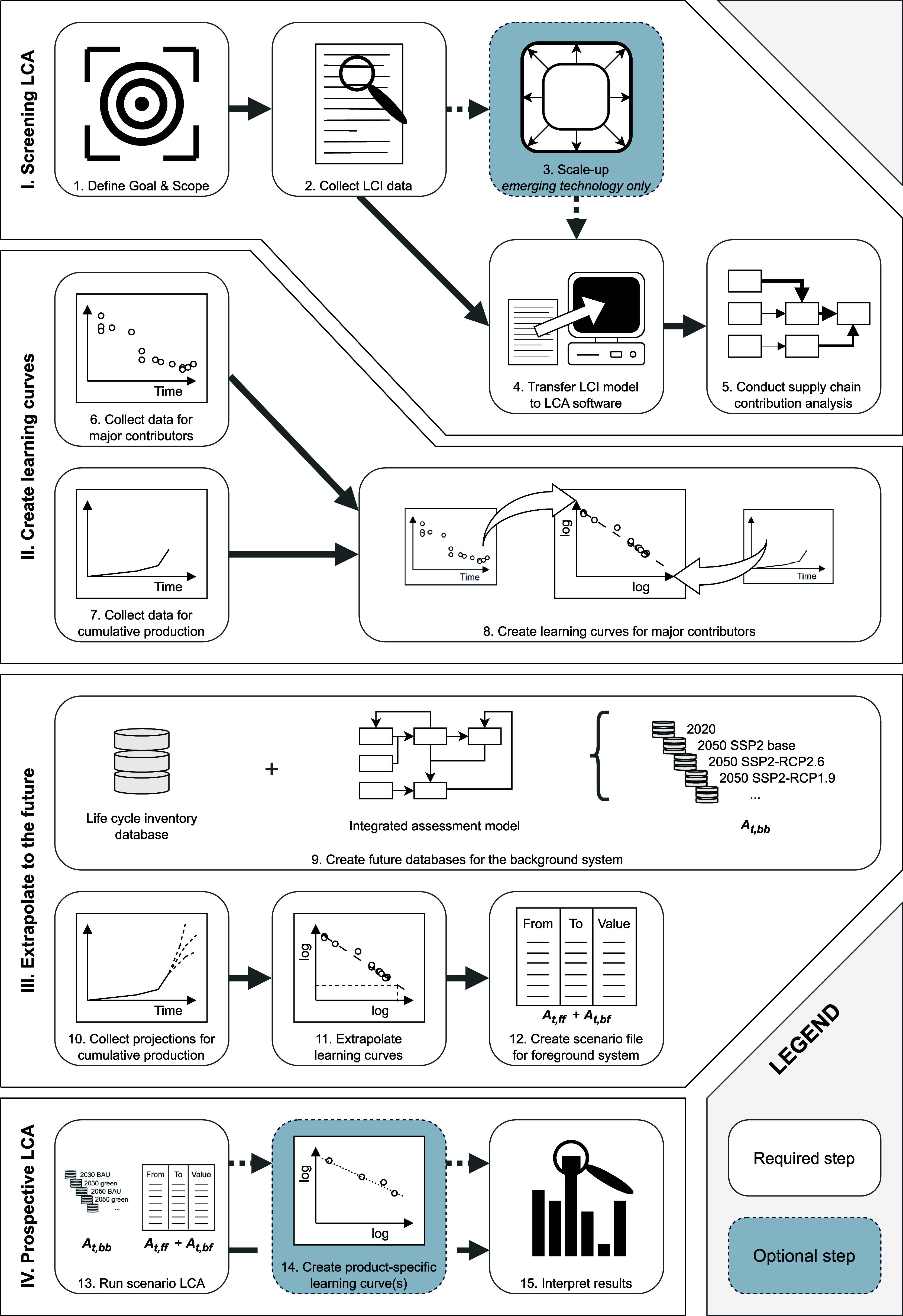
Flowchart of the proposed method for application of learning in foreground and background processes in prospective LCA.

**Table 1:** Description and Modeling Methods and Tools for Each Steps of the Proposed Method for Application of Learning in Foreground and Background Processes in Prospective LCA

	description	modeling methods and tools
Phase I. Screening LCA		
step 1. define goal and scope	define the goal, functional unit, system boundary, and temporal and geographic scope of the LCA and select the life cycle impact assessment method(s) and LCA software tools for conducting the LCA.	see guidelines for general LCA, e.g., ISO 14040 ^16^ and ISO 14044 ^17^
step 2. collect LCI data for the studied technology	use most recent LCI data for production of the studied technology.	LCI databases, (gray) literature, interviews with technology experts, etc.
step 3. scale-up the technology to a mature state (optional)	when the goal is to assess an emerging technology, the present LCI should first be upscaled using prospective LCA methods to model this technology at a future state that enables industrial production.	see existing frameworks presented in literature, e.g., ref 3, 13, 18–23
step 4. transfer the LCI model to LCA software	using the collected LCI data, create a database of the foreground system for use in LCA software that is compatible with the modeling methods in steps 5, 12, and 13.	Brightway^24^ and Activity Browser^25^ are most suitable at present
step 5. conduct a supply chain contribution analysis	identify contributing elements with a substantial contribution to the environmental impact of interest to direct available time and resources to data collection and learning curve creation for these elements.	e.g., *print_recursive_calculation* function of bw2analyzer in brightway2 ^24^
Phase II. Create Learning Curves		
step 6. collect time-series data for the major contributing elements in the supply chain	quantify trends in products used per activity (e.g., mass of glass/solar panel) for each major contributing element. When unavailable, use proxy data (e.g., glass thickness × area × density/solar panel). Collect as many data points for as wide a time range as possible to improve accuracy of the learning curve.	LCI databases, (gray) literature, statistics bureaus, trade organizations, researcher institutes, companies, trend reports, roadmaps, etc.
step 7. collect time-series data for the cumulative production	use cumulative production as a measure of learning. Alternatively, metrics such as cumulative units sold, cumulative units shipped, or cumulative installed capacity could be used.	
step 8. create learning curves for the major contributing elements	plot time-series data collected in step 6 against corresponding cumulative production data collected in step 7, both on log_10_ transformed axes. Fit a straight line through ordinary least-squares regression. Determine the slope β_*j*,*i*_, intercept *a*_*j*,*i*,0_, and confidence interval of each learning curve.	Wright’s law (eq 1)
Phase III. Extrapolate to the Future		
step 9. create future databases for the background system	apply projections from integrated assessment models (IAMs) to the LCI database used in modeling the background system. Include diverse development narratives from the IAMs for scenario analyses.	*premise*^15^ is most suitable at present
step 10. collect projection data for the cumulative production	where possible, collect projections for multiple years and diverse scenarios.	IAMs, roadmaps, trend reports, white papers, etc.
Step 11. extrapolate the learning curves	sample the confidence interval of the learning curves from step 8 through Monte Carlo simulations to estimate future values for the major contributing elements (e.g., mass glass/solar panel in 2050).	Wright’s law (eq 1) Monte Carlo simulations
step 12. create scenarios for the foreground system	identify for each major contributor the activity name, reference product, location, category, database, and key of the activity sending a reference product *j* (e.g., glass) and the activity *i* receiving that reference product (e.g., production of the solar panel).	Brightway^24^ or Activity Browser^25^ and a spreadsheet program (e.g., Excel)
Phase IV. Prospective LCA		
step 13. run a scenario LCA	combine the scenario for the foreground system with its corresponding background system.	Brightway^24^ or Activity Browser^25^
step 14. create product-specific learning curve(s) (optional)	LCA results for multiple years could be combined to create a learning curve for the technology.	Wright’s law (eq 1)
step 15. interpret the results	analyze how the results are impacted by developments in the foreground and background. Ideally study these developments both combined and separately (e.g., only background, only foreground, only one of the learning curves, etc.). Assess how uncertainty in the learning curves of each flow is transferred to the uncertainty in the environmental footprint of the product.	sensitivity and variance contribution analyses

In phase I, the goal and scope of the study are determined, and a screening LCA is conducted to identify, which parts of the product life cycle are major contributors to the studied environmental impacts of the product system. This helps prioritize time and resource deployment to learning curves for parts with a large contribution to assessed environmental impacts to capture the most significant learning-induced changes in these environmental impacts.

In phase II, learning curves for the identified major contributing parts are created. Data need to be collected for both cumulative production and changes affecting these elements over time. We followed the mathematical framework presented by Bergesen and Suh^10^ to introduce process-specific learning curves for foreground processes. Bergesen and Suh^10^ argued that the quantity *a* of product *j* (e.g., glass) going into activity *i* (e.g., cadmium telluride panel production) can reduce over time *t* as a function of cumulative production cp and an empirically determined process-specific learning parameters β following Wright’s law (eq 1), where *a*_*j*,*i*,*t*_ are elements of a time-resolved technosphere matrix ***A***_***t***_.

1 $$\log\;a_{j,i,t2}=\log\;a_{j,i,t1}+\beta_{j,i}\times \log\left({cp}_{i,t2}/{cp}_{i,t1}\right)$$

Learning parameter β is typically transformed and reported as the learning rate (LR = 1–2^β_*j*,*i*_^), which is the percentage change in the environmental indicator for each doubling of cumulative production. The technosphere matrix can be subdivided such that $A_{t}=\left[\begin{array}{cc} A_{t,ff} & A_{t,fb}\\ A_{t,bf} & A_{t,bb} \end{array}\right]$ with ***A***_***t***, ***ff***_ and ***A***_***t***, ***bb***_ containing all elements where the produced product and consuming activity are both part of the foreground or background system, respectively, and with ***A***_***t***, ***bf***_ containing all elements where products from the background system are used in activities in the foreground system and ***A***_***t***, ***fb***_ containing all elements where products from the foreground system are used in activities in the background system.

In phase III, the process-specific learning curves are applied to the foreground system, and the IAM projections are applied to the LCI database representing the background system to derive future values for the elements of the time-resolved technosphere matrix ***A***_***t***_. Our method applies eq 1 to the elements *a*_*j*,*i*,*t*_ of ***A***_***t***, ***ff***_ and ***A***_***t***, ***bf***_ and uses *premise* ^15^ to apply projections from an IAM to the elements *a*_*j*,*i*,*t*_ of ***A***_***t***, ***bb***_ that are covered by this IAM. Integration of the product into the background system is not considered, as this would require knowledge of market dynamics, which is currently considered outside the scope. Therefore, no elements *a*_*j*,*i*,*t*_ of ***A***_***t***, ***fb***_ are defined. To account for the uncertainty in the learning curves, Monte Carlo simulations are used to draw multiple samples from the confidence interval of the learning curves at a future point in time.

In phase IV, these sampled values for processes in the foreground system are combined with a future version of the background system in a time-specific LCA. Obtained results are interpreted through scenario and sensitivity analyses. When LCA results for multiple future years are created, they can be combined to create product-specific learning curves for the studied technology.

### 2.2 Case Study Application

#### 2.2.1 Phase I: Screening LCA

##### 2.2.1.1 Step 1—Goal and Scope

The goal of the case study was to demonstrate the application of our method, which assessed various environmental impacts. Additionally, we wanted to compare our results with those obtained using alternative methods based on learning curves. We therefore studied monocrystalline silicon PV panel production to enable comparison with Louwen et al.^6^ We focused on passivated emitter and rear cell (PERC), which at present has the largest market share in the monocrystalline PV market.^26^ The geographic scope was China, where the majority of PV panels are currently produced.^26^ The temporal scope comprised 2020 and 2050, with 2050 being the furthest point in time in the most recent World Energy Outlook (WEO) of the International Energy Agency.^27^ The functional unit was defined as “the production of 1 Watt-peak (W_p_) of PERC solar panel capacity”, with transportation, installation, use, maintenance, and end-of-life waste treatment placed outside the system boundary.

Impacts were assessed in LCA software, Brightway2,^24^ with the life cycle impact assessment method, ReCiPe 2016.^28^ This method contains 18 midpoint and three endpoint impact categories. The endpoints provide insight into damage to the three areas of protection, human health, ecosystem quality, and resource scarcity, by aggregating effects from the midpoints into single units. To ensure compatibility with background databases created by *premise* in step 9, several characterization factors were added or edited in the *Climate Change* impact category in line with van der Hulst et al. 2024.^29^ Results in the main text represent outcomes obtained with the hierarchist (H) perspective of ReCiPe, which considers time frames and available data for which there is scientific consensus. Results for the other two perspectives, egalitarian and individualist, are provided in the Supporting Information, Sections 2.2 and 2.3.

##### 2.2.1.2 Steps 2 and 3—Data Collection and Scale-Up

The foreground system was modeled using a recent and comprehensive LCI for the production of PERC panels provided in Müller et al.^30^ Since PERC is a mature technology, we did not apply upscaling to the LCI data as prescribed in optional step 3 of our method and therefore did not model any process changes, changes in the scale of product and equipment, or changes in known process synergies such as waste recycling. However, some adjustments were made to enable connection with the background database and to enable assessment of panels produced in China on the basis of a power-based functional unit in W_p_. A flowchart and comprehensive description of the studied product system are provided in the Supporting Information, Section 1.1.

##### 2.2.1.3 Steps 4—Transfer to LCA Software

A database file for the foreground system (provided in the data repository) was imported into Brightway2 using Activity Browser, version 2.9.7.^25,31^ The LCI database ecoinvent, version 3.9.1 system model “Allocation, cut-off by classification” ^32,33^ was used to model the background system. Through a midpoint-to-endpoint contribution analysis, we identified which midpoint categories contributed at least 15% to damage in either of the three endpoint categories.

##### 2.2.1.4 Step 5—Supply Chain Contribution Analysis

A supply chain contribution analysis was conducted for the major contributing impact categories using the *print_recursive_calculation* function of Brightway2.^24^ This function traverses the supply chain of the activity, producing the functional unit to identify processes with a substantial contribution to the environmental impact. Codes for application of the Brightway2 function are provided in the Supporting Information, Section 1.1.

#### 2.2.2 Phase II: Create Learning Curves

Time-series data for cumulative installed capacity and major contributors in the supply chain were collected from diverse publicly available data sources (Supporting Information, Section 1.2). In some cases, we found multiple data sources reporting different values for the same parameter and year. Data from official statistic bureaus were given preference and were only supplemented with data from other sources when using comparable modeling approaches and assumptions. Cumulative installed PV capacity was used as a measure of learning for all learning curves throughout the supply chain. Values for major environmental impact contributors were plotted against the cumulative installed capacity for the corresponding year on a log_10_–log_10_ plot. The slope β_*j*,*i*_ and intercept *a*_*j*,*i*,0_ of the learning curves were calculated with eq 1 through ordinary least-squares fitting of the log_10_ transformed data using linear models.

#### 2.2.3 Phase III: Extrapolate to the Future

##### 2.2.3.1 Step 9—Create Future Background Databases

Changes to the ecoinvent background database were based on projections from IMAGE,^34^ which is one of several IAMs available in *premise*. The IAM is given the parameters for a future scenario, and it will calculate for a number of sectors, which conditions need to be met to satisfy this future scenario. The future scenarios are informed by a combination of the shared socio-economic pathway (SSP) framework^35^ and the representative concentration pathway (RCP) framework.^36^ The former provides credible scenarios for the development of population, economic output, and rate of technological development, while the latter provides possible development scenarios for the level of GHGs. Projections for SSP2 were used, which is the “middle of the road” pathway for which social, economic, and technological developments are assumed to follow a trajectory similar to that charted by the past. Its baseline scenario results in a global mean surface temperature increase of 3–4 °C by 2100. Additionally, the RCP1.9 scenario was considered, which projects 1.9 W/m^2^ of radiative forcing from GHGs in 2100, coinciding with a global mean surface temperature increase of 1.2–1.4 °C, which is compatible with the Paris climate accord.^36^ We used *premise*, version 2.0.2 ^15,37^ to make adjustments to the sectors of electricity generation, transportation by truck, and production of fuels, steel, and cement to meet these scenarios. A copy of ecoinvent was created by *premise* for both scenarios for the year 2050. In addition, the year 2020 was included to enable comparison between projected and observed values. A more detailed account of the application of *premise* is provided in the Supporting Information, Section 1.3.

##### 2.2.3.2 Step 10—Projection of Cumulative Production

Since the IMAGE model projects changes to the energy market, it includes projections on the deployment of PV. We acquired the modeled projections for the cumulative installed capacity in each considered scenario from the IMAGE model developers. These projections were 728 GW in 2020 and 4644 and 8222 GW in 2050 for the SSP2-base and SSP2-RCP1.9 scenarios, respectively. For sensitivity analyses, we also included cumulative installed capacities for 2050 projected in the 2023 WEO,^27^ which were considerably higher than what was modeled by IMAGE.

##### 2.2.3.3 Steps 11 and 12—Extrapolate Learning Curves and Create Scenario Files

For each of the 11 learning curves and each scenario, the deterministic values for 2020 and 2050 were obtained by extrapolating the learning curves to the projected cumulative installed capacities from step 10 using eq 1 and the slope β_*j*,*i*_ and intercept *a*_*j*,*i*,0_ from step 8. To quantify how uncertainty in the learning curves parameters propagated into the LCA predicted impact, we simulated 1000 potential combinations of learning curve outcomes per scenario following a Monte Carlo approach. We limited the simulation to 1000 samples as this already required considerable computational power, taking on average about half an hour to assess impacts in 14 impact categories of only one future scenario. This procedure took the uncertainty in the estimated learning curve parameters slope β_*j*,*i*_ and intercept *a*_*j*,*i*,0_, to account for the covariances between the uncertainty in the estimated parameters by using Cholesky decomposition of the variance–covariance matrix. Deterministic and probabilistic values were stored in scenario files (see data repository) for use in the Activity Browser.

#### 2.2.4 Phase IV: Prospective LCA

##### 2.2.4.1 Step 13 and 14—Run Scenario LCA and Create Product-Specific Learning Curve(s)

The *Scenario LCA* feature of Activity Browser was used to calculate environmental impacts for major contributing midpoint impact categories and for all scenarios considered. Supporting Information, Section 1.4 provides a more detailed account of the application of this feature by using the provided files. Optional step 14 of creating product-specific environmental learning curves was not included, since impacts were calculated only for 2020 and 2050, and learning curves created from two data points are unlikely to be reliable.

##### 2.2.4.2 Step 15—Interpret Results

To assess which future developments (i.e., foreground learning curve projections or background conditions) lead to the largest reduction in environmental impacts, we conducted a one-at-a-time (OAT) sensitivity analysis. We conducted an LCA including only developments in the foreground (i.e., including all learning curves), only developments in the background, and both. All future permutated LCA results (2050) were compared to one current LCA result (2020) to quantify the sensitivity of the relative reduction in the estimated impact. We also performed OAT sensitivity analyses for the individual learning curves by one-at-a-time “switching off” the learning curve variables, replacing the value predicted in 2050 with the value derived for 2020. In addition, following the uncertainty propagation, the spearman rank correlation coefficients were used to quantify the influence of each of the learning curve estimates on the uncertainty in the estimated LCA impact.

## 3 Results

### 3.1 Phase I: Screening LCA

The contribution analysis revealed the most relevant elements of the system under study to orient data collection in subsequent steps. Five midpoint impact categories attributed 15% or more of the impacts in the three endpoint categories: *Climate Change*, *Acidification*, *Non-Carcinogenic Human Toxicity*, *Particulate Matter Formation*, and *Fossil Resource Scarcity* (see Supporting Information, Section 2.1). The process contribution analysis of the GHG footprint for the *Climate Change* impact category is visualized as a Sankey diagram in Figure 2 (for other categories, see Supporting Information, Section 2.1).

**Figure 2:**
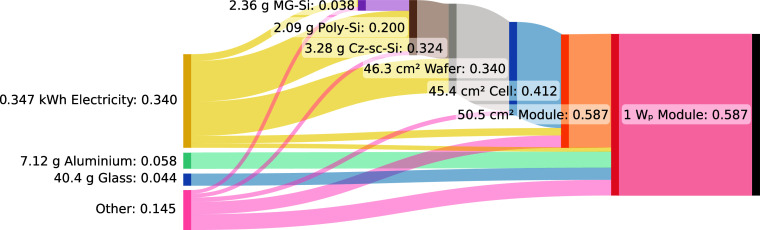
Sankey diagram of the supply chain for the production of 1 W_p_ of PERC solar panel capacity. Values behind each colon represent the GHG footprint of that product in kg CO_2_-eq per *W*_p_. MG-Si: metallurgical grade silicon; poly-Si: poly silicon; Cz-sc-Si: Czochralski single-crystalline silicon.

Here, we see processes contributing 2.5% or more to the total GHG footprint of the reference product (shown on the right). Each step toward the left represents a step up the supply chain. The leftmost processes represent processes in the background system. The category “Other” includes all background processes contributing less than 2.5% to the total GHG footprint. The reference product “Module” in *W*_p_ has 100% of its GHG footprint coming from the process “Module” in cm^2^. This is to be expected, since they are the same process but with a different unit. The required module area per W_p_ is determined by the module efficiency, which is therefore a major parameter to include in the model. One step further in the supply chain, we see that the cell, aluminum, glass, and electricity are the major contributors to the GHG footprint per area of “Module” produced. Therefore, input quantities for these materials and energy are major parameters to include in the model. Repeating this for all levels of the modeled supply chain and for all five relevant midpoint impact categories, we identified the following major contributors on which to focus data collection efforts:

- Module production (*W*_p_): module production (m^2^);
- Module production (m^2^): silicon cell, aluminum, copper, glass, ethylene-vinyl acetate, and electricity;
- Silicon cell production: silicon wafer, silver in metallization paste, and electricity;
- Silicon wafer production: Czochralski silicon;
- Czochralski single-crystal silicon production: polysilicon and electricity;
- Poly silicon production: metallurgical grade silicon and electricity;
- Metallurgical grade silicon production: electricity.

### 3.2 Phase II: Create Learning Curves

Time-series data were found for 11 of the 17 processes identified in Phase I (see Supporting Information, Section 1.2). The resulting process-specific learning curves are presented as dark gray lines in Figure 3. Module efficiency (Figure 3a) has a learning curve with a positive slope and, therefore, a negative LR, meaning that the efficiency increases with increasing cumulative installed capacity. The remaining ten processes have learning curves with negative slopes and positive learning rates and are thus decreasing with increasing cumulative installed capacity, e.g., the more panels produced, the more the thickness of the wafer, kerf, and glass decreases. The steeper the slope, the larger the LR, and thus the stronger the parameter increases or decreases as a result of learning.

**Figure 3:**
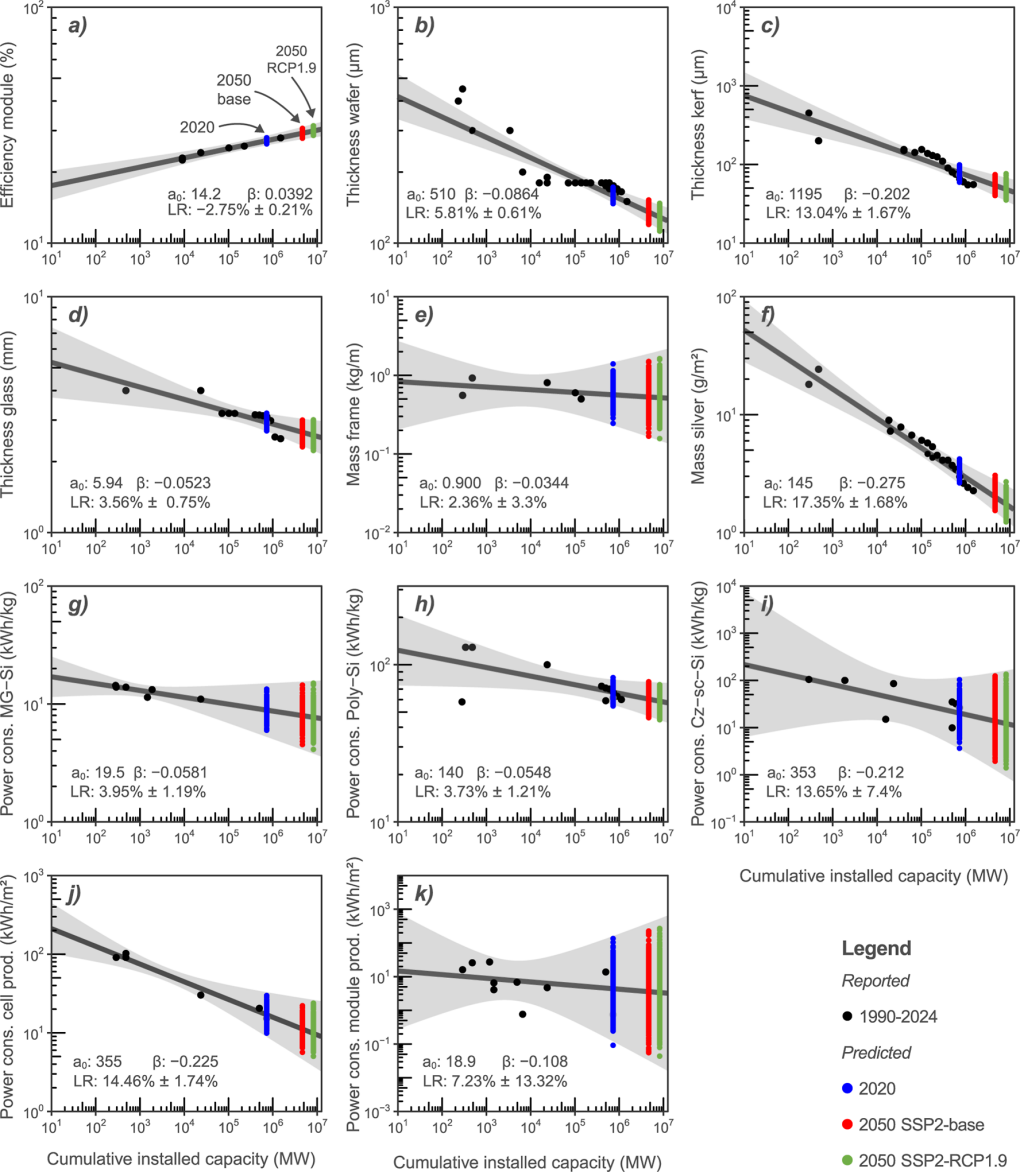
11 process-specific learning curves for which data were obtained. Individual empirical observations are represented with black dots. Cumulative installed capacities, as well as these reported observations were log_10_ transformed to enable ordinary least-squares fitting. The dark line represents the fitted learning curve and the shaded area represents the 95% confidence intervals, quantifying the uncertainty in the learning curve fit. Colored dots represented sampled values obtained through Monte Carlo simulation. *a*_0_: intercept, β: slope; LR: learning rate; cons.: consumption; prod.: production; MG-Si: metallurgical grade silicon; poly-Si: poly silicon; Cz-sc-Si: Czochralski single-crystalline silicon; SSP: shared socio-economic pathway; and RCP: representative concentration pathway.

### 3.3 Phase III: Extrapolate to the Future

The 1000 sampled values from the Monte Carlo simulation for each future scenario are represented with colored dots in Figure 3. Blue dots represent sampled values for 2020, using the cumulative installed capacity reported for that year. Red and green dots represent sampled values for 2050 based on, respectively, SSP2-base and SSP2-RCP1.9 projections of cumulative installed capacity for 2050. Narrower confidence intervals result in more tightly grouped sampled values.

### 3.4 Phase IV: Prospective LCA

Figure 4 displays a reproduction of the product-specific learning curve for the GHG footprint of mono-Si solar panels from Louwen et al.^6^ Projected GHG footprints, obtained using process-specific learning curves for PERC panels, are superimposed as three colored violin plots representing the distribution of the 1000 GHG footprints predicted using a Monte Carlo approach for each of the three assessed scenarios. The 2020 and 2050 SSP2-base scenarios, with average projected footprints of 0.474 and 0.314 kg CO_2_-equiv/*W*_p_, respectively, are in close agreement with the 0.499 and 0.274 kg CO_2_-equiv/*W*_p_ projected by Louwen et al. Thus, our approach using process-specific learning curves projects GHG footprint, which are in reasonable agreement with projections from product-specific learning curves. The more progressive SSP2-RCP1.9 scenario resulted in an average projected footprint in 2050 of 0.093 kg of CO_2_-equiv/*W*_p_, which is substantially lower than the 0.228 kg of CO_2_-equiv/*W*_p_ projected by Louwen et al., as would be expected. The SSP2-RCP1.9 scenario assumes extensive application of renewable energy, which would be a major shift away from past trends, whereas the SSP2-base scenario is closer to an extrapolation of the historic rate of progress in energy systems. It should be noted that the basis of comparison is slightly different, since Louwen et al. consider a system boundary that includes balance-of-system, whereas our projections do not account for this. Based on contribution analyses using ecoinvent data sets, the balance-of-system would account for approximately 25% of the GHG footprint. Extending our system boundary might help identify further similarities and differences in results obtained with both methods, but this was considered outside the scope of this study.

**Figure 4:**
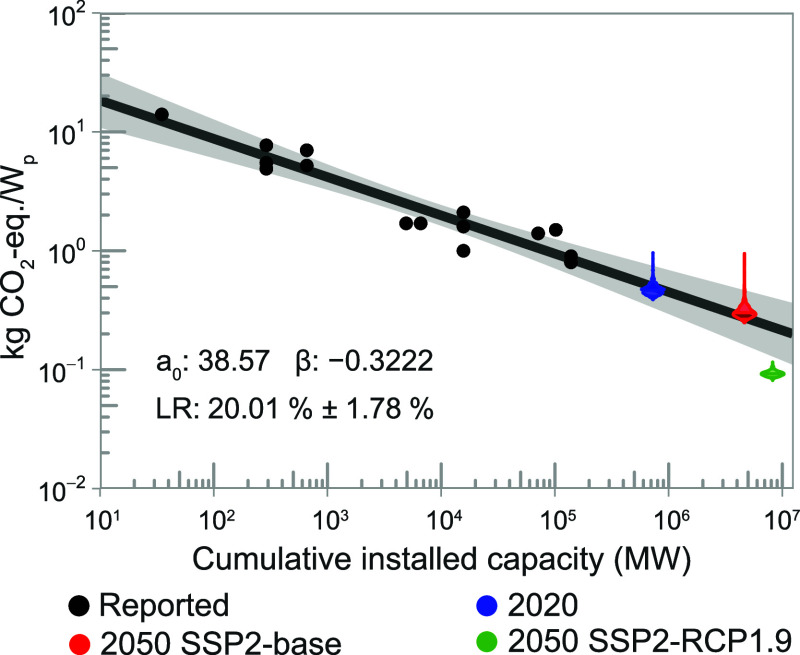
Results for the ReCiPe 2016 (H) climate change impact category. Empirical learning curve based on Louwen et al.^6^ for the GHG footprints of monocrystalline silicon PV systems reported in the literature, with colored violin plots superimposed that represent the values predicted using process-specific learning curves for the foreground and IAM projections for the background. kg CO_2_-equiv: kilogram carbon dioxide equivalent; *W*_p_: Watt-peak; MW: megawatt; *a*_0_: initial GHG footprint; β: learning parameter; LR: learning rate; SSP: shared socio-economic pathway; and RPC: representative concentration pathway.

Figure 5 shows the results of the OAT sensitivity analysis of the GHG footprint. Results for the other midpoint impact categories are provided in the Supporting Information, Section 2.1. We found that, in the SSP2-base scenario, learning in the foreground (F) reduces the GHG footprint by 28%, while changes in the background (B) reduce the GHG footprint by 12%. The combination (F + B) results in a 36% reduction in GHG footprint. Note how the reductions are not additive, due to interactions between the foreground and background systems. In the SSP2-RCP1.9 scenario, learning in the foreground (F) reduces the GHG footprint by 35%, while changes in the background (B) reduce the GHG footprint by 75%. The combination (F + B) results in a 80% reduction in GHG footprint. Thus, learning in the foreground is more important in the SSP2-base scenario, whereas changes in the background are more important in the SSP2-RCP1.9 scenario. Decarbonization of electricity is the development in the background with the largest contribution to GHG footprint reductions,^5^ while for the foreground system this is learning in module efficiency (see Supporting Information, Section 2.1.2). Note that the difference in results for only learning in the foreground (F) is due to different projections for the cumulative installed capacity in 2050. The SSP2-RCP1.9 scenario projects higher cumulative installed capacities, leading to more learning and therefore a larger reduction in the GHG footprint. Results for the OAT sensitivity analyses of the individual process parameters in the foreground system are provided in the Supporting Information, Section 2.1.2.

**Figure 5:**
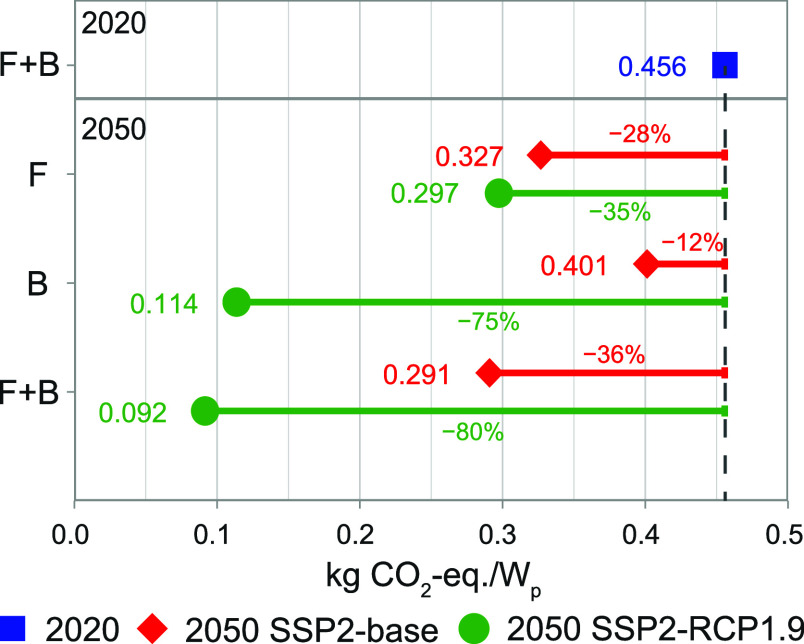
One-at-a-time sensitivity analyses showing how sensitive the impact reductions between 2020 and 2050 are to modeled developments in only the foreground system (F), only the background system (B), or both (F + B). kg CO_2_-equiv: kilogram carbon dioxide equivalent; *W*_p_: Watt-peak; F: foreground; B: background; SSP: shared socio-economic pathway; and RPC: representative concentration pathway.

Figure 6 displays the Spearman’s rank correlation coefficients between the GHG footprint and each of the 11 parameters for which process-specific learning curves were created. Results for the other midpoint impact categories are provided in the Supporting Information, Section 2.1. The higher the Spearman’s rank correlation coefficient of a process parameter, the more it contributes to the uncertainty in the GHG footprint of PERC panel production. Module efficiency has a negative correlation coefficient, meaning that increasing module efficiency correlates with decreasing GHG footprints. The other process parameters all have positive correlation coefficients, meaning that a decrease in these parameter values correlates with a decrease in GHG footprints. We found that the process-specific learning curves with the largest uncertainty in model fit, power consumption in Czochralski silicon, and module production, contribute the most to uncertainty in the GHG footprint. When electricity is decarbonized (i.e., SSP-RCP1.9), the GHG footprints of power consumption-related processes diminish. Thus, while the uncertainty in the model fit remains large for power consumption in Czochralski silicon and module production, their impact on the GHG footprint is reduced. Consequently, the process-specific learning curve for the frame becomes the major contributor to the uncertainty. Uncertainty in process-specific learning curves can be further reduced by, e.g., collecting more data points or further disaggregating these learning curves into multiple underlying process-specific learning curves.

**Figure 6:**
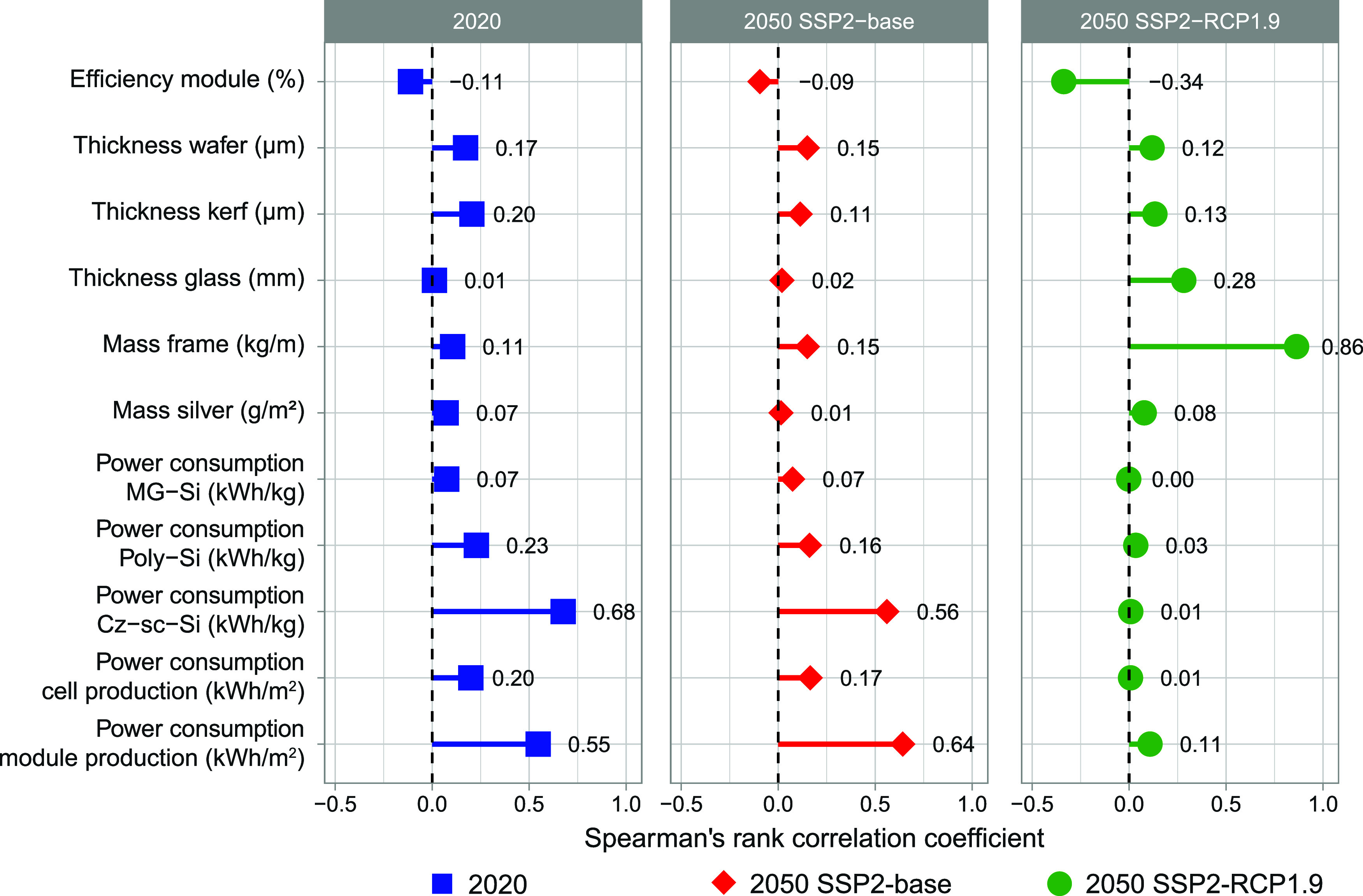
Uncertainty analyses showing the Spearman’s rank correlation coefficients relating the 1000 GHG footprints obtained for each scenario against the 11 process parameters adapted in the foreground system using learning curves. SSP: shared socio-economic pathway; RPC: representative concentration pathway; MG-Si: metallurgical grade silicon; poly-Si: poly silicon; and Cz-sc-Si: Czochralski single-crystalline silicon.

The other four assessed midpoint impact categories follow similar trends as displayed for *Climate Change* in Figure 5 and Figure 6, with the exception of *Non-Carcinogenic Human Toxicity* (see Supporting Information, Section 2.1.4). Emissions of heavy metals from mining of aluminum, copper, and silver are the main contributors, which are not affected by electricity decarbonization. Thus, in a decarbonized economy (i.e., SSP-RCP1.9), uncertainty in the mass of the frame and silver are major contributors to the uncertainty in this impact category. Copper was by far the major contributor, but changes in impacts from copper consumption were neither included in the foreground system for lack of data nor in the background system for lack of coverage of this sector by IMAGE. Thus, projected results for this category, as well as its contribution to the human health endpoint, are likely to be overestimated. This should be addressed in future research, for example, by expanding the scope of data collection to try and derive process-specific learning curves for the copper supply chain.

## 4 Discussion

### 4.1 Uncertainties

Our study shows for the first time how expected changes in foreground and background processes can be combined in prospective LCA. Our method is, however, not without uncertainties, which are further reflected upon below.

First, the single-factor learning curve used in the method attributes all observed changes in environmental performance over time to learning-by-doing. In economics, two- and multifactor learning curves are also used to try to more accurately correlate trends to multiple factors, such as learning-by-searching and economies of scale. Such comprehensive learning curves are yet to be applied to environmental impacts.^5^ Further research is required to assess which (set of) parameter(s) would be the best predictor(s) of changes in environmental impacts. More advanced two- and multifactor learning curves do require more time and effort, which might make their application less feasible to some practitioners.^5^

Second, learning curves require data for a measure of learning, such as cumulative production, which can introduce errors. To accurately derive this parameter at any point in time, one needs production data for all years of production. This can be difficult for early production years. Exclusion of these years could result in substantial underestimation of the cumulative production, as demonstrated by, among others, Weiss et al.^38^ When cumulative production data are absent altogether, one might have to rely on proxy data that can introduce further errors. In our case study, we used cumulative installed capacity of solar panels, which, among others, disregards panels that have been removed from the field at end-of-life. As more and more panels come offline, this proxy will become less and less reliable. However, solar panel production is exponentially increasing, and panels have a lifetime of around 30 years; thus, newly installed capacity far exceeds the capacity coming offline. This problem only becomes apparent once panel production starts deviating from its exponential growth path, in which case it would be advised to use best available estimates for cumulative production as a more reliable measure of learning.

Third, the use of a single measure of learning for the entire value chain of the foreground system might introduce errors. Data collection for the measure of learning was challenging in our case study. Therefore, we used cumulative installed capacity of solar panels as a measure of learning in all learning curves throughout the supply chain. While representative as measure of learning for processes close the functional unit (e.g., module and cell production), it is likely less representative for processes further up- or downstream. For example, metallurgical grade silicon is used in other products than solar panels, and its learning is thus affected by demand of multiple products. When reporting learning curves, it should therefore be clear what measure of learning was used, and data sets for both axes should be provided (as is done here), so that the learning curves can be tailored to the goal and scope of individual studies.

Fourth, extrapolation of learning curves requires projections for the measure of learning, which might vary between models and data sources. Assessment of the data quality of these models or data sources might require expert consultation. When no expert can be consulted and when one remains uncertain about the data quality of projections, it is preferred to use projections from various sources where available to get a sense of how much the differences in models or data sources may influence the results obtained from extrapolation. For example, using the 2023 WEO projections for cumulative installed capacity in 2050 returned lower average GHG footprints for 2050 of 0.087 kg CO_2_-equiv/*W*_p_ (see Supporting Information, Section 2.1.2), compared to using projections from IMAGE. Furthermore, we found that each subsequent WEO projects higher cumulative installed capacities, thus implying that these projections have thus far been chronically underestimated (see Supporting Information, Section 2.1.2).

Fifth, learning curves might not continue forever either because a new technology displaces an incumbent or because physical or practical limits are reached. As an example of displacement, PERC is projected to be fully displaced by other silicon PV technologies by 2034.^39^ Here, we assumed that these newer technologies are comparable, such that the process-specific learning curve for PERC can be considered representative for any silicon PV technology. As an example of reaching limits, the PV efficiency of a single-junction solar panel cannot exceed the Shockley–Queisser limit of 33.7% due to laws of physics.^40^ In such cases, Bergesen and Suh^10^ propose to restrict *a*_*j*,*i*,*t*_ to its limit value. This would result in learning curve plots, which suddenly flatten at the limit value.^41^ Alternatively, the limit value could be set as a limit value in curve fitting, with the learning curve fitted to approach, but never reach this limit value, as demonstrated by Ramírez and Worrell.^42^

Finally, separately integrating learning in the foreground and background might introduce discrepancies. For example, in our case study, developments in solar panels used in the foreground were managed by process-specific learning curves, while developments in solar panels used in the background system were managed by narratives for learning modeled in the applied IAM. The latter entailed a lower level of detail, and therefore we created two unique narratives for the same technology. This temporal inconsistency can be counteracted by integration of the foreground system into the background system. This requires identification and quantification of relevant elements *a*_*j*,*i*,*t*_ in the ***A***_***t***, ***fb***_ domain of time-resolved technosphere matrix ***A***_***t***_. Such an extension was, however, considered outside the scope of this study.

### 4.2 Challenges and Limitations

The presented method is particularly useful in prospective LCA of mature technologies but poses some challenges and limitations when applied to emerging technologies. Consider, for instance, silicon/perovskite tandem solar panels, which are an emerging technology that is likely to replace the mature technology assessed in the case study. Presently available lab and pilot scale data would first need to be upscaled (i.e., step 3 of the method) to be representative of a mature, emerged technology produced at an industrial scale (i.e., TRL 9). Subsequently applying learning curves to this upscaled emerged technology requires data of processes for which no historic data exist. For example, no historic data are available for production of the tandem panel. However, learning curves could be applied to the supply chain. For example, the same data from the case study might be used to model the silicon portion of the tandem panel’s supply chain. The perovskite component is again a part for which no historic data will be available. Therefore, learning curves would need to be applied to processes further up or down the supply chain. For example, while it might not be possible to apply learning curves to production of the perovskite layer or the complex chemicals used in the process, it might be possible to apply learning curves to production of the base chemicals used to create these complex chemicals. Thus, depending on the type of technology and its maturity, learning curves can be applied to processes close to the reference product, or they might need to be applied further up or down its supply chain. While in the latter case, the effects from learning are likely to be underestimated, it would present an improvement over the status quo, which often completely leaves out such supply chain learning effects. In the case of emerging technologies that require many new processes and materials, the presented method may not be appropriate, and other approaches may need to be considered.

### 4.3 Outlook

Our developed method to integrate learning in foreground and background processes within prospective LCA provides much needed practical guidance on how to create and apply environmental learning curves for a foreground system and on how to combine their use with prospective LCA databases for the background system created with, e.g., *premise*. While the results of our case study are in agreement with empirical observations, more case studies are required to further verify to what extent our method provides representative projections for future environmental impacts. In particular, the method should be tested on disparate technologies from a diverse set of sectors and using a broad selection of impact categories. Preferably, technologies beyond energy systems should be studied, in particular, technologies related to the production of base materials such as metals, fuels, concrete, plastics, and base chemicals. Much like with the creation of LCI databases such as ecoinvent, focusing on these base materials would provide the building blocks that enable the assessment of more complicated systems.

The creation and application of environmental learning curves are resource- and time-intensive. However, given their reproducible nature, learning curves can be updated or adapted for use in other case studies. Much like the development of LCI databases for use in conventional LCA, learning curve databases for use in prospective LCA could, over time, reduce the effort in applying learning curves. Thus, learning curves present an objective and scalable method for generating data points for the future. To aid in application and prevent redundant work, a central repository should be created to store collected data in a shared knowledge base. Not only should this repository contain learning rates but also the underlying data for both the *x*-axis (e.g., cumulative production) and *y*-axis (e.g., resource consumed or waste emitted per functional unit). This makes it possible to reproduce and verify the learning curves as well as extend them by adding new data points once they become available.

An important further development is the integration of learning curves for foreground systems into background databases, e.g., through tools such as *premise* ^15^ or *lca_algebraic*.^43^ This can be useful not only in prospective LCA but also in conventional LCA. Conventional LCI databases are typically created using peer-reviewed empirical data, which can quickly become outdated. Learning curves based on historical data could be applied as an aid in the annual update of these LCI databases. When used in prospective LCI databases, projections should ideally adhere to the narratives of the SSP and RCP frameworks, which endure broad consensus in the scientific community for use in scenario building. Such cross-compatibility of modeling approaches would also enable the IAM community to adjust their models based on insights from prospective LCA.

## Supplementary Material



## Data Availability

The data underlying this study are openly available in figshare at https://doi.org/10.6084/m9.figshare.7945955.

## Notes

Additional information regarding applied methods and additional results (PDF)
