# Correction to “Learning Curves in Prospective Life Cycle Assessment”

**DOI:** 10.1021/acs.est.5c18726

**Published:** 2026-01-08

**Authors:** Mitchell K. van der Hulst, Mara Hauck, Selwyn Hoeks, Rosalie van Zelm, Mark A. J. Huijbregts

The original publication contained an incorrect URL in the Data Availability Statement and Supporting Information. The correct URL is 10.6084/m9.figshare.c.7945955.

## Supplementary Material



## Data Availability

The data underlying this study are openly available in figshare at 10.6084/m9.figshare.c.7945955.

## Notes

Additional information regarding applied methods and additional results (PDF)

