# Supplementary material for: Learning Curves in Prospective Life Cycle Assessment
Source: Environ Sci Technol. 2025 Jul 30;59(31):16501–12. doi: 10.1021/acs.est.5c03870 (PMC12355957; doi:10.1021/acs.est.5c03870)
Supplement: Supplementary file 1 [file es5c03870_si_001.pdf]

Supporting Information

Learning Curves in Prospective Life Cycle Assessment

Mitchell K. van der Hulst[a,b], Mara Hauck[b,c], Selwyn Hoeks[a], Rosalie van Zelm[a], Mark A.J. Huijbregts[a,b,*]

[a] Department of Environmental Science, Radboud Institute for Biological and Environmental Sciences, P.O. Box 9010, 6500 GL, Nijmegen, The Netherlands
[b] Expertise Group Circularity, Sustainability, Impact, TNO, P.O. Box 80015, 3508 TA, Utrecht, The Netherlands
[c] Technology, Innovation & Society, Department of Industrial Engineering & Innovation Sciences, Eindhoven University of Technology, P.O. Box 513, Eindhoven, 5600 MB, The Netherlands

* Mark.Huijbregts@ru.nl

This Supporting Information provides a comprehensive description of collection and processing of data, as well as a providing additional result. Section 1 provides additional information regarding applied methods and section 2 provides additional results. Additional data are provided in spreadsheets and additional code is provided in jupyter notebook and R files which are made available in the Supplementary Information on figshare at https://doi.org/10.6084/m9.figshare.7945955.

This file contains:
- 62 pages
- 30 figures
- 20 tables

1. METHODS

1.1. Phase I: Screening LCA

A flow chart of the studied product system is presented in Fig. S1. The foreground system is largely defined by activities for which datasets were obtained from the study by Müller et al. [1] for industrial production of passivated emitter and rear contact (PERC) solar panels in China. All activities in the foreground system use products from the background system, which was represented using a version of the ecoinvent 3.9.1. database [2, 3] that was adjusted with *premise* version 2.0.2 [4, 5]. Datasets for the activities of aluminium alloy and flat glass production used in PERC module production were copied from the *premise* adjusted ecoinvent 3.9.1 database and manually adjusted so that electricity in these activities would be coming from Chinese electricity markets. Finally, one process was created in this work to convert the unit of the activity for module production from square meters (m^2) to Watt-peak (W_p), resulting in a process which produces our functional unit (in bold) as product. A spreadsheet file containing this foreground system is provided in the Supplementary Information on figshare. The foreground database can be reproduced by loading this file to a Brightway2 project using Activity Browser.

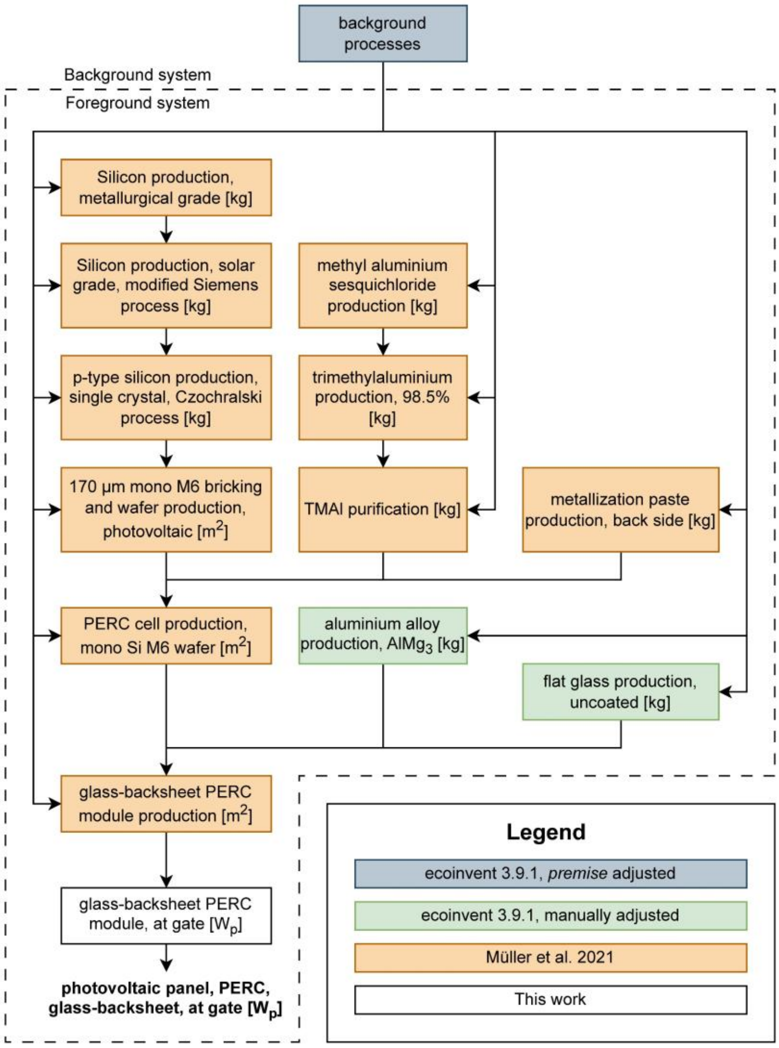

Fig. S1. *Flow chart of the product system considered in the case study of this work. PERC: Passivated Emitter and Rear Cell; TMAl: trimethylaluminium; AlMg*$_3$*: .aluminium-magnesium alloy.*

The *print_recursive_calculation* function of brightway2 [6] was used to conduct a process contribution analysis for the production of 1 W_p of PERC solar panel. One can set the calculation depth, which is the number of steps that the function traverses up or down the supply chain before it stops. It was set to seven, which is one larger than the length of the value chain of our foreground system as presented in the flow chart of Fig. S1. As a result, we obtain insight into the contributions of all processes in the foreground system, as well as the processes in the background system which contribute most to these processes in the foreground system. One can also set a cut-off, where impacts for flows that contribute less than this cut-off value are not printed. The ideal cut-off value depends on the product system and is a balancing act between keeping the list of contributors manageable and accounting for as much of the total impact as possible. Through trial and error, we settled on a cut-off of 2.5% for our product system. Using this cut-off value, at least 70% of impact are explained for all categories, meaning that the category "Other" makes up no more than 30% of the total impact.

Use of the *print_recursive_calculation* function of brightway2 [6] required installation of the following packages:

- bw2analyzer = 0.10
- bw2calc = 1.8.2
- bw2data = 3.6.6
- bw2io = 0.8.12

The database "Foreground" was loaded to a project named "learning_curves". In a Jupyter notebook, the following code was executed before executing the *print_recursive_calculation*.

```
import bw2data as bd
import bw2analyzer as ba
import bw2calc as bc
import bw2io as bi

bd.projects.set_current('learning_curves')

foreground_database = bd.Database('Foreground')

activity=[act for act in foreground_database if 'glass-backsheet PERC module, at gate' in act['name']][0]
```

The contribution analysis for the climate change impact category of ReCiPe 2016 (H) was conducted by running the code below:

```
lcia_method=[m for m in bd.methods if 'ReCiPe 2016 v1.03, midpoint (H) - premise' in m[0]
                  and 'global warming potential' in str(m)][0]

ba.print_recursive_calculation(activity, tuple(lcia_method), max_level=7, cutoff=0.025, tab_character="*")
```

The contribution analysis for the acidification impact category of ReCiPe 2016 (H) was conducted by running the code below:

```
lcia_method=[m for m in bd.methods if 'ReCiPe 2016 v1.03, midpoint (H) - premise' in m[0]
                  and 'acidification' in str(m)][0]

ba.print_recursive_calculation(activity, tuple(lcia_method), max_level=7, cutoff=0.025, tab_character="*")
```

The contribution analysis for the human non-carcinogenic toxicity impact category of ReCiPe 2016 (H) was conducted by running the code below:

```
lcia_method=[m for m in bd.methods if 'ReCiPe 2016 v1.03, midpoint (H) - premise' in m[0]
                  and 'human toxicity: non-carcinogenic' in str(m)][0]

ba.print_recursive_calculation(activity, tuple(lcia_method), max_level=7, cutoff=0.025, tab_character="*")
```

The contribution analysis for the particulate matter formation impact category of ReCiPe 2016 (H) was conducted by running the code below:

```
lcia_method=[m for m in bd.methods if 'ReCiPe 2016 v1.03, midpoint (H) - premise' in m[0]
                  and 'particulate matter formation' in str(m)][0]

ba.print_recursive_calculation(activity, tuple(lcia_method), max_level=7, cutoff=0.025, tab_character="*")
```

The contribution analysis for the non-renewable fossil energy resources impact category of ReCiPe 2016 (H) was conducted by running the code below:

```
lcia_method=[m for m in bd.methods if 'ReCiPe 2016 v1.03, midpoint (H) - premise' in m[0]
                  and 'energy resources: non-renewable, fossil' in str(m)][0]

ba.print_recursive_calculation(activity, tuple(lcia_method), max_level=7, cutoff=0.025, tab_character="*")
```

The contribution analysis for the climate change impact category of ReCiPe 2016 (I) was conducted by running the code below:

```
lcia_method=[m for m in bd.methods if 'ReCiPe 2016 v1.03, midpoint (I) - premise' in m[0]
                  and 'global warming potential' in str(m)][0]

ba.print_recursive_calculation(activity, tuple(lcia_method), max_level=7, cutoff=0.025, tab_character="*")
```

The contribution analysis for the acidification impact category of ReCiPe 2016 (I) was conducted by running the code below:

```
lcia_method=[m for m in bd.methods if 'ReCiPe 2016 v1.03, midpoint (I) - premise' in m[0]
                  and 'acidification' in str(m)][0]

ba.print_recursive_calculation(activity, tuple(lcia_method), max_level=7, cutoff=0.025, tab_character="*")
```

The contribution analysis for the photochemical oxidant formation: terrestrial impact category of ReCiPe 2016 (I) was conducted by running the code below:

```
lcia_method=[m for m in bd.methods if 'ReCiPe 2016 v1.03, midpoint (I) - premise' in m[0]
                  and 'photochemical oxidant formation: terrestrial' in str(m)][0]

ba.print_recursive_calculation(activity, tuple(lcia_method), max_level=7, cutoff=0.025, tab_character="*")
```

The contribution analysis for the particulate matter formation impact category of ReCiPe 2016 (I) was conducted by running the code below:

```
lcia_method=[m for m in bd.methods if 'ReCiPe 2016 v1.03, midpoint (I) - premise' in m[0]
                  and 'particulate matter formation' in str(m)][0]

ba.print_recursive_calculation(activity, tuple(lcia_method), max_level=7, cutoff=0.025, tab_character="*")
```

The contribution analysis for the non-renewable fossil energy resources impact category of ReCiPe 2016 (I) was conducted by running the code below:

```
lcia_method=[m for m in bd.methods if 'ReCiPe 2016 v1.03, midpoint (I) - premise' in m[0]
                  and 'energy resources: non-renewable, fossil' in str(m)][0]

ba.print_recursive_calculation(activity, tuple(lcia_method), max_level=7, cutoff=0.025, tab_character="*")
```

The contribution analysis for the climate change impact category of ReCiPe 2016 (E) was conducted by running the code below:

```
lcia_method=[m for m in bd.methods if 'ReCiPe 2016 v1.03, midpoint (E) - premise' in m[0]
                  and 'global warming potential' in str(m)][0]

ba.print_recursive_calculation(activity, tuple(lcia_method), max_level=7, cutoff=0.025, tab_character="*")
```

The contribution analysis for the ecotoxicity: marine impact category of ReCiPe 2016 (E) was conducted by running the code below:

```
lcia_method=[m for m in bd.methods if 'ReCiPe 2016 v1.03, midpoint (E) - premise' in m[0]
                  and 'ecotoxicity: marine' in str(m)][0]
```

```
ba.print_recursive_calculation(activity, tuple(lcia_method), max_level=7, cutoff=0.025, tab_character="*")
```

The contribution analysis for the human non-carcinogenic toxicity impact category of ReCiPe 2016 (E) was conducted by running the code below:

```
lcia_method=[m for m in bd.methods if 'ReCiPe 2016 v1.03, midpoint (E) - premise' in m[0]
                    and 'human toxicity: non-carcinogenic' in str(m)][0]

ba.print_recursive_calculation(activity, tuple(lcia_method), max_level=7, cutoff=0.025, tab_character="*")
```

The contribution analysis for the non-renewable fossil energy resources impact category of ReCiPe 2016 (E) was conducted by running the code below:

```
lcia_method=[m for m in bd.methods if 'ReCiPe 2016 v1.03, midpoint (E) - premise' in m[0]
                    and 'energy resources: non-renewable, fossil' in str(m)][0]

ba.print_recursive_calculation(activity, tuple(lcia_method), max_level=7, cutoff=0.025, tab_character="*")
```

1.2. Phase II: Create learning curves

Each learning curve consists of an x-axis that described a measure of learning (e.g. cumulative production of the functional unit) and a y-axis that describes a learning dependant parameter (e.g. kg steel/functional unit). For the product system of our case study, we identified eleven learning dependant parameters, for which data collection and transformation is described in subsequent sections. An overview of data sources is provided in Tab. S1 and the individual datapoints for each of the eleven learning dependant parameters are provided in an spreadsheet which is available in the Supplementary Information on figshare. The following sections describe the data sources and data transformations for use in process-specific learning curves in more detail.

Tab. S1. *Time-series data for major contributors in the supply chain of producing 1 Watt-peak (W_p) of PERC solar panel capacity. EVA: ethylene-vinyl acetate; MG-Si: metallurgical grade silicon; poly-si polycrystalline silicon; Cz-sc-si: Czochralski single-crystal silicon.*

**Dependent parameter**			
**Process,** Major contributor	**Representing variable**	**Time series**	**Source(s)/comment**
**Module production (W_p)**			
Module consumption (m^2)	photovoltaic efficiency (%)	2007, 2009, 2012, 2015, 2023, 2024	[7]
**Module production (m^2)**			
Silicon cell consumption	m^2 cell/m^2 module	-	Changes were negligible
Aluminum consumption	kg aluminium/meter frame	1992, 1996, 2009, 2013, 2013	[8-11]
Copper consumption	kg copper/m^2 module	-	No data were found
Glass consumption	mm glass/m^2 module	1996, 2009, 2011–2013, 2017–2023	[8-19]
EVA consumption	kg EVA/m^2 module	-	No changes over time observed
Electricity consumption	kWh/m^2 module	1992, 1996, 2000, 2001, 2005, 2006, 2009, 2018	[8, 9, 20]
**Silicon cell production**			
Silicon wafer consumption	m^2 wafer/m^2 cell	-	No changes over time observed
Silver consumption (in metallization paste)	mg silver/m^2 cell	1992, 1996, 2009 - 2023	[8-19, 21-24]
Electricity consumption	kWh/m^2 cell	1992, 1996, 2009, 2018	[8, 9, 20]
**Silicon wafer production**			
Czochralski silicon consumption	µm wafer/m^2 wafer	1990, 1992, 2004, 2006, 2008–2023	[8-19, 21-26]
Czochralski silicon consumption	µm kerf/m^2 wafer (i.e. wafer cutting loss)	1992, 1996, 2010–2023	[8-19, 21-24]
**Czochralski single-crystal silicon production**			
Poly-silicon consumption	kg poly-Si/kg Cz-sc-Si	-	No data were found
Electricity consumption	kWh/kg Cz-sc-Si	1992, 2002, 2008, 2009, 2018, 2019	[8, 9, 14, 15, 20, 27, 28]
**Poly-silicon production**			
Metallurgical grade silicon consumption	kg MG-Si/kg poly-Si	-	No data were found
Electricity consumption	kWh/kg poly-Si	1991, 1993, 1996, 2009, 2017–2022	[8, 9, 20, 29-33]
**Metallurgical grade silicon production**			
Electricity consumption	kWh/kg MG-Si	1991, 1992, 1996, 2001, 2002, 2009	[8, 9]

**Independent parameter**			
**Process**	**Representing variable**	**Time series**	**Source(s)/comment**
Learning-by-doing	MW cumulative installed capacity	1957-2022	[33-49]

1.2.1. Module efficiency

The area per kilowatt peak is inversely proportional to the photovoltaic efficiency of the panel. Trends in efficiency were obtained from NREL, with record research-cell efficiencies reported for 1977–2018 [50], while champion module efficiencies were only reported for 2007–2023 [51]. The rate of improvement in cell and module efficiency were found to be comparable, with record module efficiencies trailing behind the record cell efficiencies. While the record cell efficiency data table contains more entries, thus providing a more empiricial data on which to base the learning curve, we opted to use the record module efficiency table, since this would avoid the introduction of an error when converting record cell efficiencies to record module efficiencies

Learning in module efficiency was include in the scenario files by adjusting the amount of 'photovoltaic panel, PERC, glass-backsheet' from the activity 'glass-backsheet PERC module production' in square meters going into the activity 'glass-backsheet PERC module, at gate' to produce 'photovoltaic panel, PERC, glass-backsheet, at gate' in Watt-peak. The area per Watt-peak A_{W_p} was calculated with Eq. S1, where SI_{STC} is the solar irradiation and η_{STC} the effiency under standard test conditions (i.e. 1000 W/m^2, 25°C, air mass equal to 1.5 and ASTM G173-03 standard spectrum).

$$A_{W_p}\left[\frac{m^2}{W_p}\right] = \frac{0.001\left[\frac{kW_p}{W_p}\right]}{SI_{STC}\left[\frac{kW}{m^2}\right] * \eta_{STC}[-]} = \frac{0.001\left[\frac{kW_p}{W_p}\right]}{1\left[\frac{kW}{m^2}\right] * \eta_{STC}[\%] * 0.01[-/\%]} \qquad \text{Eq. S1}$$

1.2.2. Mass of silver

The mass of silver used in solar cell production was obtained from various sources. The amount of silver used in 1992 was calculated from the publicly available source documentation for energy systems modelled in ecoinvent 2 [8] using Eq. S2. The mass of silver used per square meter of cell ($m_{Ag,cell,'92}$) is calculated from the mass of solder paste ($m_{Lotpaste}$) and solder (m_{Lot}) used, the concentration of silver in the solder ($conc._{Ag}$), the number of cells per panel (n_{cells}) and the area of the cells per panel (A_{cells})

$$m_{Ag,cell,'92}\left[\frac{g}{m^2}\right] = \frac{\left(m_{Lotpaste}\left[\frac{g}{panel}\right] + m_{Lot}\left[\frac{g}{panel}\right]\right) * conc._{Ag}\,[\%]}{n_{cells}\left[\frac{1}{panel}\right] \Big/ A_{cells}\left[\frac{m^2}{panel}\right]} \qquad \text{Eq. S2}$$

The amount of silver used in 1996 also reported in the publicly available source documentation for energy systems modelled in ecoinvent 2 [8], where the area of mass of silver per cell was divided by the area of the cell.

The amount of silver used in 2009 was calculated from the publicly available source documentation for energy systems modelled in ecoinvent 3 [9] using Eq. S3. The mass of silver used per square meter of cell ($m_{Ag,cell,'09}$) is calculated from the mass of front side metallization paste ($m_{MP,front}$) and back side metallization paste ($m_{MP,front}$) used, and the mass of silver (m_{Ag}) used in both metallization pastes.

$$m_{Ag,cell,'09}\left[\frac{g}{m^2}\right] = \frac{m_{MP,front}}{cell}\left[\frac{g}{m^2}\right] * \frac{m_{Ag}}{m_{MP,front}}\left[\frac{kg}{kg}\right] + \frac{m_{MP,back}}{cell}\left[\frac{g}{m^2}\right] * \frac{m_{Ag}}{m_{MP,back}}\left[\frac{kg}{kg}\right] \quad \text{Eq. S3}$$

The amount of silver used in 2010 to 2023 was derived from the ITRPV roadmaps by dividing the mass of silver used per cell with the area of the cell.

1.2.3. Mass of frame

Aluminium is used in the frame of the module, with the length of frame fixed by the circumference of the module. Therefore, data were collected on the mass of aluminium consumed per meter of frame used in module production. Trends in mass of aluminium per meter of frame were obtained from openly available documentation of the ecoinvent database [8, 9], and VDMA roadmaps [10, 11].

Learning in mass of the frame is include in the scenario files by adjusting the amount of 'aluminium alloy, AlMg3' from the activity 'aluminium alloy production, AlMg3' in kilograms going into the activity 'glass-backsheet PERC module production' to produce 'photovoltaic panel, PERC, glass-backsheet' in square meters.

1.2.4. Glass thickness

For glass, the input mass per solar panel depends on the area and thickness of the glass. For one square meter of panel, the area is fixed, therefore, only data on the thickness of glass sheets used in module production are relevant. The input mass of glass has decreased over time as glass has progressively become thinner to reduce weight and cost. Trends in glass thickness were obtained from openly available documentation of the ecoinvent database [8, 9], as well as from market shares of different glass thicknesses reported in the VDMA roadmaps [10-18, 21-24, 26].

Learning in glass thickness is include in the scenario files by adjusting the amount of 'flat glass, uncoated' from the activity 'flat glass production, uncoated' and the amount of 'tempering, flat glass' from the activity 'market for tempering, flat glass', both in kilograms, going into the activity 'glass-backsheet PERC module production' to produce 'photovoltaic panel, PERC, glass-backsheet' in square meters. The mass of glass per area m_{glass} was calculated with Eq. S4, where Δ_{glass} is the thickness of the glass in mm and δ_{glass} is the density of glass, which is 2530 kg/m^3.

$$m_{glass}\left[\frac{kg}{m^2}\right] = \Delta_{glass}[mm] * 0.001\left[\frac{m}{mm}\right] * \delta_{glass}\left[\frac{kg}{m^3}\right] \quad \text{Eq. S4}$$

1.2.5. Wafer thickness

Czochralski single-crystal silicon consumption per square meter of wafer has decreased as wafers have become thinner. Trends in wafer thickness were obtained from Fraunhofer ISE [25], openly available documentation of the ecoinvent database [8, 9] and VDMA roadmaps [10-18, 21-24, 26].

For a description of how changes in learning in wafer thickness was included in the scenario files, see the next section.

1.2.6. Kerf thickness

Losses from cutting wafers from Czochralski single-crystal silicon ingots, known as kerfloss, have decreased over time in an effort to maximize the yield of wafers per ingot. The trend in saw thickness was obtained from openly available documentation of the ecoinvent database [8] and VDMA roadmaps [10-18, 21-24, 26].

Besides cutting losses in the z-axis of the wafer due to the thickness of the saw (i.e. kerfloss), cutting losses also occur in the x-y plane of the wafer as the cylindrical Czochralski single-crystal silicon ingot is cut into semi-square wafers. When cutting a square out of a circle, the maximum area is obtained when the diagonal of the square equals the diameter of the circle. However, a larger surface area for the wafer can be realized by cutting a semi-square with a slightly larger diagonal than the diameter of the circle, resulting in a square which is missing its corners. Cutting losses in the x-y were assumed to not have changed significantly over time on a per-area basis.

Learning in wafer and kerf thickness is include in the scenario files by adjusting the amount of 'silicon, single crystal, Czochralski' from the activity 'p-type silicon production, single crystal, Czochralski process' in kilograms, going into the activity '170 µm mono M6 bricking and wafer production, photovoltaic' to produce 'single-Si wafer, photovoltaic' in square meters. The mass of Czochralski single-crystal silicon per area $m_{poly-Si}$ was calculated with Eq. S5, where d_{ingot} is the diameter of an M12/G12 Czochralski single-crystal silicon ingot, which is 0.295 meters [52], Δ_{wafer} and Δ_{kerf} are the thickness of the wafer and kerf in µm, δ_{Si} is the density of silicon, which is 2330 kg/m^3, and A_{wafer} is the area of a semi-square G12 wafer, which is 44096 mm^2 [53].

$$m_{poly-Si}\left[\frac{kg}{m^2}\right] = \frac{\pi * \left(\frac{d_{ingot}\,[m]}{2}\right)^2 * \left(\Delta_{wafer}[\mu m] + \Delta_{kerf}[\mu m]\right) * 10^{-6}\left[\frac{m}{\mu m}\right] * \delta_{Si}\left[\frac{kg}{m^3}\right]}{A_{wafer}\,[mm^2] * 10^{-6}\left[\frac{m^2}{mm^2}\right]} \qquad \text{Eq. S5}$$

1.2.7. Power consumption in metallurgical grade silicon production

Electricity consumption in metallurgical grade silicon consumption has decreased over time in an effort to reduce cost of production. The trend in energy efficiency was obtained from openly available documentation of the ecoinvent database [8, 9].

Learning in power consumption in metallurgical grade silicon production is include in the scenario files by adjusting the amount of 'electricity, medium voltage' from the activity 'market group for electricity, medium voltage' from location 'CN' in kilowatt hours going into the activity 'silicon production, metallurgical grade' to produce 'silicon, metallurgical grade' in kilograms.

1.2.8. Power consumption in poly-silicon production

Electricity consumption in poly-silicon consumption has decreased over time in an effort to reduce cost of production. The trend in energy efficiency was obtained from openly available documentation of the ecoinvent database [8, 9], Woodhouse et al. [54] and trend reports from the IEA [29-33].

Learning in power consumption in poly-silicon production is include in the scenario files by adjusting the amount of 'electricity, medium voltage' from the activity 'market group for electricity, medium

voltage' from location 'CN' in kilowatt hours going into the activity 'silicon production, solar grade, modified Siemens process' to produce 'silicon, poly-silicon' in kilograms.

1.2.9. Power consumption in Czochralski single-crystal silicon production

Electricity consumption in Czochralski single-crystal silicon consumption has decreased over time in an effort to reduce cost of production. The trend in energy efficiency was obtained from openly available documentation of the ecoinvent database [8, 9], Jester [27], Vedde et al. [28], Woodhouse et al. [54] and VDMA roadmaps [14, 15].

Learning in power consumption in Czochralski single-crystal silicon production is include in the scenario files by adjusting the amount of 'electricity, medium voltage' from the activity 'market group for electricity, medium voltage' from location 'CN' in kilowatt hours going into the activity 'p-type silicon production, single crystal, Czochralski process' to produce 'silicon, single crystal, Czochralski' in kilograms.

1.2.10. Power consumption in cell production

Electricity consumption in cell consumption has decreased over time in an effort to reduce cost of production. The trend in energy efficiency was obtained from openly available documentation of the ecoinvent database [8, 9], and Woodhouse et al. [54].

Learning in power consumption in cell production is include in the scenario files by adjusting the amount of 'electricity, medium voltage' from the activity 'market group for electricity, medium voltage' from location 'CN' in kilowatt hours going into the activity 'PERC cell production, mono Si M6 wafer' to produce 'photovoltaic cell, single-Si wafer' in square meter.

1.2.11. Power consumption in module production

Electricity consumption in module consumption has decreased over time in an effort to reduce cost of production. The trend in energy efficiency was obtained from openly available documentation of the ecoinvent database [8, 9], and Woodhouse et al. [54].

Learning in power consumption in module production is include in the scenario files by adjusting the amount of 'electricity, medium voltage' from the activity 'market group for electricity, medium voltage' from location 'CN' in kilowatt hours going into the activity 'glass-backsheet PERC module production' to produce 'photovoltaic panel, PERC, glass-backsheet' in square meter.

1.2.12. Cumulative installed capacity

Cumulative installed capacity of PV panels in megawatts was used as the measure of learning. A time-series for the cumulative installed PV capacity between 1957 and 2022 was created by combining data from various sources. These include trends [33] and snapshot [49] reports from the IEA PVPS, global market outlooks by SolarPower Europe (formerly European Photovoltaic Industry Association)[43-47], and statics data by the United States Energy Information Administration (EIA) [48], the international renewable energy agency (IRENA)[38], and the energy institute (formerly BP)

[40-42]. Additional data were extracted from a book on semiconductors and semimetals [34] and from the source material [35-37, 39] of learning curves for the cost of PV as presented by Our World in Data [55] and the performance curve database by the Santa Fe Institute [56, 57]. The cumulative installed capacity for each year between 1957 and 2022 was calculated by taking the average of the values reported by the various sources.

1.3. Phase III: Extrapolate to the future

To be able to fully reproduce the work presented herein, one needs to recreate the prospective copies of the ecoinvent databases that were used. To do so, one needs to install a python package management software such as anaconda or miniconda, create an environment, and install the python packages *premise 2.0.2* [5] and *jupyter lab* in this environment. Once installed, one can open *jupyter lab* in their browser and run the jupyter notebook file that is provided in the Supplementary Information on figshare. An HTML version of the notebook is included to enable inspection of the code without the use of python. The jupyter notebook contains all further instructions for application of *premise 2.0.2* to ecoinvent 3.9.1. Running the code in the jupyter notebook file using *jupyter lab* will…

- …create a project named 'learning_curves',
- …set-up this project by installing the biosphere database and LCIA methods,
- …install the original ecoinvent 3.9.1 database,
- …install three prospective copies of the ecoinvent 3.9.1 database for the three scenarios considered herein (i.e. 2020 SSP2-base, 2050 SSP2-base, 2050 SSP2-RCP1.9).

Note that you need an active licence for the ecoinvent database, as well as a decryption key for *premise* which can be obtained from the library maintainers. Furthermore, note that older or newer versions of *premise* might give different outcomes. While it is advised to use the latest version for new research, you will require *premise 2.0.2* when trying to replicate the outcomes of the research presented herein.

1.4. Phase IV: Prospective LCA

Values for each scenario were stored in separate scenario files. Proxy values were converted to correct units for the flow based on the established relationship. For example, trends in thickness of the glass were converted to trends in mass of the glass through multiplication with the area and the density of glass. The learning curve for glass thickness affected two products going into the activity of module production, since both an input from the activity of producing glass and an input from the activity of tempering glass are required. The learning curves for wafer and kerf thickness were combined, since both affected the input of the product poly-silicon going into the activity of wafer production. Thus, in total, eleven products going into eleven activities were identified in each scenario file.

Changes in the foreground system are defined in scenario files, while changes in the background system are defined in either of the three copies of the ecoinvent LCI database created with *premise*. To reproduce our work, one needs to make specific combinations of background database and scenario files as listed in Tab. S2 to obtain the reported result. Using Activity Browser, one needs to (re)link the *Foreground* database to the listed background database and subsequently conduct a

scenario LCA loading the listed scenario file. The scenario files are provided as spreadsheets in the Supplementary Information on figshare.

Tab. S2. *Overview of which background database and foreground scenario file should be used for each scenario.*

**Background** - *Database name*	**Foreground** - *Scenario file name*
Regular prospective LCA	
**2020 -** *ei391_cutoff_SSP2_base_2020*	**2020 -** *deterministic_f_SSP2-base_2020_b_SSP2-base_2020.xlsx*
**2050 baseline -** *ei391_cutoff_SSP2_base_2050*	**2050 baseline -** *deterministic_f_SSP2-base_2050_b_SSP2-base_2050.xlsx*
**2050 RCP1.9 -** *ei391_cutoff_SSP2_RCP19_2050*	**2050 RCP 1.9 -** *deterministic_f_SSP2-RCP19_2050_b_SSP2-RCP19_2050.xlsx*
Assess only foreground changes	
**2020 -** *ei391_cutoff_SSP2_base_2020*	**2050 baseline -** *deterministic_f_SSP2-base_2050_b_SSP2-base_2020.xlsx*
**2020 -** *ei391_cutoff_SSP2_base_2020*	**2050 RCP 1.9 -** *deterministic_f_SSP2-RCP19_2050_b_SSP2-base_2020.xlsx*
Assess only background changes	
**2050 baseline -** *ei391_cutoff_SSP2_base_2050*	**2020 -** *deterministic_f_SSP2-base_2020_b_SSP2-base_2050.xlsx*
**2050 RCP1.9 -** *ei391_cutoff_SSP2_RCP19_2050*	**2020 -** *deterministic_f_SSP2-base_2020_b_SSP2-RCP19_2050.xlsx*
Monte Carlo simulation	
**2020 -** *ei391_cutoff_SSP2_base_2020*	**2020 -** *monte_carlo_f_SSP2-base_2020_b_SSP2-base_2020.xlsx*
**2050 baseline -** *ei391_cutoff_SSP2_base_2050*	**2050 baseline -** *monte_carlo_f_SSP2-base_2050_b_SSP2-base_2050.xlsx*
**2050 RCP1.9 -** *ei391_cutoff_SSP2_RCP19_2050*	**2050 RCP 1.9 -** *monte_carlo_f_SSP2-RCP19_2050_b_SSP2-RCP19_2050.xlsx*
Monte Carlo simulation – cumulative production assumptions from IEA 2023 WEO [58]	
**2020 -** *ei391_cutoff_SSP2_base_2020*	**2020 -** *monte_carlo_f_IEA-current2020_b_SSP2-base_2020.xlsx*
**2050 RCP1.9 -** *ei391_cutoff_SSP2_RCP19_2050*	**2050 IEA-NZE -** *monte_carlo_f_IEA-NZE2050_b_SSP2-RCP19_2050.xlsx*

2. RESULTS

2.1. ReCiPe 2016 midpoint hierarchist (H)

2.1.1. Midpoint-to-Endpoint contribution analysis

A midpoint-to-endpoint contribution analysis for the production of 1 W_p of PERC solar panel was conducted using the ReCiPe 2016 endpoint life cycle impact assessment method [59, 60]. Adjustment to the characterization factors of carbon dioxide, methane, and hydrogen in the impact category of Climate Change were made in line with van der Hulst et. al 2024 [61]. Tab. S3 displays contributions of the eighteen midpoint categories to the three end-point categories for the hierarchist perspective. Midpoint categories contributing 15% or more to the total impact of either of the three endpoint categories are highlighted in gray and were included in further assessments at the midpoint level.

Tab. S3. *Midpoint-to-endpoint contribution analysis for producing 1 Watt-peak (W_p) of PERC solar panel capacity using the ReCiPe 2016 hierarchist (H) impact assessment method.*

**Endpoint**, midpoint	**Unit**	**Impact per W_p**	**Contribution**
**Ecosystem quality**	**species*yr**	**2.96E-09**	**100%**
Acidification: terrestrial, terrestrial acidification potential (TAP)	species*yr	5.30E-10	18%
Climate change: freshwater, global warming potential (GWP)	species*yr	4.49E-14	0%
Climate change: terrestrial, global warming potential (GWP)	species*yr	1.65E-09	56%
Ecotoxicity: freshwater, freshwater ecotoxicity potential (FETP)	species*yr	5.28E-11	2%
Ecotoxicity: marine, marine ecotoxicity potential (METP)	species*yr	1.06E-11	0%
Ecotoxicity: terrestrial, terrestrial ecotoxicity potential (TETP)	species*yr	4.24E-11	1%
Eutrophication: freshwater, freshwater eutrophication potential (FEP)	species*yr	1.16E-10	4%
Eutrophication: marine, marine eutrophication potential (MEP)	species*yr	7.11E-14	0%
Land use, agricultural land occupation (LOP)	species*yr	2.20E-10	7%
Photochemical oxidant formation: terrestrial ecosystems, photochemical oxidant formation potential: ecosystems (EOFP)	species*yr	2.57E-10	9%
Water use, water consumption potential (WCP)	species*yr	3.98E-15	0%
**Human Health**	**DALY**	**1.59E-06**	**100%**
Climate change, global warming potential (GWP)	DALY	5.45E-07	34%
Human toxicity: carcinogenic, human toxicity potential (HTPc)	DALY	1.22E-07	8%
Human toxicity: non-carcinogenic, human toxicity potential (HTPnc)	DALY	2.38E-07	15%
Ionising radiation, ionising radiation potential (IRP)	DALY	2.06E-10	0%
Ozone depletion, ozone depletion potential (ODPinfinite)	DALY	1.07E-10	0%
Particulate matter formation, particulate matter formation potential (PMFP)	DALY	6.73E-07	42%
Photochemical oxidant formation: human health, photochemical oxidant formation potential: humans (HOFP)	DALY	1.69E-09	0%
Water use, water consumption potential (WCP)	DALY	1.46E-08	1%
**Natural resources**	**USD2013**	**3.86**	**100%**
Energy resources: non-renewable, fossil, fossil fuel potential (FFP)	USD2013	2.67E-02	86%
Material resources: metals/minerals, surplus ore potential (SOP)	USD2013	4.38E-02	14%

2.1.2. Climate Change

The output of the *print_recursive_calculation* is as follows:

```
Fraction of score | Absolute score | Amount | Activity
0001 | 0.5874 |     1 | 'glass-backsheet PERC module production' (watt peak, CN, None)
*0001 | 0.5874 | 0.005052 | 'glass-backsheet PERC module production' (square meter, CN, None)
**0.702 | 0.4125 | 0.004537 | 'PERC cell production, mono Si M6 wafer' (square meter, CN, None)
***0.579 | 0.3399 | 0.004627 | '170 µm mono M6 bricking and wafer production, photovoltaic' (square m
****0.551 | 0.3237 | 0.003276 | 'p-type silicon production, single crystal, Czochralski process' (kilo
*****0.34 | 0.1997 | 0.002093 | 'silicon production, solar grade, modified Siemens process' (kilogram,
******0.065 | 0.03817 | 0.002365 | 'silicon production, metallurgical grade' (kilogram, CN, None)
*******0.0433 | 0.02546 | 0.02602 | 'market group for electricity, medium voltage' (kilowatt hour, CN, Non
******0.251 | 0.1475 | 0.1507 | 'market group for electricity, medium voltage' (kilowatt hour, CN, Non
*******0.223 | 0.1308 | 0.1247 | 'market group for electricity, medium voltage' (kilowatt hour, CN-SGCC
*******0.0284 | 0.01671 | 0.02605 | 'market for electricity, medium voltage' (kilowatt hour, CN-CSG, None)
*****0.21 | 0.1231 | 0.1258 | 'market group for electricity, medium voltage' (kilowatt hour, CN, Non
******0.186 | 0.1092 | 0.104 | 'market group for electricity, medium voltage' (kilowatt hour, CN-SGCC
*******0.0501 | 0.02943 | 0.02382 | 'market for electricity, medium voltage' (kilowatt hour, CN-NCGC, None
*******0.0423 | 0.02485 | 0.02828 | 'market for electricity, medium voltage' (kilowatt hour, CN-ECGC, None
*******0.0391 | 0.02297 | 0.01542 | 'market for electricity, medium voltage' (kilowatt hour, CN-NECG, None
*******0.0273 | 0.01602 | 0.01536 | 'market for electricity, medium voltage' (kilowatt hour, CN-NWG, None)
***0.0456 | 0.02677 | 0.02736 | 'market group for electricity, medium voltage' (kilowatt hour, CN, Non
****0.0404 | 0.02374 | 0.02263 | 'market group for electricity, medium voltage' (kilowatt hour, CN-SGCC
**0.0996 | 0.05853 | 0.007125 | 'aluminium alloy production, AlMg3' (kilogram, CN, None)
***0.0878 | 0.0516 | 0.007125 | 'aluminium alloy production, AlMg3' (kilogram, RER, None)
****0.0679 | 0.0399 | 0.006875 | 'market for aluminium, cast alloy' (kilogram, GLO, None)
*****0.0608 | 0.03571 | 0.001797 | 'aluminium ingot, primary, to aluminium, cast alloy market' (kilogram,
******0.0537 | 0.03154 | 0.001396 | 'market for aluminium, primary, ingot' (kilogram, RoW, None)
*******0.0401 | 0.02356 | 0.0009819 | 'aluminium production, primary, ingot' (kilogram, CN, None)
**0.0755 | 0.04433 | 0.04042 | 'flat glass production, uncoated' (kilogram, CN, None)
***0.0708 | 0.04159 | 0.04042 | 'flat glass production, uncoated' (kilogram, RER, None)
**0.0279 | 0.01642 | 0.01677 | 'market group for electricity, medium voltage' (kilowatt hour, CN, Non
```

The Sankey diagram for this output is provided in Fig. 1 of the main text and is reproduced here in Fig. S2.

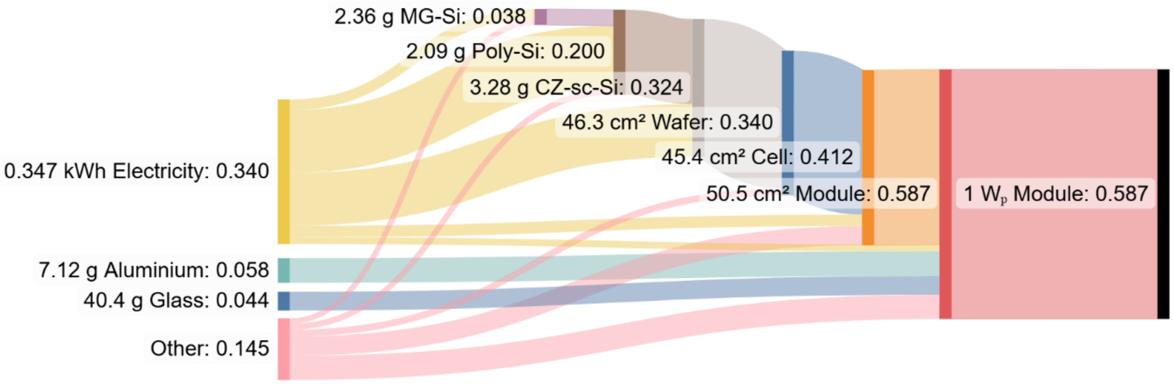

Fig. S2. *Sankey diagram of the supply chain for the production of 1 Watt-peak (W_p) of PERC solar panel capacity. Values behind each colon represent the GHG footprint of that product in kg CO_2-eq/W_p. MG-Si: metallurgical grade silicon; poly-Si: poly-silicon; Cz-sc-Si: Czochralski single-crystalline silicon.*

Louwen et al. [62] used future projections for the cumulative installed capacity from the 2014 World Energy Outlook (WEO) of the International Energy Agency (IEA) to extrapolate their learning curves to 2040. In the main text we used the projections for cumulative installed PV capacity in the IAM that was also used in projecting changes in the background. To assess the sensitivity of different assumptions in projecting future cumulative installed capacities, we additionally used the WEO. Fig. S3 contains the same empirical learning curve from Louwen et al. [62] as in Fig. 3 of the main text, but now it contains violin plots for the GHG footprints that were obtained when using projections for the cumulative installed capacity from the 2023 WEO of the IEA [58].

To ensure consistency in assumption between the foreground and background, scenarios were matched based on their narrative. In line with Boyce et al. [63], we matched the net zero emissions by 2050 (NZE) scenarios from the WEO with the SSP2-RCP1.9 scenarios. This resulted in considerably higher projections for the cumulative installed capacity in 2050 of 18,753 GW. No scenario from WEO matched the SSP2-base scenario. Since the WEO projects higher cumulative installed capacities for 2050, the violin plots for 2050 are shifted more to the right.

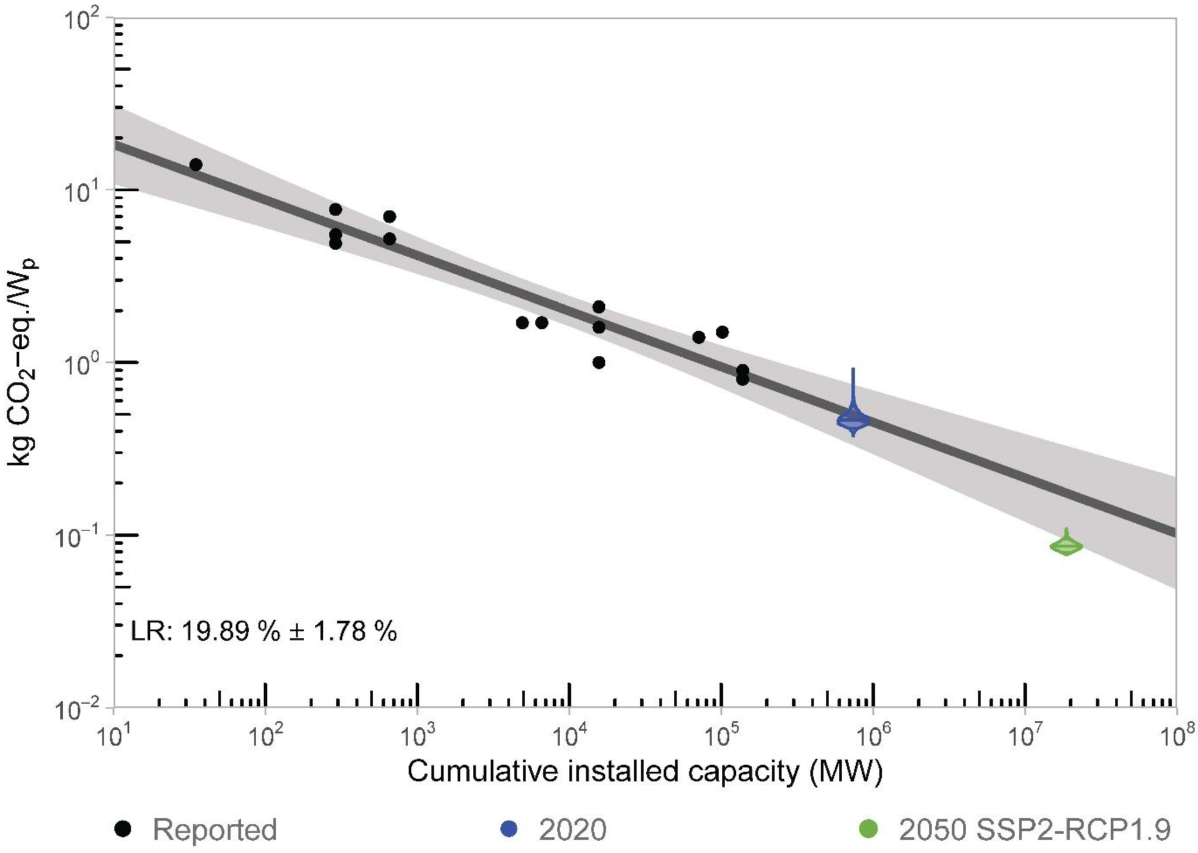

***Fig. S3**. Empirical learning curve based on Louwen et al. [62] for the GHG footprints of mono-crystalline silicon PV systems reported in literature, with colored violin plots superimposed that represent the values predicted using process-specific learning curves for the foreground and IAM projections for the background. The cumulative installed capacity projections for these scenarios are based on projections from the IEA [58].*

The cumulative production projected for 2050 in the 2023 WEO is a factor 2.3 higher than what is projected by IMAGE. However, even this higher projected might be underestimating the cumulative capacity that might be achieved in 2050. Fig. S4 contains the historic development of the cumulative

installed capacity of solar (black dots), as well as projections for the cumulative installed capacity from the IEA extracted from their world energy outlook published between 1998 and 2023 (coloured dots). The historic trend is one of exponential growth in cumulative installed capacity, with periods of weaker growth being followed by periods of stronger growth. As can be observed, projections for the cumulative installed capacity increase with every subsequent edition of the WEO, with projections consistently trending lower than what can be expected from extrapolating the historic exponential growth. The 2002 WEO is the first to project cumulative installed capacity for 2030 and projected this to be 56 GW. This projection has already been increased to 4.7 TW in the latest WEO. However, extrapolating the historic trend results in a projection of 21 TW. This discrepancy becomes even larger further into the future. The 2021 WEO was the first to contain projections for 2050, projecting a cumulative installed capacity of 6.2 TW. The latest WEO already increased this projection to 13 TW, while extrapolation of the historic trend would result in a projection of 9.2 EW. Even when projecting into the near future, the WEO under-projects the cumulative installed capacity. For example, the 2016 WEO was the last to project cumulative installed capacity in 2020, projecting it to be 0.42 GW, while the realised cumulative installed capacity in 2020 was 0.74 GW. Individual datapoints for Fig. S4 are provided in a spreadsheet which is available in the Supplementary Information on figshare.

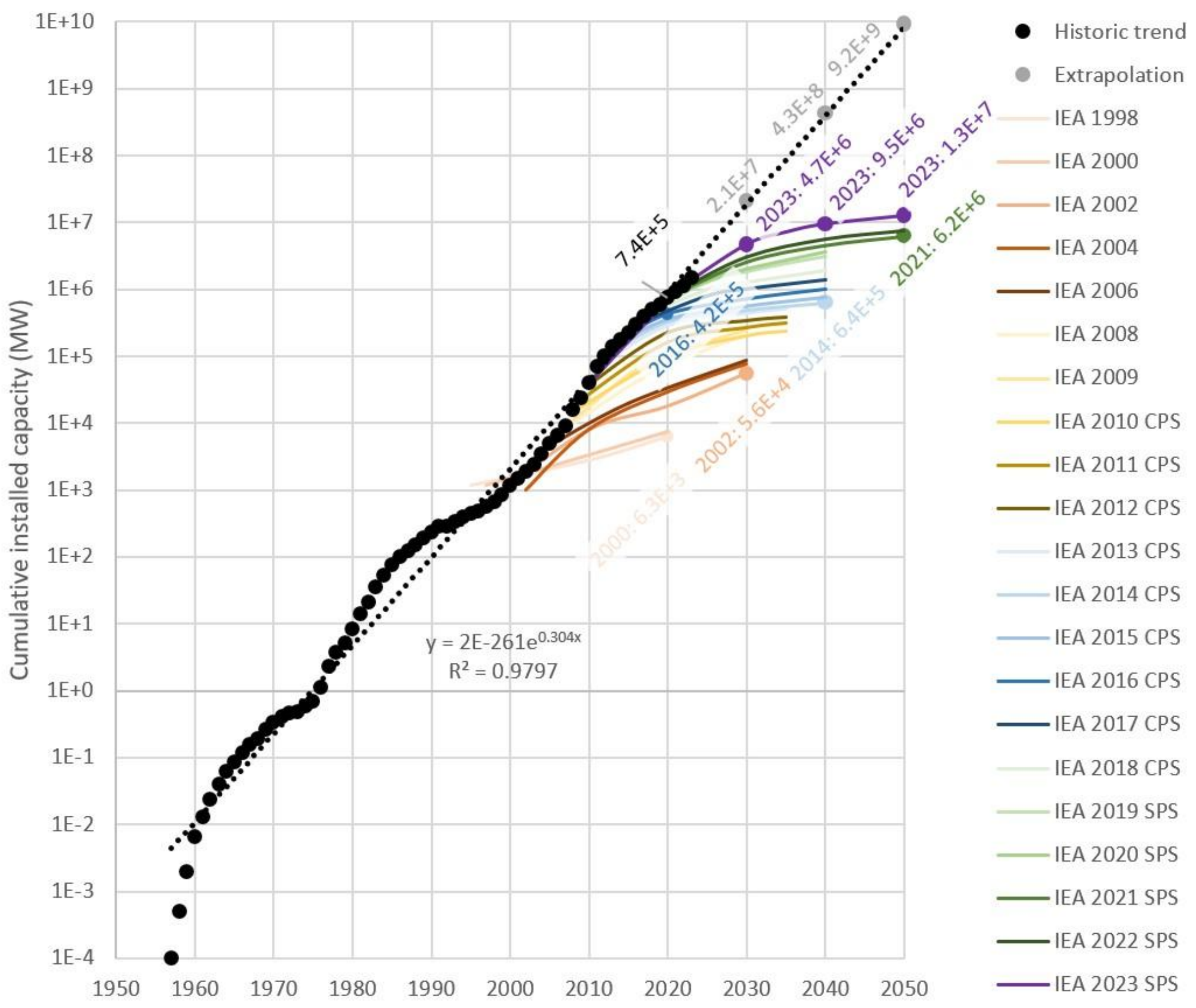

Fig. S4. *Historic trend in cumulative installed capacity of solar and projections for the development of the cumulative installed capacity as projected by the IEA in their WEO from 1998 to 2023.*

Eventually, growth in cumulative installed capacity will have to slow as market saturation is achieved, but it is difficult to project when this can be expected to occur. If cumulative installed capacity were to continue growing exponentially as it has for the past six decades, the latest projections for 2050 might be off by two orders of magnitude. At higher cumulative installed capacities, more learning will have occurred and therefore environmental footprints would be lower than what is projected in the main text. Several of the process-specific learning curves may reach their theoretical limit value well before reaching cumulative installed capacities in the order of petawatts. For sustained improvement in environmental impacts, other technologies would need to be employed which are not restricted by the same theoretical limits as current technologies. A likely route would be the deployment of multi-junction solar panels, which can exceed the Shockley-Queisser limit. Thus it is likely that if cumulative installed capacity were to continue increasing exponentially, that multi-junction solar panels will start making up a larger portion of this capacity. This in turn decreases growth in the cumulative installed capacity of mono-crystalline silicon and will eventually terminate the learning curve for this technology, to be replaced with the learning curve for a new technology, being the multi-junction solar panels.

Results for the OAT sensitivity analyses of the ReCiPe 2016 (H) climate change midpoint impact category are provided in Tab. S4. The values in rows labelled F, B, and F+B correspond with values in Fig. 4 of the main text where the percentage change in GHG footprint is calculated between 2020 and 2050 ($\Delta_{2020 \rightarrow 2050}$). The final eleven rows represent OAT sensitivity values when excluding a single learning curve in the foreground system, where the percentage change in GHG footprint is calculated between "F+B" and "F+B, excl. [a single learning curve (e.g. efficiency)]". From Tab. S4 it becomes apparent that exclusion of learning in panel efficiency results in the largest increase in GHG footprint and, therefore, the GHG footprint is most sensitive to this process parameter.

Tab. S4. *One-at-a-time sensitivity analyses for the ReCiPe 2016 (H) climate change midpoint impact category showing how sensitive the percentage impact reductions between 2020 and 2050 are to modelled developments in only the foreground system (F), only the background system (B), or both (F+B), and to exclusion of individual learning curves for the foreground system.*

	**2020**
**Baseline**	**kg CO_2-eq.**
	0.455688338

	**2050 SSP2-base**		**2050 SSP2-RCP1.9**	
	**kg CO_2-eq.**	**$\Delta_{2020 \rightarrow 2050}$**	**kg CO_2-eq.**	**$\Delta_{2020 \rightarrow 2050}$**
**F**	0.32795799	−28%	0.297445703	−35%
**B**	0.401296988	−12%	0.113653425	−75%
**F+B**	0.290894535	−36%	0.091561130	−80%
	**kg CO_2-eq.**	**$\Delta_{FB \rightarrow FB\ excl.}$**	**kg CO_2-eq.**	**$\Delta_{2020 \rightarrow 2050}$**
**F+B, excluding efficiency**	0.312795393	7.5%	0.100683	10.0%
**F+B, excluding thickness wafer**	0.306648064	5.4%	0.094514	3.2%
**F+B, excluding thickness kerf**	0.307332436	5.7%	0.094553	3.3%
**F+B, excluding thickness glass**	0.294180469	1.1%	0.095175	3.9%
**F+B, excluding mass frame**	0.291900591	0.3%	0.092486	1.0%
**F+B, excluding mass silver**	0.292977244	0.7%	0.092158	0.7%
**F+B, excluding power consumption MG-Si**	0.292096493	0.4%	0.091580	0.02%
**F+B, excluding power consumption poly-Si**	0.298470448	2.6%	0.091681	0.13%
**F+B, excluding power consumption Cz-sc-Si**	0.302983724	4.2%	0.091744	0.20%
**F+B, excluding power consumption cell production**	0.306140791	5.2%	0.091807	0.27%
**F+B, excluding power consumption module production**	0.293232508	0.8%	0.091600	0.04%

2.1.3. Acidification

The output of the *print_recursive_calculation* is as follows:

```
Fraction of score | Absolute score | Amount | Activity
0001 | 0.002501 |     1 | 'glass-backsheet PERC module production' (watt peak, CN, None)
*0001 | 0.002501 | 0.005052 | 'glass-backsheet PERC module production' (square meter, CN, None)
**0.553 | 0.001382 | 0.004537 | 'PERC cell production, mono Si M6 wafer' (square meter, CN, None)
***0.463 | 0.001159 | 0.004627 | '170 µm mono M6 bricking and wafer production, photovoltaic' (square m
****0.428 | 0.001071 | 0.003276 | 'p-type silicon production, single crystal, Czochralski process' (kilo
*****0.261 | 0.0006531 | 0.002093 | 'silicon production, solar grade, modified Siemens process' (kilogram,
******0.054 | 0.000135 | 0.002365 | 'silicon production, metallurgical grade' (kilogram, CN, None)
*******0.0343 | 8.585e-05 | 0.02602 | 'market group for electricity, medium voltage' (kilowatt hour, CN, Non
******0.199 | 0.0004973 | 0.1507 | 'market group for electricity, medium voltage' (kilowatt hour, CN, Non
*******0.175 | 0.000437 | 0.1247 | 'market group for electricity, medium voltage' (kilowatt hour, CN-SGCC
*****0.166 | 0.000415 | 0.1258 | 'market group for electricity, medium voltage' (kilowatt hour, CN, Non
******0.146 | 0.0003648 | 0.104 | 'market group for electricity, medium voltage' (kilowatt hour, CN-SGCC
*******0.0414 | 0.0001035 | 0.02382 | 'market for electricity, medium voltage' (kilowatt hour, CN-NCGC, None
*******0.0321 | 8.034e-05 | 0.02828 | 'market for electricity, medium voltage' (kilowatt hour, CN-ECGC, None
*******0.0292 | 7.304e-05 | 0.01542 | 'market for electricity, medium voltage' (kilowatt hour, CN-NECG, None
***0.0361 | 9.026e-05 | 0.02736 | 'market group for electricity, medium voltage' (kilowatt hour, CN, Non
****0.0317 | 7.933e-05 | 0.02263 | 'market group for electricity, medium voltage' (kilowatt hour, CN-SGCC
**0.0934 | 0.0002335 | 0.007125 | 'aluminium alloy production, AlMg3' (kilogram, CN, None)
***0.0839 | 0.0002098 | 0.007125 | 'aluminium alloy production, AlMg3' (kilogram, RER, None)
****0.0695 | 0.000174 | 0.006875 | 'market for aluminium, cast alloy' (kilogram, GLO, None)
*****0.0638 | 0.0001596 | 0.001797 | 'aluminium ingot, primary, to aluminium, cast alloy market' (kilogram,
******0.0547 | 0.0001369 | 0.001396 | 'market for aluminium, primary, ingot' (kilogram, RoW, None)
*******0.0405 | 0.0001012 | 0.0009819 | 'aluminium production, primary, ingot' (kilogram, CN, None)
**0.114 | 0.0002843 | 0.04042 | 'flat glass production, uncoated' (kilogram, CN, None)
***0.11 | 0.0002749 | 0.04042 | 'flat glass production, uncoated' (kilogram, RER, None)
**0.123 | 0.0003068 | 0.0007477 | 'market for copper, cathode' (kilogram, GLO, None)
***0.103 | 0.0002567 | 0.0004523 | 'electrorefining of copper, anode' (kilogram, GLO, None)
****0.101 | 0.0002523 | 0.000449 | 'market for copper, anode' (kilogram, GLO, None)
*****0.0343 | 8.584e-05 | 0.0001675 | 'smelting of copper concentrate, sulfide ore' (kilogram, RoW, None)
*****0.0386 | 9.655e-05 | 0.0001708 | 'smelting of copper concentrate, sulfide ore' (kilogram, CN, None)
```

The Sankey diagram for this output is provided in Fig. S5.

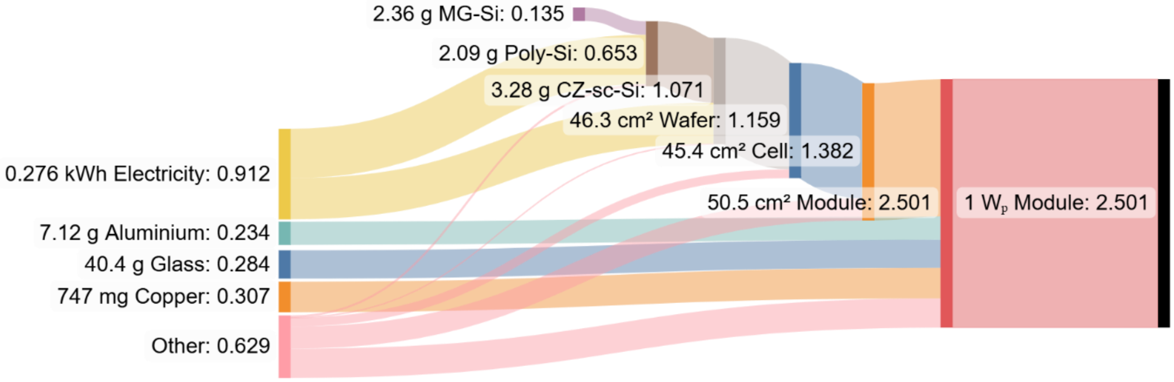

***Fig. S5** Sankey diagram of the supply chain for the production of 1 Watt-peak (W_p) of PERC solar panel capacity. Values behind each colon represent the terrestrial acidification footprint of that product in g SO_2-eq/W_p. MG-Si: metallurgical grade silicon; poly-Si: poly-silicon; Cz-sc-Si: Czochralski single-crystalline silicon.*

Results for the OAT sensitivity analyses of the ReCiPe 2016 (H) acidification midpoint impact category are provided in Tab. S5. The values in rows labelled F, B, and F+B correspond with impact reductions due to developments in the foreground, background, or both, respectively. The percentage change in acidification footprint is calculated between 2020 and 2050 ($\Delta_{2020\rightarrow2050}$). The final eleven rows represent OAT sensitivity values when excluding a single learning curve in the foreground system, where the percentage change in acidification footprint is calculated between "F+B" and "F+B, excl. [a single learning curve (e.g. efficiency)]". From Tab. S5 it becomes apparent that exclusion of learning in panel efficiency results in the largest increase in acidification footprint and, therefore, the acidification footprint is most sensitive to this process parameter.

Tab. S5. *One-at-a-time sensitivity analyses for the ReCiPe 2016 (H) acidification midpoint impact category showing how sensitive the percentage impact reductions between 2020 and 2050 are to modelled developments in only the foreground system (F), only the background system (B), or both (F+B), and to exclusion of individual learning curves for the foreground system.*

	**2020**
**Baseline**	**kg SO_2-eq.**
	0.001371

	**2050 SSP2-base**		**2050 SSP2-RCP1.9**	
	**kg SO_2-eq.**	**$\Delta_{2020\rightarrow2050}$**	**kg SO_2-eq.**	**$\Delta_{2020\rightarrow2050}$**
**F**	0.001092	−20%	0.001025	−25%
**B**	0.001246	−9%	0.000863	−37%
**F+B**	0.001006	−27%	0.000707	−48%
	**kg SO_2-eq.**	**$\Delta_{FB\rightarrow FB\ excl.}$**	**kg SO_2-eq.**	**$\Delta_{2020\rightarrow2050}$**
**F+B, excluding efficiency**	0.001081	7.5%	0.000777	10.0%
**F+B, excluding thickness wafer**	0.001031	2.5%	0.000718	1.6%
**F+B, excluding thickness kerf**	0.001032	2.7%	0.000718	1.6%
**F+B, excluding thickness glass**	0.001026	2.0%	0.000731	3.5%
**F+B, excluding mass frame**	0.001009	0.3%	0.000711	0.5%
**F+B, excluding mass silver**	0.001019	1.3%	0.000721	1.9%
**F+B, excluding power consumption MG-Si**	0.001007	0.2%	0.000707	0.1%
**F+B, excluding power consumption poly-Si**	0.001017	1.1%	0.000709	0.3%
**F+B, excluding power consumption Cz-sc-Si**	0.001023	1.8%	0.000710	0.5%
**F+B, excluding power consumption cell production**	0.001028	2.2%	0.000712	0.7%
**F+B, excluding power consumption module production**	0.001009	0.3%	0.000708	0.1%

Fig. S6 displays the Spearman's rank correlation coefficients between the acidification footprint of PERC panel production and each of the eleven parameters for which process-specific learning curves were created. The higher the Spearman's rank correlation coefficient of a process parameter, the more it contributes to the uncertainty in the acidification footprint of PERC panel production. Module efficiency has a negative correlation coefficient, meaning that higher module efficiency correlate with lower acidification footprints. The other process parameters all have positive correlation coefficients, meaning that an increase in these parameter values result in a higher acidification footprints.

In the SSP2-base scenarios, the uncertainty in the acidification footprints is most affected by uncertainty in power consumption in module and Czochralski single-crystal silicon production. Sulfur in fossil fuels is released as sulfur dioxide during combustion of said fossil fuels and nitrogen oxides are simultaneously formed. Sulfur dioxide and nitrogen oxides both contribute to acidification. However, in a decarbonized economy (i.e. SSP2-RCP19), fewer fossil fuels are used in energy generation and, therefore, the acidification footprint of the consumed electricity diminishes. This in turn makes the acidification footprint of producing a PERC panel less sensitive to uncertainty in process-specific learning curves for electricity consumption. As a result, the next two main contributors, glass and the frame (see Fig. S5) become the largest contributors to uncertainty in the acidification footprint of producing a PERC panel. The uncertainty in projected acidification footprints from PERC panel production might then be reduced by directing research efforts towards reducing uncertainty for the process-specific learning curve of the thickness of the glass and the mass of the frame, e.g. by collecting more datapoints or by further disaggregating this learning curve into multiple process-specific learning curves for producing a frame.

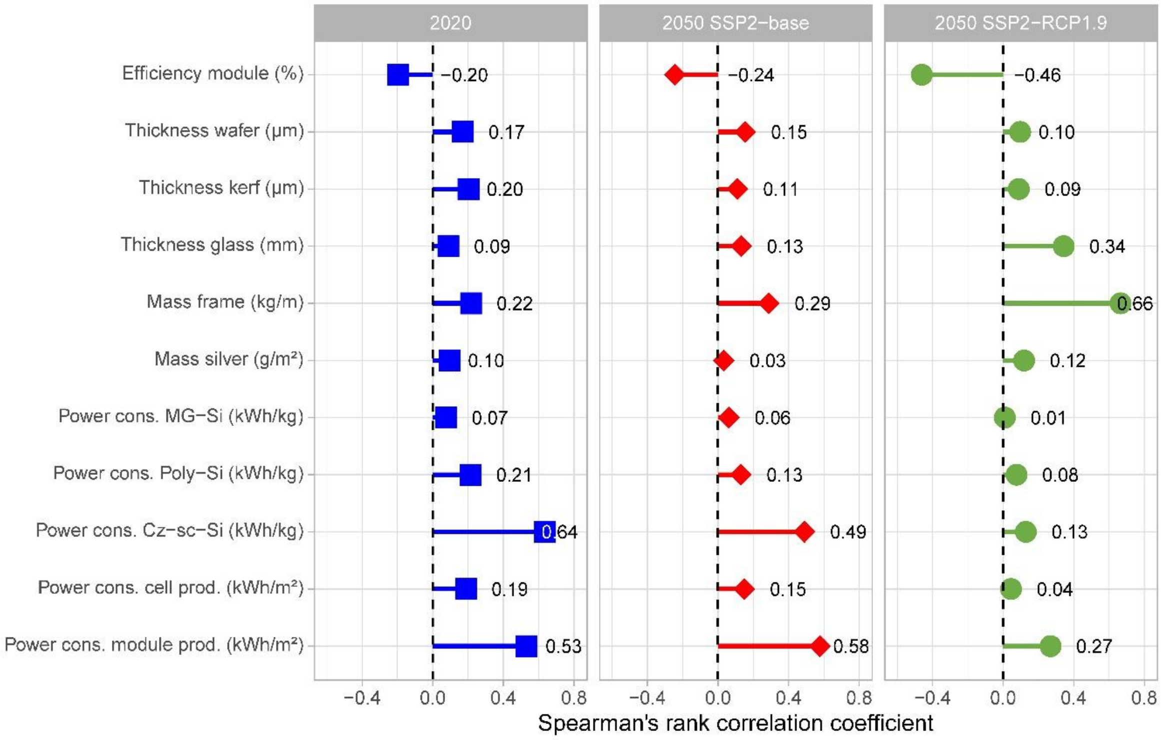

Fig. S6. *Uncertainty analyses showing the Spearman's rank correlation coefficients relating the 1,000 terrestrial acidification footprints obtained for each scenario against the eleven process parameters adapted in the foreground system using learning curves. F: foreground; B: background; MG-Si: metallurgical grade silicon; poly-Si: poly-silicon; Cz-sc-Si: Czochralski single-crystalline silicon; cons.: consumption; prod.: production.*

2.1.4. Human Toxicity: Non-Carcinogenic

The output of the *print_recursive_calculation* is as follows:

```
Fraction of score | Absolute score | Amount | Activity
0001 | 1.044 |     1 | 'glass-backsheet PERC module production' (watt peak, CN, None)
*0001 | 1.044 | 0.005052 | 'glass-backsheet PERC module production' (square meter, CN, None)
**0.418 | 0.4363 | 0.004537 | 'PERC cell production, mono Si M6 wafer' (square meter, CN, None)
***0.168 | 0.175 | 0.004627 | '170 µm mono M6 bricking and wafer production, photovoltaic' (square m
****0.136 | 0.1419 | 0.003276 | 'p-type silicon production, single crystal, Czochralski process' (kilo
*****0.0823 | 0.08589 | 0.002093 | 'silicon production, solar grade, modified Siemens process' (kilogram,
******0.0631 | 0.06583 | 0.1507 | 'market group for electricity, medium voltage' (kilowatt hour, CN, Non
*******0.0558 | 0.05825 | 0.1247 | 'market group for electricity, medium voltage' (kilowatt hour, CN-SGCC
*****0.0526 | 0.05494 | 0.1258 | 'market group for electricity, medium voltage' (kilowatt hour, CN, Non
******0.0466 | 0.04862 | 0.104 | 'market group for electricity, medium voltage' (kilowatt hour, CN-SGCC
***0.0321 | 0.03347 | 4.627e-06 | 'metallization paste production, back side' (kilogram, CN, None)
****0.0321 | 0.03346 | 2.314e-06 | 'market for silver' (kilogram, GLO, None)
*****0.0268 | 0.02798 | 1.74e-06 | 'silver-gold mine operation with refinery' (kilogram, RoW, None)
***0.183 | 0.1915 | 1.579e-05 | 'market for metallization paste, front side' (kilogram, RER, None)
****0.183 | 0.1915 | 1.579e-05 | 'metallization paste production, front side' (kilogram, RER, None)
*****0.183 | 0.1914 | 1.323e-05 | 'market for silver' (kilogram, GLO, None)
******0.153 |  0.16 | 9.953e-06 | 'silver-gold mine operation with refinery' (kilogram, RoW, None)
*******0.0317 | 0.03312 | -0.01394 | 'market for sulfidic tailings, from silver mine operation' (kilogram,
*******0.102 | 0.1067 | -0.04498 | 'market for sulfidic tailings, from silver mine operation' (kilogram,
**0.0747 | 0.07795 | 0.007125 | 'aluminium alloy production, AlMg3' (kilogram, CN, None)
***0.0746 | 0.07787 | 0.007125 | 'aluminium alloy production, AlMg3' (kilogram, RER, None)
****0.0599 | 0.06249 | 0.006875 | 'market for aluminium, cast alloy' (kilogram, GLO, None)
*****0.0282 | 0.02947 | 0.001797 | 'aluminium ingot, primary, to aluminium, cast alloy market' (kilogram,
**0.387 | 0.4035 | 0.0007477 | 'market for copper, cathode' (kilogram, GLO, None)
***0.116 | 0.121 | 0.0001275 | 'copper production, cathode, solvent extraction and electrowinning pro
****0.0295 | 0.03075 | -0.0181 | 'market for sulfidic tailings, from copper mine operation' (kilogram,
*****-0.0295 | -0.03075 | 0.0181 | 'treatment of sulfidic tailings, from copper mine operation, tailings
***0.259 | 0.2702 | 0.0004523 | 'electrorefining of copper, anode' (kilogram, GLO, None)
****0.257 | 0.2687 | 0.000449 | 'market for copper, anode' (kilogram, GLO, None)
*****0.0956 | 0.09981 | 0.0001675 | 'smelting of copper concentrate, sulfide ore' (kilogram, RoW, None)
******0.0461 | 0.04811 | 0.0005578 | 'market for copper concentrate, sulfide ore' (kilogram, GLO, None)
*****0.109 | 0.1142 | 0.0001708 | 'smelting of copper concentrate, sulfide ore' (kilogram, CN, None)
******0.0547 | 0.05709 | 0.0006618 | 'market for copper concentrate, sulfide ore' (kilogram, GLO, None)
```

The Sankey diagram for this output is provided in Fig. S7.

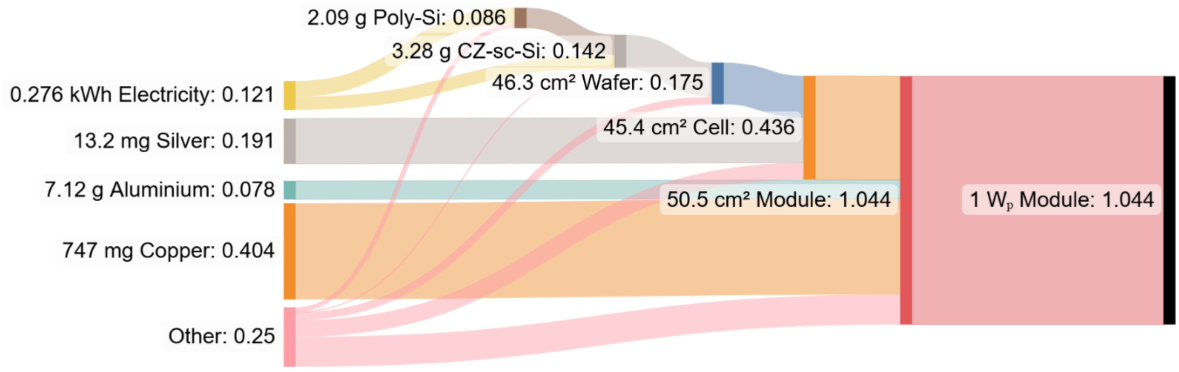

Fig. S7. *Sankey diagram of the supply chain for the production of 1 Watt-peak (W_p) of PERC solar panel capacity. Values behind each colon represent the non-carcinogenic human toxicity footprint of that product in kg 1,4-DCB-Eq./W_p. poly-Si: poly-silicon; Cz-sc-Si: Czochralski single-crystalline silicon.*

Results for the OAT sensitivity analyses of the ReCiPe 2016 (H) non-carcinogenic human toxicity midpoint impact category are provided in Tab. S6. The values in rows labelled F, B, and F+B correspond with impact reductions due to developments in the foreground, background, or both, respectively. The percentage change in non-carcinogenic human toxicity footprint is calculated between 2020 and 2050 ($\Delta_{2020\rightarrow2050}$). The final eleven rows represent OAT sensitivity values when excluding a single learning curve in the foreground system, where the percentage change in non-carcinogenic human toxicity footprint is calculated between "F+B" and "F+B, excl. [a single learning curve (e.g. efficiency)]". From Tab. S6 it becomes apparent that exclusion of learning in silver consumption results in the largest increase in the non-carcinogenic human toxicity footprint and, therefore, the non-carcinogenic human toxicity footprint is most sensitive to this process parameter.

Tab. S6. *One-at-a-time sensitivity analyses for the ReCiPe 2016 (H) human non-carcinogenic toxicity midpoint impact category showing how sensitive the percentage impact reductions between 2020 and 2050 are to modelled developments in only the foreground system (F), only the background system (B), or both (F+B), and to exclusion of individual learning curves for the foreground system.*

	**2020**			
**Baseline**	**kg 1,4-DCB-eq.**			
	0.823617			
	**2050 SSP2-base**		**2050 SSP2-RCP1.9**	
	**kg 1,4-DCB-eq.**	**$\Delta_{2020\rightarrow2050}$**	**kg 1,4-DCB-eq.**	**$\Delta_{2020\rightarrow2050}$**
**F**	0.654071	−21%	0.615019	−25%
**B**	0.815604	−1%	0.706912	−14%
**F+B**	0.649707	−21%	0.542552	−34%
	**kg 1,4-DCB-eq.**	**$\Delta_{FB\rightarrow FB\ excl.}$**	**kg 1,4-DCB-eq.**	**$\Delta_{2020\rightarrow2050}$**
**F+B, excluding efficiency**	0.698623	7.5%	0.596602	10.0%
**F+B, excluding thickness wafer**	0.656859	1.1%	0.545624	0.6%
**F+B, excluding thickness kerf**	0.657170	1.1%	0.545664	0.6%
**F+B, excluding thickness glass**	0.651158	0.2%	0.544141	0.3%
**F+B, excluding mass frame**	0.651143	0.2%	0.544229	0.3%
**F+B, excluding mass silver**	0.720530	10.9%	0.626067	15.4%
**F+B, excluding power consumption MG-Si**	0.650270	0.1%	0.542746	0.0%
**F+B, excluding power consumption poly-Si**	0.653253	0.5%	0.543778	0.2%
**F+B, excluding power consumption Cz-sc-Si**	0.655365	0.9%	0.544431	0.3%
**F+B, excluding power consumption cell production**	0.656842	1.1%	0.545079	0.5%
**F+B, excluding power consumption module production**	0.650802	0.2%	0.542951	0.1%

Fig. S8 displays the Spearman's rank correlation coefficients between the non-carcinogenic human toxicity footprint of PERC panel production and each of the eleven parameters for which process-specific learning curves were created. The higher the Spearman's rank correlation coefficient of a process parameter, the more it contributes to the uncertainty in the non-carcinogenic human toxicity footprint of PERC panel production. Module efficiency has a negative correlation coefficient, meaning that higher module efficiency correlate with lower non-carcinogenic human toxicity footprints. The other process parameters all have positive or near-zero correlation coefficients, meaning that an increase in these parameter values result in a higher or comparable non-carcinogenic human toxicity footprint, respectively.

In the SSP2-base scenarios, the uncertainty in the non-carcinogenic human toxicity footprint is most affected by uncertainty in the main contributors (see Fig. S7), which are silver consumption in wafer production, aluminium consumption in frame production, and power consumption in Czochralski single-crystal silicon production. Furthermore, power consumption in module production is a major contributor to uncertainty as it has the largest uncertainty of any process-specific learning curves (see Fig. 2 in the main text). Release of heavy metals during silver, aluminium and fossil fuel mining, and release of sulphur dioxide, nitrogen oxides and volatile organic compounds in fossil fuel combustion contribute to non-carcinogenic human toxicity. However, in a decarbonized economy (i.e. SSP2-RCP19), the non-carcinogenic human toxicity footprint of the consumed electricity diminishes as fewer fuels are used. Therefore, the non-carcinogenic human toxicity footprint of producing a PERC panel become less sensitive to uncertainty in process-specific learning curves for electricity consumption. As a result, the mass of silver and the frame and the module efficiency become the largest contributors to uncertainty in the non-carcinogenic human toxicity footprint of producing a PERC panel. This uncertainty might then be reduced by directing research efforts towards reducing uncertainty for the process-specific learning curve of the mass of silver and the frame and the module efficiency, e.g. by collecting more datapoints or by further disaggregating the learning curve of the mass of silver and the frame into multiple process-specific learning curves for producing metallization paste or a frame.

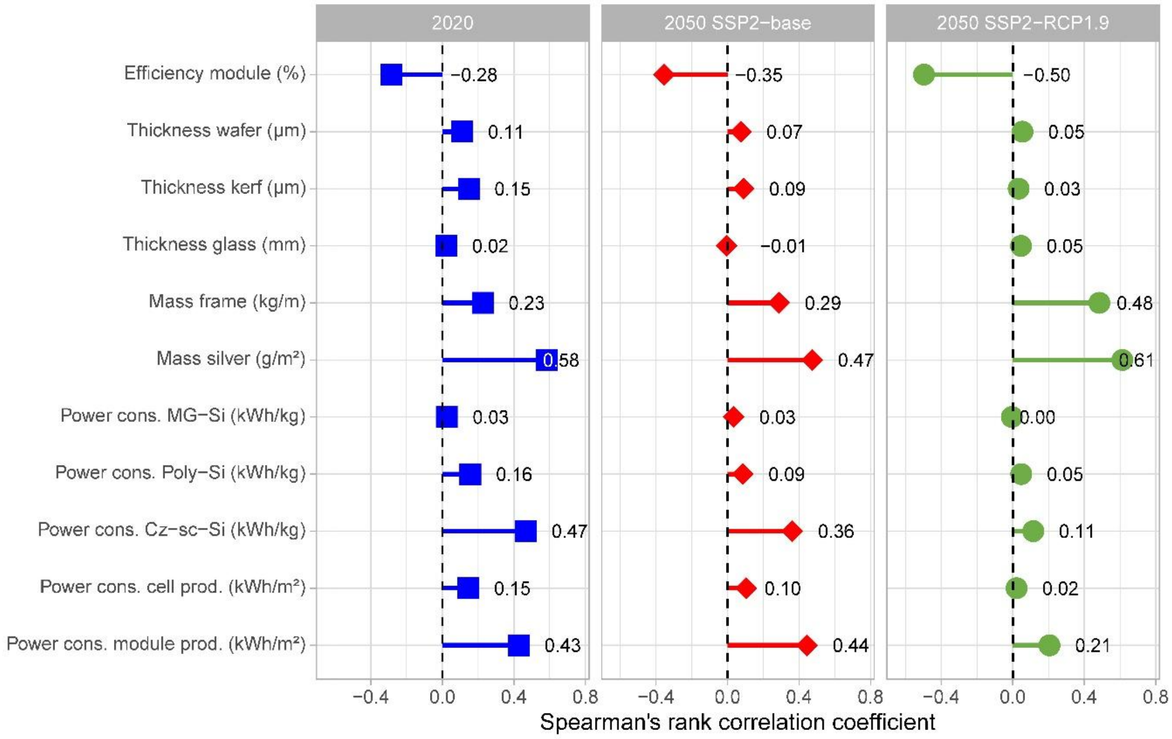

Fig. S8. *Uncertainty analyses showing the Spearman's rank correlation coefficients relating the 1,000 non-carcinogenic human toxicity footprints obtained for each scenario against the eleven process parameters adapted in the foreground system using learning curves. F: foreground; B: background; MG-Si: metallurgical grade silicon; poly-Si: poly-silicon; Cz-sc-Si: Czochralski single-crystalline silicon; cons.: consumption; prod.: production.*

2.1.5. Particulate Matter Formation

The output of the *print_recursive_calculation* is as follows:

```
Fraction of score | Absolute score | Amount | Activity
0001 | 0.001071 |     1 | 'glass-backsheet PERC module production' (watt peak, CN, None)
*0001 | 0.001071 | 0.005052 | 'glass-backsheet PERC module production' (square meter, CN, None)
**0.584 | 0.0006257 | 0.004537 | 'PERC cell production, mono Si M6 wafer' (square meter, CN, None)
***0.477 | 0.0005115 | 0.004627 | '170 µm mono M6 bricking and wafer production, photovoltaic' (square m
****0.445 | 0.0004766 | 0.003276 | 'p-type silicon production, single crystal, Czochralski process' (kilo
*****0.269 | 0.0002884 | 0.002093 | 'silicon production, solar grade, modified Siemens process' (kilogram,
******0.0542 | 5.805e-05 | 0.002365 | 'silicon production, metallurgical grade' (kilogram, CN, None)
*******0.0357 | 3.829e-05 | 0.02602 | 'market group for electricity, medium voltage' (kilowatt hour, CN, Non
******0.207 | 0.0002218 | 0.1507 | 'market group for electricity, medium voltage' (kilowatt hour, CN, Non
*******0.183 | 0.000196 | 0.1247 | 'market group for electricity, medium voltage' (kilowatt hour, CN-SGCC
*****0.173 | 0.0001851 | 0.1258 | 'market group for electricity, medium voltage' (kilowatt hour, CN, Non
******0.153 | 0.0001636 | 0.104 | 'market group for electricity, medium voltage' (kilowatt hour, CN-SGCC
*******0.0424 | 4.547e-05 | 0.02382 | 'market for electricity, medium voltage' (kilowatt hour, CN-NCGC, None
*******0.0334 | 3.582e-05 | 0.02828 | 'market for electricity, medium voltage' (kilowatt hour, CN-ECGC, None
*******0.0315 | 3.378e-05 | 0.01542 | 'market for electricity, medium voltage' (kilowatt hour, CN-NECG, None
***0.0376 | 4.026e-05 | 0.02736 | 'market group for electricity, medium voltage' (kilowatt hour, CN, Non
****0.0332 | 3.557e-05 | 0.02263 | 'market group for electricity, medium voltage' (kilowatt hour, CN-SGCC
**0.116 | 0.0001238 | 0.007125 | 'aluminium alloy production, AlMg3' (kilogram, CN, None)
***0.105 | 0.0001125 | 0.007125 | 'aluminium alloy production, AlMg3' (kilogram, RER, None)
****0.0713 | 7.633e-05 | 0.006875 | 'market for aluminium, cast alloy' (kilogram, GLO, None)
*****0.0658 | 7.051e-05 | 0.001797 | 'aluminium ingot, primary, to aluminium, cast alloy market' (kilogram,
******0.0575 | 6.158e-05 | 0.001396 | 'market for aluminium, primary, ingot' (kilogram, RoW, None)
*******0.0387 | 4.146e-05 | 0.0009819 | 'aluminium production, primary, ingot' (kilogram, CN, None)
****0.0253 | 2.709e-05 | 0.0002173 | 'market for magnesium' (kilogram, GLO, None)
*****0.0252 | 2.695e-05 | 0.00018 | 'magnesium production, pidgeon process' (kilogram, CN, None)
**0.0894 | 9.578e-05 | 0.04042 | 'flat glass production, uncoated' (kilogram, CN, None)
***0.0853 | 9.134e-05 | 0.04042 | 'flat glass production, uncoated' (kilogram, RER, None)
**0.0921 | 9.863e-05 | 0.0007477 | 'market for copper, cathode' (kilogram, GLO, None)
***0.0775 | 8.302e-05 | 0.0004523 | 'electrorefining of copper, anode' (kilogram, GLO, None)
****0.0752 | 8.054e-05 | 0.000449 | 'market for copper, anode' (kilogram, GLO, None)
*****0.0256 | 2.747e-05 | 0.0001675 | 'smelting of copper concentrate, sulfide ore' (kilogram, RoW, None)
*****0.0289 | 3.097e-05 | 0.0001708 | 'smelting of copper concentrate, sulfide ore' (kilogram, CN, None)
```

The Sankey diagram for this output is provided in Fig. S9.

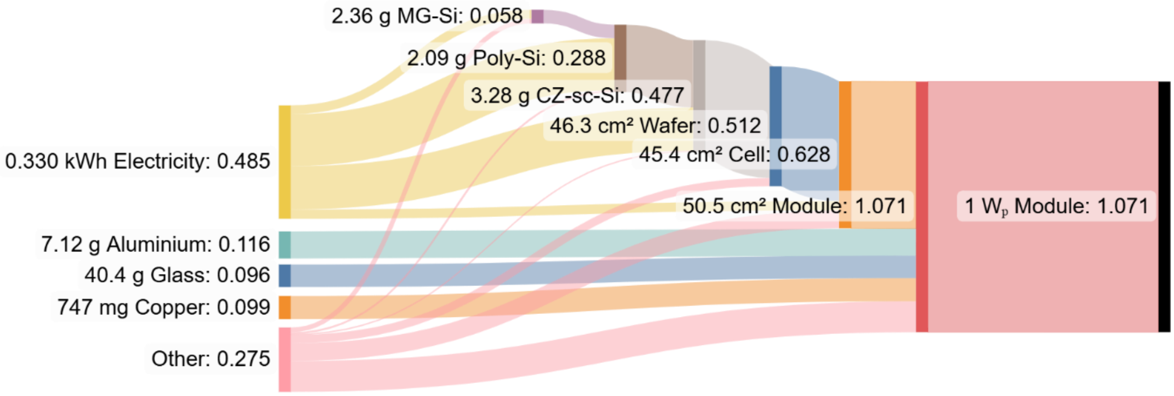

Fig. S9. *Sankey diagram of the supply chain for the production of 1 Watt-peak (W_p) of PERC solar panel capacity. Values behind each colon represent the particulate matter formation footprint of that product in g PM2.5-Eq./W_p. MG-Si: metallurgical grade silicon; poly-Si: poly-silicon; Cz-sc-Si: Czochralski single-crystalline silicon.*

Results for the OAT sensitivity analyses of the ReCiPe 2016 (H) particulate matter formation midpoint impact category are provided in Tab. S7. The values in rows labelled F, B, and F+B correspond with impact reductions due to developments in the foreground, background, or both, respectively. The percentage change in particulate matter formation footprint is calculated between 2020 and 2050 ($\Delta_{2020→2050}$). The final eleven rows represent OAT sensitivity values when excluding a single learning curve in the foreground system, where the percentage change in particulate matter formation footprint is calculated between “F+B” and “F+B, excl. [a single learning curve (e.g. efficiency)]”. From Tab. S7 it becomes apparent that exclusion of learning in panel efficiency results in the largest increase in particulate matter formation footprint and, therefore, the particulate matter formation footprint is most sensitive to this process parameter.

Tab. S7. *One-at-a-time sensitivity analyses for the ReCiPe 2016 (H) particulate matter formation midpoint impact category showing how sensitive the percentage impact reductions between 2020 and 2050 are to modelled developments in only the foreground system (F), only the background system (B), or both (F+B), and to exclusion of individual learning curves for the foreground system.*

	**2020**
**Baseline**	**kg PM2.5-eq.**
	0.000550551

	**2050 SSP2-base**		**2050 SSP2-RCP1.9**	
	**kg PM2.5-eq.**	**$\Delta_{2020→2050}$**	**kg PM2.5-eq.**	**$\Delta_{2020→2050}$**
**F**	0.00043	−22%	0.000401	−27%
**B**	0.00047	−15%	0.000311	−43%
**F+B**	0.000375	−32%	0.000254	−54%
	**kg PM2.5-eq.**	**$\Delta_{FB→FB\ excl.}$**	**kg PM2.5-eq.**	**$\Delta_{2020→2050}$**
**F+B, excluding efficiency**	0.000403	7.5%	0.000280	10.0%
**F+B, excluding thickness wafer**	0.000386	2.9%	0.000259	1.8%
**F+B, excluding thickness kerf**	0.000386	3.0%	0.000259	1.8%
**F+B, excluding thickness glass**	0.000382	1.7%	0.000262	3.1%
**F+B, excluding mass frame**	0.000377	0.4%	0.000256	0.7%
**F+B, excluding mass silver**	0.000380	1.3%	0.000259	1.9%
**F+B, excluding power consumption MG-Si**	0.000376	0.2%	0.000254	0.1%
**F+B, excluding power consumption poly-Si**	0.000380	1.3%	0.000255	0.3%
**F+B, excluding power consumption Cz-sc-Si**	0.000383	2.0%	0.000256	0.5%
**F+B, excluding power consumption cell production**	0.000385	2.5%	0.000256	0.7%
**F+B, excluding power consumption module production**	0.000377	0.4%	0.000255	0.1%

Fig. S10 displays the Spearman's rank correlation coefficients between the particulate matter formation footprint for PERC panel production and each of the eleven parameters for which process-specific learning curves were created. The higher the Spearman's rank correlation coefficient of a process parameter, the more it contributes to the uncertainty in the particulate matter formation footprint of PERC panel production. Module efficiency has a negative correlation coefficient, meaning that higher module efficiency correlate with lower particulate matter formation footprints. The other process parameters all have positive correlation coefficients, meaning that an increase in these parameter values result in a higher particulate matter formation footprints.

In the SSP2-base scenarios, the uncertainty in the particulate matter formation footprints is most affected by uncertainty in power consumption in module and Czochralski single-crystal silicon production. Particulate matter is mostly formed in electricity generation during combustion of fossil fuels. However, in a decarbonized economy (i.e. SSP2-RCP19), fewer fossil fuels are used in electricity generation and, therefore, the particulate matter formation footprint of the consumed electricity diminishes. The particulate matter formation footprint of producing a PERC panel thus becomes less sensitive to uncertainty in process-specific learning curves for electricity consumption. As a result, the next main contributors, the frame (see Fig. S9) becomes the largest contributors to uncertainty in the particulate matter formation footprint of producing a PERC panel. The uncertainty in projected particulate matter formation footprint of PERC panel production might then be reduced by directing research efforts towards reducing uncertainty for the process-specific learning curve of the mass of the frame, e.g. by collecting more datapoints or by further disaggregating this learning curve into multiple process-specific learning curves for producing a frame.

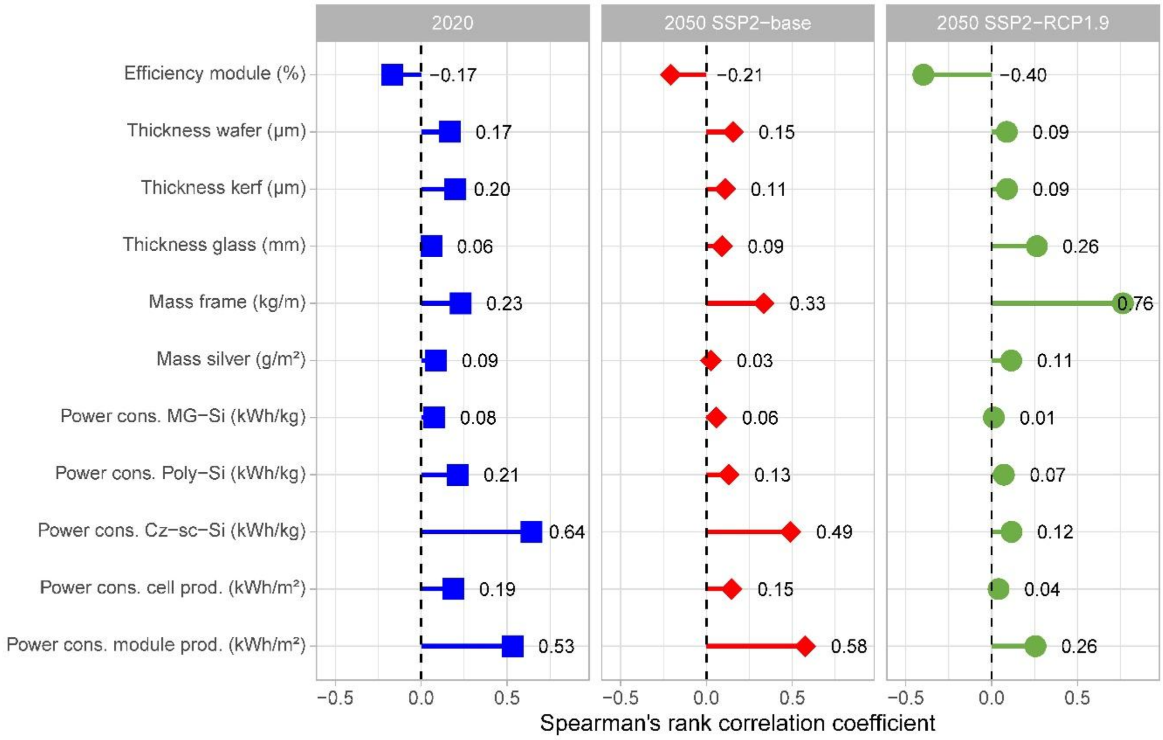

Fig. S10. *Uncertainty analyses showing the Spearman's rank correlation coefficients relating the 1,000 particulate matter formation footprints obtained for each scenario against the eleven process parameters adapted in the foreground system using learning curves. F: foreground; B: background; MG-Si: metallurgical grade silicon; poly-Si: poly-silicon; Cz-sc-Si: Czochralski single-crystalline silicon; cons.: consumption; prod.: production.*

2.1.6. Energy Resources: Non-renewable, Fossil

The output of the *print_recursive_calculation* is as follows:

```
Fraction of score | Absolute score | Amount | Activity
0001 | 0.1345 |     1 | 'glass-backsheet PERC module production' (watt peak, CN, None)
*0001 | 0.1345 | 0.005052 | 'glass-backsheet PERC module production' (square meter, CN, None)
**0.63 | 0.08474 | 0.004537 | 'PERC cell production, mono Si M6 wafer' (square meter, CN, None)
***0.53 | 0.0713 | 0.004627 | '170 µm mono M6 bricking and wafer production, photovoltaic' (square m
****00.5 | 0.06718 | 0.003276 | 'p-type silicon production, single crystal, Czochralski process' (kilo
*****0.313 | 0.0421 | 0.002093 | 'silicon production, solar grade, modified Siemens process' (kilogram,
******0.0559 | 0.007515 | 0.002365 | 'silicon production, metallurgical grade' (kilogram, CN, None)
*******0.0383 | 0.005149 | 0.02602 | 'market group for electricity, medium voltage' (kilowatt hour, CN, Non
******0.0283 | 0.003809 | 0.1465 | 'heat production, natural gas, at industrial furnace >100kW' (megajoul
******0.222 | 0.02982 | 0.1507 | 'market group for electricity, medium voltage' (kilowatt hour, CN, Non
*******0.196 | 0.02629 | 0.1247 | 'market group for electricity, medium voltage' (kilowatt hour, CN-SGCC
*******0.0263 | 0.003536 | 0.02605 | 'market for electricity, medium voltage' (kilowatt hour, CN-CSG, None)
*****0.185 | 0.02489 | 0.1258 | 'market group for electricity, medium voltage' (kilowatt hour, CN, Non
******0.163 | 0.02194 | 0.104 | 'market group for electricity, medium voltage' (kilowatt hour, CN-SGCC
*******0.044 | 0.005921 | 0.02382 | 'market for electricity, medium voltage' (kilowatt hour, CN-NCGC, None
*******0.0384 | 0.005167 | 0.02828 | 'market for electricity, medium voltage' (kilowatt hour, CN-ECGC, None
*******0.0337 | 0.00453 | 0.01542 | 'market for electricity, medium voltage' (kilowatt hour, CN-NECG, None
***0.0403 | 0.005414 | 0.02736 | 'market group for electricity, medium voltage' (kilowatt hour, CN, Non
****0.0355 | 0.004772 | 0.02263 | 'market group for electricity, medium voltage' (kilowatt hour, CN-SGCC
**0.0947 | 0.01274 | 0.007125 | 'aluminium alloy production, AlMg3' (kilogram, CN, None)
***0.0862 | 0.01159 | 0.007125 | 'aluminium alloy production, AlMg3' (kilogram, RER, None)
****0.0633 | 0.008507 | 0.006875 | 'market for aluminium, cast alloy' (kilogram, GLO, None)
*****0.0558 | 0.007504 | 0.001797 | 'aluminium ingot, primary, to aluminium, cast alloy market' (kilogram,
******0.0487 | 0.006546 | 0.001396 | 'market for aluminium, primary, ingot' (kilogram, RoW, None)
*******0.034 | 0.004568 | 0.0009819 | 'aluminium production, primary, ingot' (kilogram, CN, None)
**0.0753 | 0.01013 | 0.04042 | 'flat glass production, uncoated' (kilogram, CN, None)
***0.072 | 0.009679 | 0.04042 | 'flat glass production, uncoated' (kilogram, RER, None)
****0.0337 | 0.004531 | 0.004692 | 'market group for natural gas, high pressure' (cubic meter, Europe wit
**0.0549 | 0.007385 | 0.004006 | 'market for ethylvinylacetate, foil' (kilogram, GLO, None)
***0.0371 | 0.004982 | 0.002682 | 'ethylvinylacetate production, foil' (kilogram, RoW, None)
****0.0342 | 0.004599 | 0.002735 | 'market for ethylene vinyl acetate copolymer' (kilogram, RoW, None)
*****0.0336 | 0.004524 | 0.002735 | 'ethylene vinyl acetate copolymer production' (kilogram, RoW, None)
```

The Sankey diagram for this output is provided in Fig. S11.

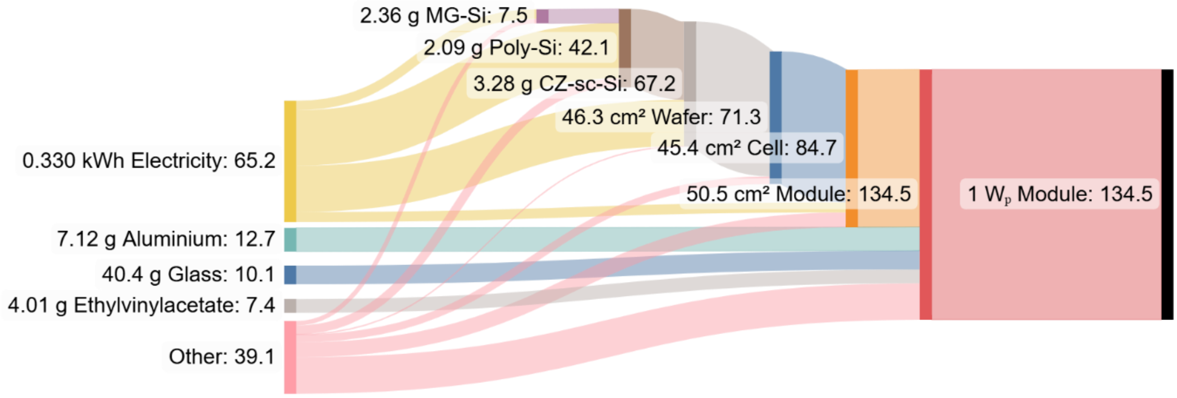

Fig. S11. *Sankey diagram of the supply chain for the production of 1 Watt-peak (W_p) of PERC solar panel capacity. Values behind each colon represent the non-renewable fossil energy resource footprint of that product in g oil-Eq./W_p. MG-Si: metallurgical grade silicon; poly-Si: poly-silicon; Cz-sc-Si: Czochralski single-crystalline silicon.*

Results for the OAT sensitivity analyses of the ReCiPe 2016 (H) non-renewable fossil energy resources midpoint impact category are provided in Tab. S8. The values in rows labelled F, B, and F+B correspond with impact reductions due to developments in the foreground, background, or both, respectively. The percentage change in non-renewable fossil energy resources footprint is calculated between 2020 and 2050 ($\Delta_{2020\rightarrow2050}$). The final eleven rows represent OAT sensitivity values when excluding a single learning curve in the foreground system, where the percentage change in non-renewable fossil energy resources footprint is calculated between "F+B" and "F+B, excl. [a single learning curve (e.g. efficiency)]". From Tab. S8 it becomes apparent that exclusion of learning in panel efficiency results in the largest increase in non-renewable fossil energy resources footprint and, therefore, the non-renewable fossil energy resources footprint is most sensitive to this process parameter.

Tab. S8. *One-at-a-time sensitivity analyses for the ReCiPe 2016 (H) non-renewable fossil energy resources midpoint impact category showing how sensitive the percentage impact reductions between 2020 and 2050 are to modelled developments in only the foreground system (F), only the background system (B), or both (F+B), and to exclusion of individual learning curves for the foreground system.*

**Baseline**	**2020**			
	**kg oil-eq.**			
	0.09639567			
	**2050 SSP2-base**		**2050 SSP2-RCP1.9**	
	**kg oil-eq.**	**$\Delta_{2020\rightarrow2050}$**	**kg oil-eq.**	**$\Delta_{2020\rightarrow2050}$**
**F**	0.071628	−26%	0.065913	−32%
**B**	0.088693	−8%	0.048759	−49%
**F+B**	0.066482	−31%	0.037014	−62%
	**kg oil-eq.**	**$\Delta_{FB\rightarrow FB\ excl.}$**	**kg oil-eq.**	**$\Delta_{2020\rightarrow2050}$**
**F+B, excluding efficiency**	0.071488	7.5%	0.040701	10.0%
**F+B, excluding thickness wafer**	0.069607	4.7%	0.038585	4.2%
**F+B, excluding thickness kerf**	0.069743	4.9%	0.038605	4.3%
**F+B, excluding thickness glass**	0.067245	1.1%	0.037900	2.4%
**F+B, excluding mass frame**	0.066688	0.3%	0.037225	0.6%
**F+B, excluding mass silver**	0.066991	0.8%	0.037333	0.9%
**F+B, excluding power consumption MG-Si**	0.066706	0.3%	0.037086	0.2%
**F+B, excluding power consumption poly-Si**	0.067892	2.1%	0.037472	1.2%
**F+B, excluding power consumption Cz-sc-Si**	0.068731	3.4%	0.037716	1.9%
**F+B, excluding power consumption cell production**	0.069319	4.3%	0.037958	2.6%
**F+B, excluding power consumption module production**	0.066917	0.7%	0.037163	0.4%

Fig. S12 displays the Spearman's rank correlation coefficients between the non-renewable fossil energy resources footprint for PERC panel production and each of the eleven parameters for which process-specific learning curves were created. The higher the Spearman's rank correlation coefficient of a process parameter, the more it contributes to the uncertainty in the non-renewable fossil energy resources footprint of PERC panel production. Module efficiency has a negative correlation coefficient, meaning that higher module efficiency correlate with lower non-renewable fossil energy resources footprints. The other process parameters all have positive correlation coefficients, meaning that an increase in these parameter values result in a higher non-renewable fossil energy resources footprints.

In the SSP2-base scenarios, the uncertainty in the non-renewable fossil energy resources footprint for PERC panel production is most affected by uncertainty in power consumption in module and Czochralski single-crystal silicon production. In a decarbonized economy (i.e. SSP2-RCP19), one would expect the non-renewable fossil energy resources footprint of the consumed electricity to diminish as more electricity is generated with renewable resources. As the consumption of fossil resources in electricity generation decreases, the non-renewable fossil energy resources footprint of producing a PERC panel would become less sensitive to uncertainty in process-specific learning curves for electricity consumption. A slight reduction is visible, but not as strong as e.g. in the categories of climate change and particulate matter formation. A reason for this can be found in the scenario files that are exported by premise (see the Supporting Information). Contributions of various sources of electricity are displayed in Tab. S9. In 2020, the major energy sources for electricity are pulverized coal and hydro. In the SSP2-RCP1.9 scenario for 2050, the main contributors are onshore wind, solar, hydro and natural gas with carbon capture and storage (CCS). The use of natural gas with CCS results in fewer emissions of greenhouse gasses and particulate matter, thus explaining the reduced contribution of electricity to the uncertainty in the GHG footprint and particulate matter formation footprints in the 20205 SSP2-RCP1.9 scenario. However, natural gas with CC still results in consumption of natural gas, thus contributing to the non-renewable fossil energy resources footprints. This is why electricity consumption remains a relatively large contributor to uncertainty in the non-renewable fossil energy resources footprints.

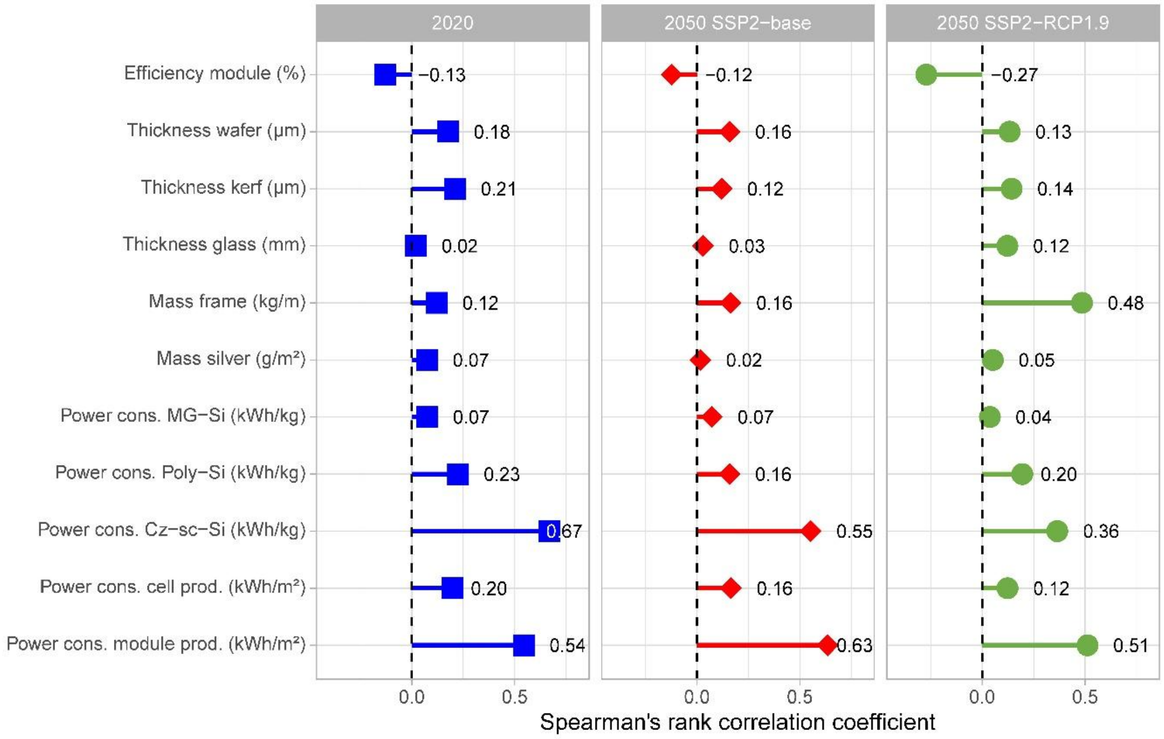

Fig. S12. *Uncertainty analyses showing the Spearman's rank correlation coefficients relating the 1,000 non-renewable fossil energy resource footprints obtained for each scenario against the eleven process parameters adapted in the foreground system using learning curves. F: foreground; B: background; MG-Si: metallurgical grade silicon; poly-Si: poly-silicon; Cz-sc-Si: Czochralski single-crystalline silicon; cons.: consumption; prod.: production.*

__Tab. S9.__ Generated volumes of electricity per technology in exajoules for China as used by premise to calculate the electricity supply mix.

	**2020**		**2050 SSP2-base**		**2050 SSP2-RCP1.9**	
	**EJ**	**%**	**EJ**	**%**	**EJ**	**%**
**Pulverized coal**	19.1	65%	28.9	49%	0	0%
**Hydro**	4.5	15%	6.7	11%	6.9	15%
**Nuclear**	1.7	6%	2.4	4%	2.7	6%
**Wind onshore**	1.7	6%	5.1	9%	17.8	39%
**Solar**	1	3%	7.4	13%	8.8	19%
**Natural gas with CCS**	0	0%	0	0%	6.0	13%
**Other**	1.2	4%	8.3	14%	3.3	7%
**Total**	**29.2**	**100%**	**58.8**	**100%**	**45.5**	**100%**

2.2. ReCiPe 2016 midpoint individualist (I)

2.2.1. Midpoint-to-Endpoint contribution analysis

A midpoint-to-endpoint contribution analysis for the production of 1 W_p of PERC solar panel was conducted using the ReCiPe 2016 endpoint life cycle impact assessment method [59, 60]. Adjustment to the characterization factors of carbon dioxide, methane, and hydrogen in the impact category of Climate Change were made in line with van der Hulst et. al 2024 [61]. Tab. S10 displays contributions of the eighteen midpoint categories to the three end-point categories for the individualist perspective. Midpoint categories contributing 15% or more to the total impact of either of the three endpoint categories are highlighted in gray and were included in further assessments at the midpoint level.

Tab. S10. *Midpoint-to-endpoint contribution analysis for producing 1 Watt-peak (W_p) of PERC solar panel capacity using the ReCiPe 2016 individualist (I) impact assessment method.*

**Endpoint**, midpoint	**Unit**	**Impact per W_p**	**Contribution**
**Ecosystem quality**	**species*yr**	**1.57E-09**	**100%**
Acidification: terrestrial, terrestrial acidification potential (TAP)	species*yr	5.30E-10	34%
Climate change: freshwater, global warming potential (GWP)	species*yr	1.03E-14	0%
Climate change: terrestrial, global warming potential (GWP)	species*yr	3.77E-10	24%
Ecotoxicity: freshwater, freshwater ecotoxicity potential (FETP)	species*yr	4.83E-11	3%
Ecotoxicity: marine, marine ecotoxicity potential (METP)	species*yr	2.03E-12	0%
Ecotoxicity: terrestrial, terrestrial ecotoxicity potential (TETP)	species*yr	1.81E-11	1%
Eutrophication: freshwater, freshwater eutrophication potential (FEP)	species*yr	1.16E-10	7%
Eutrophication: marine, marine eutrophication potential (MEP)	species*yr	7.11E-14	0%
Land use, agricultural land occupation (LOP)	species*yr	2.20E-10	14%
Photochemical oxidant formation: terrestrial ecosystems, photochemical oxidant formation potential: ecosystems (EOFP)	species*yr	2.57E-10	16%
Water use, water consumption potential (WCP)	species*yr	3.98E-15	0%
**Human Health**	**DALY**	**3.06E-07**	**100%**
Climate change, global warming potential (GWP)	DALY	5.76E-08	19%
Human toxicity: carcinogenic, human toxicity potential (HTPc)	DALY	7.68E-10	0%
Human toxicity: non-carcinogenic, human toxicity potential (HTPnc)	DALY	5.25E-09	2%
Ionising radiation, ionising radiation potential (IRP)	DALY	1.62E-10	0%
Ozone depletion, ozone depletion potential (ODPinfinite)	DALY	4.24E-11	0%
Particulate matter formation, particulate matter formation potential (PMFP)	DALY	2.20E-07	72%
Photochemical oxidant formation: human health, photochemical oxidant formation potential: humans (HOFP)	DALY	1.69E-09	1%
Water use, water consumption potential (WCP)	DALY	2.04E-08	7%
**Natural resources**	**USD2013**	**0.031**	**100%**
Energy resources: non-renewable, fossil, fossil fuel potential (FFP)	USD2013	0.027	86%
Material resources: metals/minerals, surplus ore potential (SOP)	USD2013	0.0044	14%

2.2.2. Climate change

The output of the *print_recursive_calculation* is as follows:

```
Fraction of score | Absolute score | Amount | Activity
0001 | 0.7096 |      1 | 'glass-backsheet PERC module production' (watt peak, CN, None)
*0001 | 0.7096 | 0.005052 | 'glass-backsheet PERC module production' (square meter, CN, None)
**0.702 | 0.4983 | 0.004537 | 'PERC cell production, mono Si M6 wafer' (square meter, CN, None)
***0.591 | 0.4197 | 0.004627 | '170 µm mono M6 bricking and wafer production, photovoltaic' (square m
****0.563 | 0.3996 | 0.003276 | 'p-type silicon production, single crystal, Czochralski process' (kilo
*****0.346 | 0.2459 | 0.002093 | 'silicon production, solar grade, modified Siemens process' (kilogram,
******0.0658 | 0.04668 | 0.002365 | 'silicon production, metallurgical grade' (kilogram, CN, None)
*******0.0445 | 0.03161 | 0.02602 | 'market group for electricity, medium voltage' (kilowatt hour, CN, Non
******0.258 | 0.1831 | 0.1507 | 'market group for electricity, medium voltage' (kilowatt hour, CN, Non
*******0.229 | 0.1625 | 0.1247 | 'market group for electricity, medium voltage' (kilowatt hour, CN-SGCC
*******0.029 | 0.02059 | 0.02605 | 'market for electricity, medium voltage' (kilowatt hour, CN-CSG, None)
*****0.215 | 0.1528 | 0.1258 | 'market group for electricity, medium voltage' (kilowatt hour, CN, Non
******0.191 | 0.1356 | 0.104 | 'market group for electricity, medium voltage' (kilowatt hour, CN-SGCC
*******0.0516 | 0.03661 | 0.02382 | 'market for electricity, medium voltage' (kilowatt hour, CN-NCGC, None
*******0.0432 | 0.03066 | 0.02828 | 'market for electricity, medium voltage' (kilowatt hour, CN-ECGC, None
*******0.0404 | 0.02865 | 0.01542 | 'market for electricity, medium voltage' (kilowatt hour, CN-NECG, None
*******0.0281 | 0.01994 | 0.01536 | 'market for electricity, medium voltage' (kilowatt hour, CN-NWG, None)
***0.0468 | 0.03323 | 0.02736 | 'market group for electricity, medium voltage' (kilowatt hour, CN, Non
****0.0416 | 0.02949 | 0.02263 | 'market group for electricity, medium voltage' (kilowatt hour, CN-SGCC
**0.0978 | 0.06943 | 0.007125 | 'aluminium alloy production, AlMg3' (kilogram, CN, None)
***0.0849 | 0.06025 | 0.007125 | 'aluminium alloy production, AlMg3' (kilogram, RER, None)
****0.0651 | 0.04619 | 0.006875 | 'market for aluminium, cast alloy' (kilogram, GLO, None)
*****0.0584 | 0.04147 | 0.001797 | 'aluminium ingot, primary, to aluminium, cast alloy market' (kilogram,
******0.052 | 0.03689 | 0.001396 | 'market for aluminium, primary, ingot' (kilogram, RoW, None)
*******0.0397 | 0.0282 | 0.0009819 | 'aluminium production, primary, ingot' (kilogram, CN, None)
**0.071 | 0.05036 | 0.04042 | 'flat glass production, uncoated' (kilogram, CN, None)
***0.0658 | 0.04672 | 0.04042 | 'flat glass production, uncoated' (kilogram, RER, None)
**0.0287 | 0.02037 | 0.01677 | 'market group for electricity, medium voltage' (kilowatt hour, CN, Non
***0.0255 | 0.01808 | 0.01387 | 'market group for electricity, medium voltage' (kilowatt hour, CN-SGCC
```

The Sankey diagram for this output is provided Fig. S13.

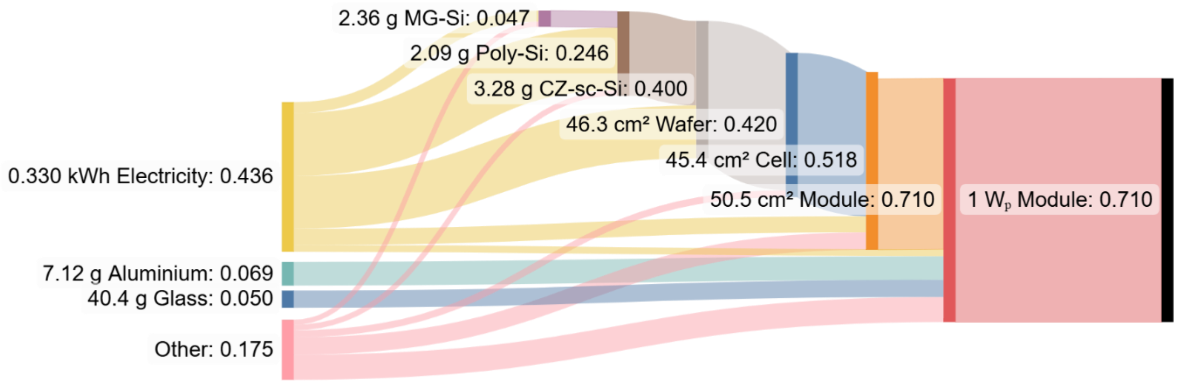

Fig. S13. *Sankey diagram of the supply chain for the production of 1 Watt-peak (W_p) of PERC solar panel capacity. Values behind each colon represent the GHG footprint of that product in kg CO_2-eq./W_p. MG-Si: metallurgical grade silicon; poly-Si: poly-silicon; Cz-sc-Si: Czochralski single-crystalline silicon.*

Results for the OAT sensitivity analyses of the ReCiPe 2016 (I) climate change midpoint impact category are provided in Tab. S11. The values in rows labelled F, B, and F+B correspond with impact reductions due to developments in the foreground, background, or both, respectively. The percentage change in GHG footprint is calculated between 2020 and 2050 ($\Delta_{2020\rightarrow2050}$). The final eleven rows represent OAT sensitivity values when excluding a single learning curve in the foreground system, where the percentage change in GHG footprint is calculated between "F+B" and "F+B, excl. [a single learning curve (e.g. efficiency)]". From Tab. S11 it becomes apparent that exclusion of learning in panel efficiency results in the largest increase in GHG footprint and, therefore, the GHG footprint is most sensitive to this process parameter.

Tab. S11. *One-at-a-time sensitivity analyses for the ReCiPe 2016 (I) climate change midpoint impact category showing how sensitive the percentage impact reductions between 2020 and 2050 are to modelled developments in only the foreground system (F), only the background system (B), or both (F+B), and to exclusion of individual learning curves for the foreground system.*

	2020
**Baseline**	**kg CO_2-eq.**
	0.543186681

	2050 SSP2-base		2050 SSP2-RCP1.9	
	**kg CO_2-eq.**	**$\Delta_{2020\rightarrow2050}$**	**kg CO_2-eq.**	**$\Delta_{2020\rightarrow2050}$**
**F**	0.388111	−29%	0.352852	−35%
**B**	0.474863942	−13%	0.13797	−75%
**F+B**	0.342922	−37%	0.109979	−80%
	**kg CO_2-eq.**	**$\Delta_{FB\rightarrow FB\ excl.}$**	**kg CO_2-eq.**	**$\Delta_{2020\rightarrow2050}$**
**F+B, excluding efficiency**	0.36874	7.5%	0.120936	10.0%
**F+B, excluding thickness wafer**	0.361828	5.5%	0.113777	3.5%
**F+B, excluding thickness kerf**	0.362649	5.8%	0.113827	3.5%
**F+B, excluding thickness glass**	0.346635	1.1%	0.114059	3.7%
**F+B, excluding mass frame**	0.344097	0.3%	0.111064	1.0%
**F+B, excluding mass silver**	0.345324	0.7%	0.110748	0.7%
**F+B, excluding power consumption MG-Si**	0.344372	0.4%	0.11003	0.0%
**F+B, excluding power consumption poly-Si**	0.352063	2.7%	0.110299	0.3%
**F+B, excluding power consumption Cz-sc-Si**	0.357508	4.3%	0.110468	0.4%
**F+B, excluding power consumption cell production**	0.361317	5.4%	0.110637	0.6%
**F+B, excluding power consumption module production**	0.345743	0.8%	0.110083	0.1%

Fig. S14 displays the Spearman's rank correlation coefficients between the GHG footprint of PERC panel production and each of the eleven parameters for which process-specific learning curves were created. The higher the Spearman's rank correlation coefficient of a process parameter, the more it contributes to the uncertainty in the GHG footprint of PERC panel production. Module efficiency has a negative correlation coefficient, meaning that higher module efficiency correlate with lower GHG footprints. The other process parameters all have positive correlation coefficients, meaning that an increase in these parameter values result in a higher GHG footprints.

In the SSP2-base scenarios, the uncertainty in the GHG footprints is most affected by uncertainty in power consumption in module and Czochralski single-crystal silicon production. Carbon in fossil fuels is released as carbon dioxide during combustion of said fossil fuels. Furthermore, use of natural gas results in fugitive emissions of methane. Carbon dioxide and methane are a greenhouse gasses that increase the radiative forcing of the atmosphere, thus contributing to climate change. However, in a decarbonized economy (i.e. SSP2-RCP19), fewer fossil fuels are used in energy generation and, therefore, the GHG footprint of the consumed electricity diminishes. When fossil fuels are used, this is in combination with carbon capture and storage (see Tab. S9). This in turn makes the GHG footprint of producing a PERC panel less sensitive to uncertainty in process-specific learning curves for electricity consumption. As a result, the next two main contributors, glass and the frame (see Fig. S13) become the largest contributors to uncertainty in the GHG footprint of producing a PERC panel. The uncertainty in projected GHG footprints from PERC panel production might then be reduced by directing research efforts towards reducing uncertainty for the process-specific learning curve of the thickness of the glass and the mass of the frame, e.g. by collecting more datapoints or by further disaggregating this learning curve into multiple process-specific learning curves for producing a frame.

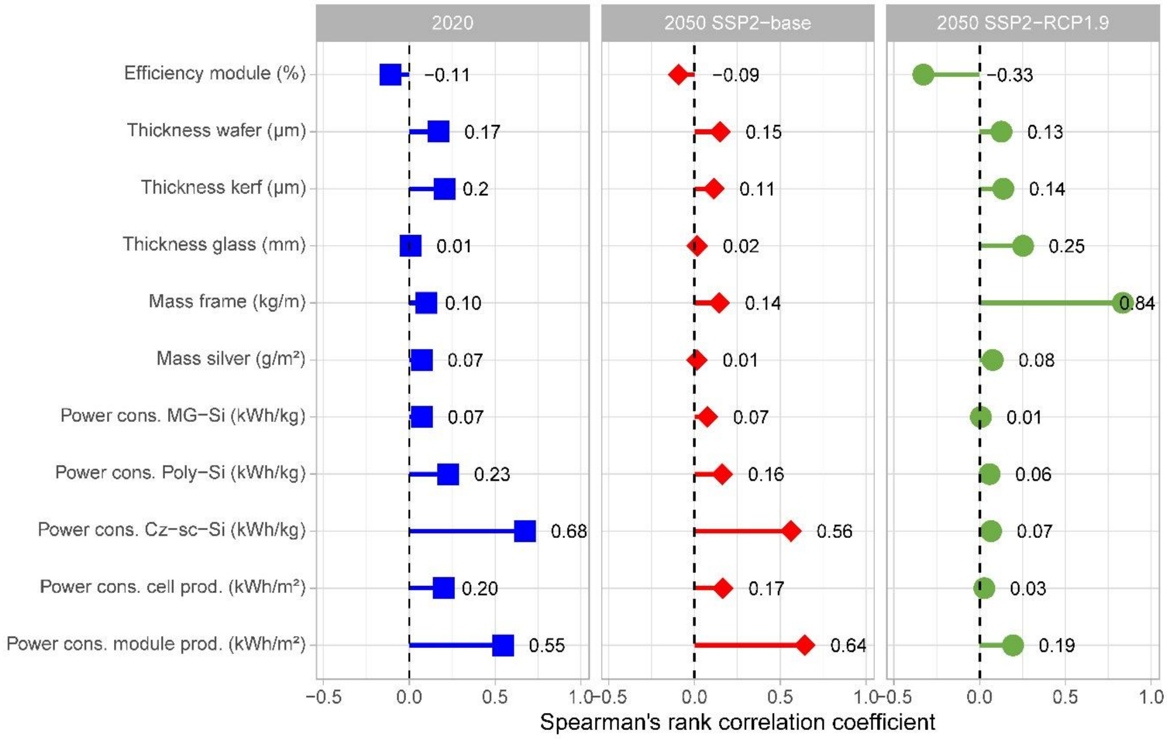

Fig. S14. *Uncertainty analyses showing the Spearman's rank correlation coefficients relating the 1,000 GHG footprints obtained for each scenario against the eleven process parameters adapted in the foreground system using learning curves. F: foreground; B: background; MG-Si: metallurgical grade silicon; poly-Si: poly-silicon; Cz-sc-Si: Czochralski single-crystalline silicon; cons.: consumption; prod.: production.*

2.2.3. Acidification

The output of the *print_recursive_calculation* is as follows:

```
Fraction of score | Absolute score | Amount | Activity
0001 | 0.002501 |     1 | 'glass-backsheet PERC module production' (watt peak, CN, None)
*0001 | 0.002501 | 0.005052 | 'glass-backsheet PERC module production' (square meter, CN, None)
**0.553 | 0.001382 | 0.004537 | 'PERC cell production, mono Si M6 wafer' (square meter, CN, None)
***0.463 | 0.001159 | 0.004627 | '170 µm mono M6 bricking and wafer production, photovoltaic' (square m
****0.428 | 0.001071 | 0.003276 | 'p-type silicon production, single crystal, Czochralski process' (kilo
*****0.261 | 0.0006531 | 0.002093 | 'silicon production, solar grade, modified Siemens process' (kilogram,
******0.054 | 0.000135 | 0.002365 | 'silicon production, metallurgical grade' (kilogram, CN, None)
*******0.0343 | 8.585e-05 | 0.02602 | 'market group for electricity, medium voltage' (kilowatt hour, CN, Non
******0.199 | 0.0004973 | 0.1507 | 'market group for electricity, medium voltage' (kilowatt hour, CN, Non
*******0.175 | 0.000437 | 0.1247 | 'market group for electricity, medium voltage' (kilowatt hour, CN-SGCC
*****0.166 | 0.000415 | 0.1258 | 'market group for electricity, medium voltage' (kilowatt hour, CN, Non
******0.146 | 0.0003648 | 0.104 | 'market group for electricity, medium voltage' (kilowatt hour, CN-SGCC
*******0.0414 | 0.0001035 | 0.02382 | 'market for electricity, medium voltage' (kilowatt hour, CN-NCGC, None
*******0.0321 | 8.034e-05 | 0.02828 | 'market for electricity, medium voltage' (kilowatt hour, CN-ECGC, None
*******0.0292 | 7.304e-05 | 0.01542 | 'market for electricity, medium voltage' (kilowatt hour, CN-NECG, None
***0.0361 | 9.026e-05 | 0.02736 | 'market group for electricity, medium voltage' (kilowatt hour, CN, Non
****0.0317 | 7.933e-05 | 0.02263 | 'market group for electricity, medium voltage' (kilowatt hour, CN-SGCC
**0.0934 | 0.0002335 | 0.007125 | 'aluminium alloy production, AlMg3' (kilogram, CN, None)
***0.0839 | 0.0002098 | 0.007125 | 'aluminium alloy production, AlMg3' (kilogram, RER, None)
****0.0695 | 0.000174 | 0.006875 | 'market for aluminium, cast alloy' (kilogram, GLO, None)
*****0.0638 | 0.0001596 | 0.001797 | 'aluminium ingot, primary, to aluminium, cast alloy market' (kilogram,
******0.0547 | 0.0001369 | 0.001396 | 'market for aluminium, primary, ingot' (kilogram, RoW, None)
*******0.0405 | 0.0001012 | 0.0009819 | 'aluminium production, primary, ingot' (kilogram, CN, None)
**0.114 | 0.0002843 | 0.04042 | 'flat glass production, uncoated' (kilogram, CN, None)
***0.11 | 0.0002749 | 0.04042 | 'flat glass production, uncoated' (kilogram, RER, None)
**0.123 | 0.0003068 | 0.0007477 | 'market for copper, cathode' (kilogram, GLO, None)
***0.103 | 0.0002567 | 0.0004523 | 'electrorefining of copper, anode' (kilogram, GLO, None)
****0.101 | 0.0002523 | 0.000449 | 'market for copper, anode' (kilogram, GLO, None)
*****0.0343 | 8.584e-05 | 0.0001675 | 'smelting of copper concentrate, sulfide ore' (kilogram, RoW, None)
*****0.0386 | 9.655e-05 | 0.0001708 | 'smelting of copper concentrate, sulfide ore' (kilogram, CN, None)
```

The Sankey diagram for this output is provided Fig. S15.

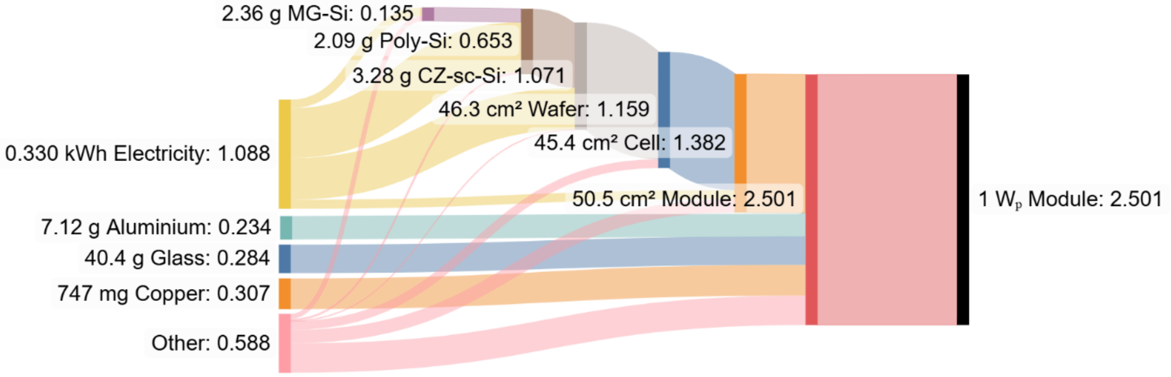

Fig. S15. *Sankey diagram of the supply chain for the production of 1 Watt-peak (W_p) of PERC solar panel capacity. Values behind each colon represent the terrestrial acidification footprint of that product in g SO_2-eq./W_p. MG-Si: metallurgical grade silicon; poly-Si: poly-silicon; Cz-sc-Si: Czochralski single-crystalline silicon.*

Results for the OAT sensitivity analyses of the ReCiPe 2016 (I) acidification midpoint impact category are provided in Tab. S12. The values in rows labelled F, B, and F+B correspond with impact reductions due to developments in the foreground, background, or both, respectively. The percentage change in acidification footprint is calculated between 2020 and 2050 ($\Delta_{2020\rightarrow2050}$). The final eleven rows represent OAT sensitivity values when excluding a single learning curve in the foreground system, where the percentage change in acidification footprint is calculated between “F+B” and “F+B, excl. [a single learning curve (e.g. efficiency)]”. From Tab. S12 it becomes apparent that exclusion of learning in panel efficiency results in the largest increase in acidification footprint and, therefore, the acidification footprint is most sensitive to this process parameter.

Tab. S12. *One-at-a-time sensitivity analyses for the ReCiPe 2016 (I) acidification midpoint impact category showing how sensitive the percentage impact reductions between 2020 and 2050 are to modelled developments in only the foreground system (F), only the background system (B), or both (F+B), and to exclusion of individual learning curves for the foreground system.*

	2020
**Baseline**	**kg SO_2-eq.**
	0.001371

	2050 SSP2-base		2050 SSP2-RCP1.9	
	**kg SO_2-eq.**	**$\Delta_{2020\rightarrow2050}$**	**kg SO_2-eq.**	**$\Delta_{2020\rightarrow2050}$**
**F**	0.001092	−20%	0.001025	−25%
**B**	0.001246	−9%	0.000863	−37%
**F+B**	0.001006	−27%	0.000707	−48%

	**kg SO_2-eq.**	**$\Delta_{FB\rightarrow FB\ excl.}$**	**kg SO_2-eq.**	**$\Delta_{2020\rightarrow2050}$**
**F+B, excluding efficiency**	0.001081	7.5%	0.000777	10.0%
**F+B, excluding thickness wafer**	0.001031	2.5%	0.000718	1.6%
**F+B, excluding thickness kerf**	0.001032	2.7%	0.000718	1.6%
**F+B, excluding thickness glass**	0.001026	2.0%	0.000731	3.5%
**F+B, excluding mass frame**	0.001009	0.3%	0.000711	0.5%
**F+B, excluding mass silver**	0.001019	1.3%	0.000721	1.9%
**F+B, excluding power consumption MG-Si**	0.001007	0.2%	0.000707	0.1%
**F+B, excluding power consumption poly-Si**	0.001017	1.1%	0.000709	0.3%
**F+B, excluding power consumption Cz-sc-Si**	0.001023	1.8%	0.000710	0.5%
**F+B, excluding power consumption cell production**	0.001028	2.2%	0.000712	0.7%
**F+B, excluding power consumption module production**	0.001009	0.3%	0.000708	0.1%

Fig. S16 displays the Spearman's rank correlation coefficients between the acidification footprint of PERC panel production and each of the eleven parameters for which process-specific learning curves were created. The higher the Spearman's rank correlation coefficient of a process parameter, the more it contributes to the uncertainty in the acidification footprint of PERC panel production. Module efficiency has a negative correlation coefficient, meaning that higher module efficiency correlate with lower acidification footprints. The other process parameters all have positive correlation coefficients, meaning that an increase in these parameter values result in a higher acidification footprints.

In the SSP2-base scenarios, the uncertainty in the acidification footprints is most affected by uncertainty in power consumption in module and Czochralski single-crystal silicon production. Sulfur in fossil fuels is released as sulfur dioxide during combustion of said fossil fuels and nitrogen oxides are simultaneously formed. Sulfur dioxide and nitrogen oxides both contribute to acidification. However, in a decarbonized economy (i.e. SSP2-RCP19), fewer fossil fuels are used in energy generation and, therefore, the acidification footprint of the consumed electricity diminishes. This in turn makes the acidification footprint of producing a PERC panel less sensitive to uncertainty in process-specific learning curves for electricity consumption. As a result, the next two main contributors, glass and the frame (see Fig. S15) become the largest contributors to uncertainty in the acidification footprint of producing a PERC panel. The uncertainty in projected acidification footprints from PERC panel production might then be reduced by directing research efforts towards reducing uncertainty for the process-specific learning curve of the thickness of the glass and the mass of the frame, e.g. by collecting more datapoints or by further disaggregating this learning curve into multiple process-specific learning curves for producing a frame.

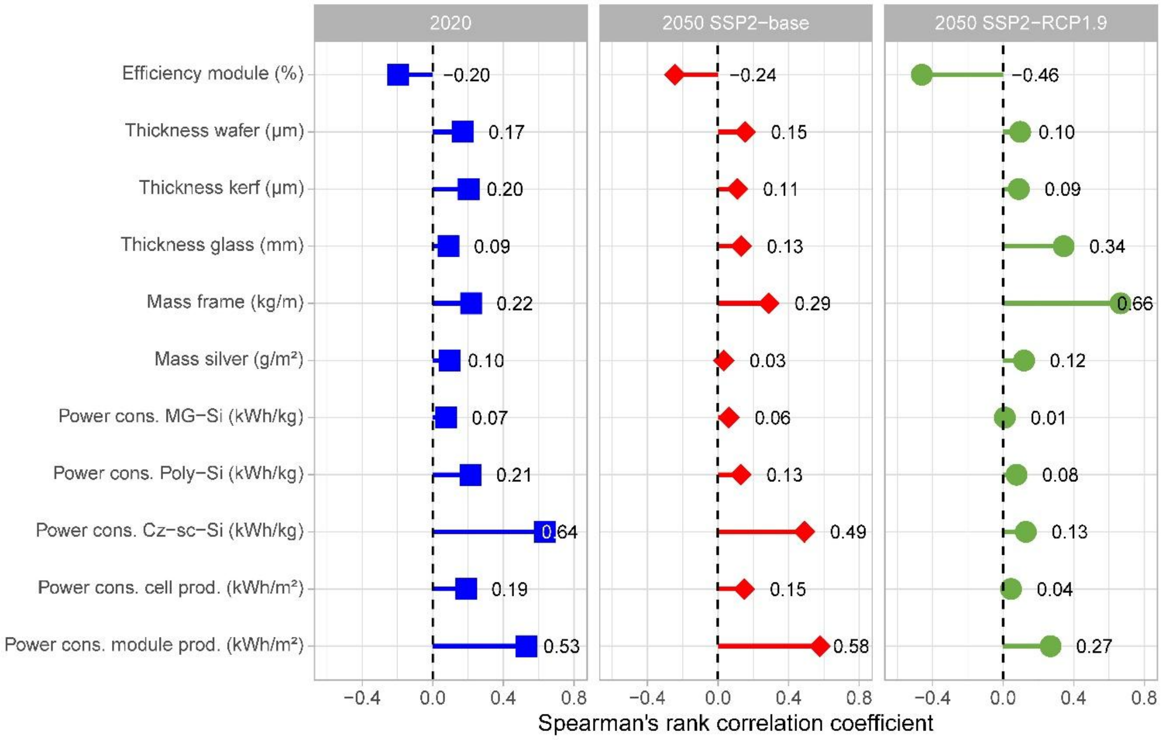

Fig. S16. *Uncertainty analyses showing the Spearman's rank correlation coefficients relating the 1,000 terrestrial acidification footprints obtained for each scenario against the eleven process parameters adapted in the foreground system using learning curves. F: foreground; B: background; MG-Si: metallurgical grade silicon; poly-Si: poly-silicon; Cz-sc-Si: Czochralski single-crystalline silicon; cons.: consumption; prod.: production.*

2.2.4. Photochemical oxidant formation, terrestrial

The output of the *print_recursive_calculation* is as follows:

```
Fraction of score | Absolute score | Amount | Activity
0001 | 0.001992 |     1 | 'glass-backsheet PERC module production' (watt peak, CN, None)
*0001 | 0.001992 | 0.005052 | 'glass-backsheet PERC module production' (square meter, CN, None)
**0.686 | 0.001367 | 0.004537 | 'PERC cell production, mono Si M6 wafer' (square meter, CN, None)
***0.463 | 0.0009218 | 0.004627 | '170 µm mono M6 bricking and wafer production, photovoltaic' (square m
****0.437 | 0.0008702 | 0.003276 | 'p-type silicon production, single crystal, Czochralski process' (kilo
*****0.266 | 0.0005304 | 0.002093 | 'silicon production, solar grade, modified Siemens process' (kilogram,
******0.0543 | 0.0001081 | 0.002365 | 'silicon production, metallurgical grade' (kilogram, CN, None)
*******0.0351 | 6.988e-05 | 0.02602 | 'market group for electricity, medium voltage' (kilowatt hour, CN, Non
******0.203 | 0.0004048 | 0.1507 | 'market group for electricity, medium voltage' (kilowatt hour, CN, Non
*******0.181 | 0.0003601 | 0.1247 | 'market group for electricity, medium voltage' (kilowatt hour, CN-SGCC
*****0.17 | 0.0003379 | 0.1258 | 'market group for electricity, medium voltage' (kilowatt hour, CN, Non
******0.151 | 0.0003005 | 0.104 | 'market group for electricity, medium voltage' (kilowatt hour, CN-SGCC
*******0.0406 | 8.085e-05 | 0.02382 | 'market for electricity, medium voltage' (kilowatt hour, CN-NCGC, None
*******0.0333 | 6.637e-05 | 0.02828 | 'market for electricity, medium voltage' (kilowatt hour, CN-ECGC, None
*******0.0322 | 6.421e-05 | 0.01542 | 'market for electricity, medium voltage' (kilowatt hour, CN-NECG, None
***0.0369 | 7.348e-05 | 0.02736 | 'market group for electricity, medium voltage' (kilowatt hour, CN, Non
****0.0328 | 6.536e-05 | 0.02263 | 'market group for electricity, medium voltage' (kilowatt hour, CN-SGCC
***0.0263 | 5.247e-05 | 1.579e-05 | 'market for metallization paste, front side' (kilogram, RER, None)
****0.0263 | 5.247e-05 | 1.579e-05 | 'metallization paste production, front side' (kilogram, RER, None)
*****0.0263 | 5.245e-05 | 1.323e-05 | 'market for silver' (kilogram, GLO, None)
**0.0802 | 0.0001598 | 0.007125 | 'aluminium alloy production, AlMg3' (kilogram, CN, None)
***0.0686 | 0.0001367 | 0.007125 | 'aluminium alloy production, AlMg3' (kilogram, RER, None)
****0.054 | 0.0001075 | 0.006875 | 'market for aluminium, cast alloy' (kilogram, GLO, None)
*****0.0472 | 9.411e-05 | 0.001797 | 'aluminium ingot, primary, to aluminium, cast alloy market' (kilogram,
******0.042 | 8.369e-05 | 0.001396 | 'market for aluminium, primary, ingot' (kilogram, RoW, None)
*******0.0326 | 6.502e-05 | 0.0009819 | 'aluminium production, primary, ingot' (kilogram, CN, None)
**0.0927 | 0.0001846 | 0.04042 | 'flat glass production, uncoated' (kilogram, CN, None)
***0.0881 | 0.0001754 | 0.04042 | 'flat glass production, uncoated' (kilogram, RER, None)
**0.0253 | 5.032e-05 | 0.0007477 | 'market for copper, cathode' (kilogram, GLO, None)
```

The Sankey diagram for this output is provided Fig. S17.

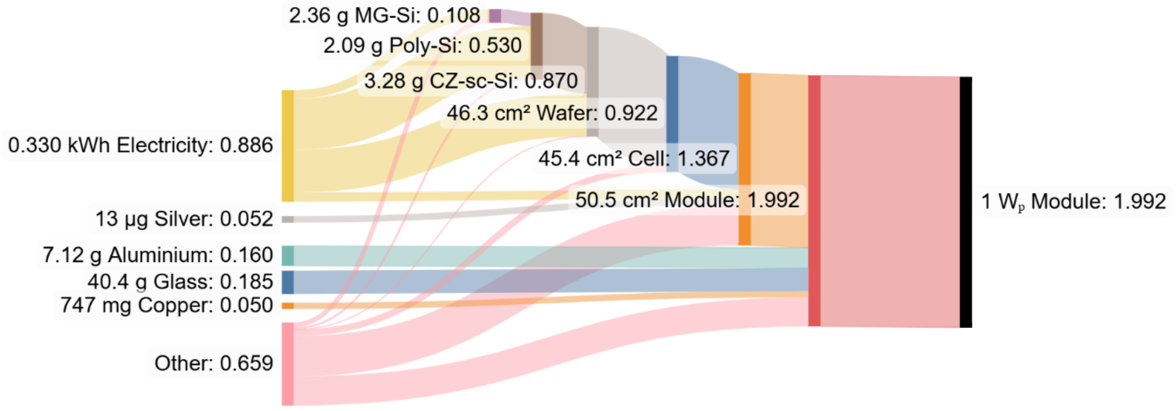

Fig. S17. *Sankey diagram of the supply chain for the production of 1 Watt-peak (W_p) of PERC solar panel capacity. Values behind each colon represent the terrestrial photochemical oxidant formation footprint of that product in g NO_x-eq./W_p. MG-Si: metallurgical grade silicon; poly-Si: poly-silicon; Cz-sc-Si: Czochralski single-crystalline silicon.*

Results for the OAT sensitivity analyses of the ReCiPe 2016 (I) terrestrial photochemical oxidant formation midpoint impact category are provided in Tab. S13. The values in rows labelled F, B, and F+B correspond with impact reductions due to developments in the foreground, background, or both, respectively. The percentage change in terrestrial photochemical oxidant formation footprint is calculated between 2020 and 2050 ($\Delta_{2020\rightarrow2050}$). The final eleven rows represent OAT sensitivity values when excluding a single learning curve in the foreground system, where the percentage change in terrestrial photochemical oxidant formation footprint is calculated between "F+B" and "F+B, excl. [a single learning curve (e.g. efficiency)]". From Tab. S13 it becomes apparent that exclusion of learning in panel efficiency results in the largest increase in terrestrial photochemical oxidant formation footprint and, therefore, the terrestrial photochemical oxidant formation footprint is most sensitive to this process parameter.

Tab. S13. *One-at-a-time sensitivity analyses for the ReCiPe 2016 (I) terrestrial photochemical oxidant formation midpoint impact category showing how sensitive the percentage impact reductions between 2020 and 2050 are to modelled developments in only the foreground system (F), only the background system (B), or both (F+B), and to exclusion of individual learning curves for the foreground system.*

**Baseline**	**2020**
	**kg NO_x-eq.**
	0.001057

	**2050 SSP2-base**		**2050 SSP2-RCP1.9**	
	**kg NO_x-eq.**	**$\Delta_{2020\rightarrow2050}$**	**kg NO_x-eq.**	**$\Delta_{2020\rightarrow2050}$**
**F**	0.00084	−21%	0.000788	−25%
**B**	0.000962	−9%	0.000716	−32%
**F+B**	0.000771	−27%	0.000578	−45%
	**kg NO_x-eq.**	**$\Delta_{FB\rightarrow FB\ excl.}$**	**kg NO_x-eq.**	**$\Delta_{2020\rightarrow2050}$**
**F+B, excluding efficiency**	0.000829	7.5%	0.000635	10.0%
**F+B, excluding thickness wafer**	0.000791	2.6%	0.000588	1.8%
**F+B, excluding thickness kerf**	0.000792	2.7%	0.000588	1.8%
**F+B, excluding thickness glass**	0.000784	1.7%	0.000593	2.7%
**F+B, excluding mass frame**	0.000773	0.2%	0.00058	0.4%
**F+B, excluding mass silver**	0.000788	2.3%	0.000597	3.4%
**F+B, excluding power consumption MG-Si**	0.000772	0.2%	0.000578	0.1%
**F+B, excluding power consumption poly-Si**	0.00078	1.1%	0.00058	0.4%
**F+B, excluding power consumption Cz-sc-Si**	0.000785	1.8%	0.000582	0.7%
**F+B, excluding power consumption cell production**	0.000788	2.3%	0.000583	0.9%
**F+B, excluding power consumption module production**	0.000774	0.3%	0.000579	0.1%

Fig. S18 displays the Spearman's rank correlation coefficients between the terrestrial photochemical oxidant formation footprint of PERC panel production and each of the eleven parameters for which process-specific learning curves were created. The higher the Spearman's rank correlation coefficient of a process parameter, the more it contributes to the uncertainty in the terrestrial photochemical oxidant formation footprint of PERC panel production. Module efficiency has a negative correlation coefficient, meaning that higher module efficiency correlate with lower terrestrial photochemical oxidant formation footprints. The other process parameters all have positive correlation coefficients, meaning that an increase in these parameter values result in a higher terrestrial photochemical oxidant formation footprints.

In the SSP2-base scenarios, the uncertainty in the terrestrial photochemical oxidant formation footprints is most affected by uncertainty in power consumption in module and Czochralski single-crystal silicon production. In a decarbonized economy (i.e. SSP2-RCP19), one would expect the terrestrial photochemical oxidant formation footprint of the consumed electricity to diminish as more electricity is generated with renewable resources. As the consumption of fossil resources in electricity generation decreases, the terrestrial photochemical oxidant formation footprint of producing a PERC panel would become less sensitive to uncertainty in process-specific learning curves for electricity consumption. A slight reduction is visible, but not as strong as e.g. in the categories of climate change and particulate matter formation. A reason for this can be found in the scenario files that are exported by premise (see the Supporting Information). Contributions of various sources of electricity are displayed in Tab. S9. In 2020, the major energy sources for electricity are pulverized coal and hydro. In the SSP2-RCP1.9 scenario for 2050, the main contributors are onshore wind, solar, hydro and natural gas with carbon capture and storage (CCS). The use of natural gas with CCS results in fewer emissions of greenhouse gasses and particulate matter, thus explaining the reduced contribution of electricity to the uncertainty in the GHG footprint and particulate matter formation footprints in the 20205 SSP2-RCP1.9 scenario. However, use of natural gas results in fugitive emissions of methane along its value chain, where methane can react with nitrous oxides to form ozone. This is why electricity consumption remains a relatively large contributor to uncertainty in the terrestrial photochemical oxidant formation footprints.

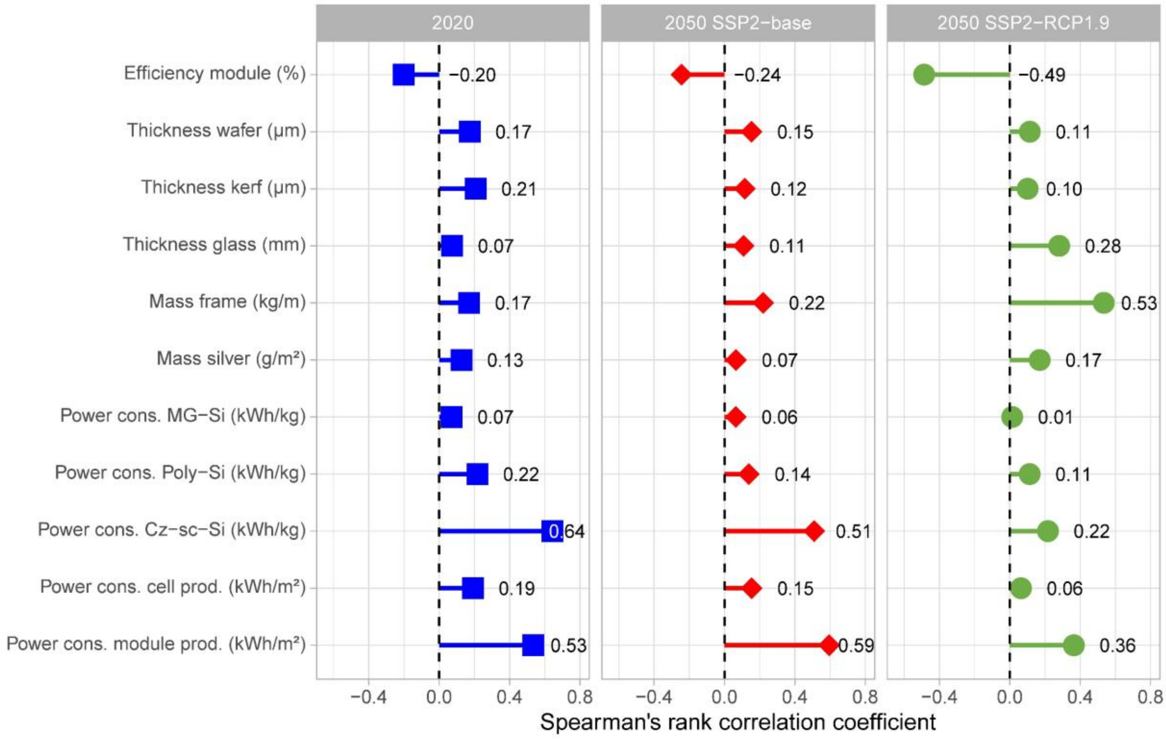

Fig. S18. *Uncertainty analyses showing the Spearman's rank correlation coefficients relating the 1,000 terrestrial photochemical oxidant formation footprints obtained for each scenario against the eleven process parameters adapted in the foreground system using learning curves. F: foreground; B: background; MG-Si: metallurgical grade silicon; poly-Si: poly-silicon; Cz-sc-Si: Czochralski single-crystalline silicon; cons.: consumption; prod.: production.*

2.2.5. Particulate Matter Formation

The output of the *print_recursive_calculation* is as follows:

```
Fraction of score | Absolute score | Amount | Activity
0001 | 0.0003505 |     1 | 'glass-backsheet PERC module production' (watt peak, CN, None)
*0001 | 0.0003505 | 0.005052 | 'glass-backsheet PERC module production' (square meter, CN, None)
**0.637 | 0.0002233 | 0.004537 | 'PERC cell production, mono Si M6 wafer' (square meter, CN, None)
***0.492 | 0.0001726 | 0.004627 | '170 µm mono M6 bricking and wafer production, photovoltaic' (square m
****0.463 | 0.0001622 | 0.003276 | 'p-type silicon production, single crystal, Czochralski process' (kilo
*****0.276 | 9.668e-05 | 0.002093 | 'silicon production, solar grade, modified Siemens process' (kilogram,
******0.0527 | 1.847e-05 | 0.002365 | 'silicon production, metallurgical grade' (kilogram, CN, None)
*******0.0373 | 1.307e-05 | 0.02602 | 'market group for electricity, medium voltage' (kilowatt hour, CN, Non
******0.216 | 7.573e-05 | 0.1507 | 'market group for electricity, medium voltage' (kilowatt hour, CN, Non
*******0.193 | 6.755e-05 | 0.1247 | 'market group for electricity, medium voltage' (kilowatt hour, CN-SGCC
*****0.18 | 6.321e-05 | 0.1258 | 'market group for electricity, medium voltage' (kilowatt hour, CN, Non
******0.161 | 5.638e-05 | 0.104 | 'market group for electricity, medium voltage' (kilowatt hour, CN-SGCC
*******0.043 | 1.507e-05 | 0.02382 | 'market for electricity, medium voltage' (kilowatt hour, CN-NCGC, None
*******0.0349 | 1.222e-05 | 0.02828 | 'market for electricity, medium voltage' (kilowatt hour, CN-ECGC, None
*******0.0351 | 1.23e-05 | 0.01542 | 'market for electricity, medium voltage' (kilowatt hour, CN-NECG, None
***0.0392 | 1.375e-05 | 0.02736 | 'market group for electricity, medium voltage' (kilowatt hour, CN, Non
****0.035 | 1.226e-05 | 0.02263 | 'market group for electricity, medium voltage' (kilowatt hour, CN-SGCC
***0.0289 | 1.011e-05 | 1.284e-05 | 'market for silicon tetrahydride' (kilogram, GLO, None)
****0.0289 | 1.011e-05 | 1.284e-05 | 'silicon hydrochloration' (kilogram, GLO, None)
*****0.0252 | 8.818e-06 | 0.01141 | 'market group for electricity, high voltage' (kilowatt hour, GLO, None
**0.159 | 5.562e-05 | 0.007125 | 'aluminium alloy production, AlMg3' (kilogram, CN, None)
***0.147 | 5.144e-05 | 0.007125 | 'aluminium alloy production, AlMg3' (kilogram, RER, None)
****0.0731 | 2.562e-05 | 0.006875 | 'market for aluminium, cast alloy' (kilogram, GLO, None)
*****0.0683 | 2.393e-05 | 0.001797 | 'aluminium ingot, primary, to aluminium, cast alloy market' (kilogram,
******0.0617 | 2.161e-05 | 0.001396 | 'market for aluminium, primary, ingot' (kilogram, RoW, None)
*******0.0338 | 1.186e-05 | 0.0009819 | 'aluminium production, primary, ingot' (kilogram, CN, None)
****0.065 | 2.279e-05 | 0.0002173 | 'market for magnesium' (kilogram, GLO, None)
*****0.0649 | 2.275e-05 | 0.00018 | 'magnesium production, pidgeon process' (kilogram, CN, None)
**0.0422 | 1.481e-05 | 0.04042 | 'flat glass production, uncoated' (kilogram, CN, None)
***0.0375 | 1.315e-05 | 0.04042 | 'flat glass production, uncoated' (kilogram, RER, None)
**0.0325 | 1.14e-05 | 0.0007477 | 'market for copper, cathode' (kilogram, GLO, None)
***0.0275 | 9.638e-06 | 0.0004523 | 'electrorefining of copper, anode' (kilogram, GLO, None)
```

The Sankey diagram for this output is provided Fig. S19.

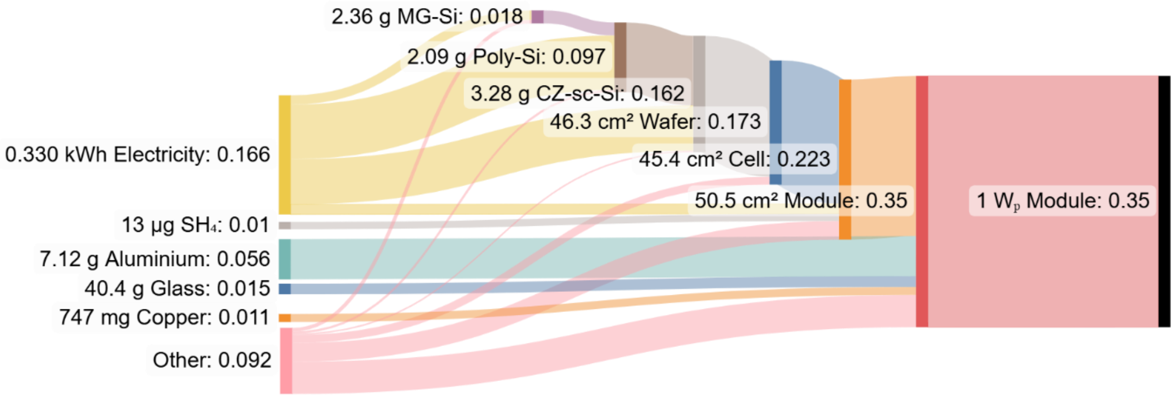

Fig. S19. *Sankey diagram of the supply chain for the production of 1 Watt-peak (W_p) of PERC solar panel capacity. Values behind each colon represent the particulate matter formation footprint of that product in g PM2.5-eq./W_p. MG-Si: metallurgical grade silicon; poly-Si: poly-silicon; Cz-sc-Si: Czochralski single-crystalline silicon.*

Results for the OAT sensitivity analyses of the ReCiPe 2016 (I) particulate matter formation midpoint impact category are provided in Tab. S14. The values in rows labelled F, B, and F+B correspond with impact reductions due to developments in the foreground, background, or both, respectively. The percentage change in particulate matter formation footprint is calculated between 2020 and 2050 ($\Delta_{2020\rightarrow2050}$). The final eleven rows represent OAT sensitivity values when excluding a single learning curve in the foreground system, where the percentage change in particulate matter formation footprint is calculated between "F+B" and "F+B, excl. [a single learning curve (e.g. efficiency)]". From Tab. S14 it becomes apparent that exclusion of learning in panel efficiency results in the largest increase in particulate matter formation footprint and, therefore, the particulate matter formation footprint is most sensitive to this process parameter.

Tab. S14. *One-at-a-time sensitivity analyses for the ReCiPe 2016 (I) particulate matter formation midpoint impact category showing how sensitive the percentage impact reductions between 2020 and 2050 are to modelled developments in only the foreground system (F), only the background system (B), or both (F+B), and to exclusion of individual learning curves for the foreground system.*

**Baseline**	**2020**
	**kg PM2.5-eq.**
	0.0001598

	**2050 SSP2-base**		**2050 SSP2-RCP1.9**	
	**kg PM2.5-eq.**	**$\Delta_{2020\rightarrow2050}$**	**kg PM2.5-eq.**	**$\Delta_{2020\rightarrow2050}$**
**F**	0.0001192	−25%	0.0001099	−31%
**B**	0.0001162	−27%	0.0000709	−56%
**F+B**	0.0000900	−44%	0.0000572	−64%

	**kg PM2.5-eq.**	**$\Delta_{FB\rightarrow FB\ excl.}$**	**kg PM2.5-eq.**	**$\Delta_{2020\rightarrow2050}$**
**F+B, excluding efficiency**	0.0000968	7.5%	0.0000628	10.0%
**F+B, excluding thickness wafer**	0.0000933	3.6%	0.0000585	2.3%
**F+B, excluding thickness kerf**	0.0000935	3.8%	0.0000585	2.3%
**F+B, excluding thickness glass**	0.0000909	1.0%	0.0000582	1.8%
**F+B, excluding mass frame**	0.0000907	0.7%	0.0000579	1.3%
**F+B, excluding mass silver**	0.0000913	1.4%	0.0000585	2.3%
**F+B, excluding power consumption MG-Si**	0.0000903	0.3%	0.0000572	0.1%
**F+B, excluding power consumption poly-Si**	0.0000915	1.6%	0.0000574	0.5%
**F+B, excluding power consumption Cz-sc-Si**	0.0000924	2.6%	0.0000576	0.7%
**F+B, excluding power consumption cell production**	0.0000930	3.3%	0.0000577	0.9%
**F+B, excluding power consumption module production**	0.0000905	0.5%	0.0000572	0.1%

Fig. S20 displays the Spearman's rank correlation coefficients between the particulate matter formation footprint for PERC panel production and each of the eleven parameters for which process-specific learning curves were created. The higher the Spearman's rank correlation coefficient of a process parameter, the more it contributes to the uncertainty in the particulate matter formation footprint of PERC panel production. Module efficiency has a negative correlation coefficient, meaning that higher module efficiency correlate with lower particulate matter formation footprints. The other process parameters all have positive correlation coefficients, meaning that an increase in these parameter values result in a higher particulate matter formation footprints.

In the SSP2-base scenarios, the uncertainty in the particulate matter formation footprints is most affected by uncertainty in power consumption in module and Czochralski single-crystal silicon production. Particulate matter is mostly formed in electricity generation during combustion of fossil fuels. However, in a decarbonized economy (i.e. SSP2-RCP19), fewer fossil fuels are used in electricity generation and, therefore, the particulate matter formation footprint of the consumed electricity diminishes. The particulate matter formation footprint of producing a PERC panel thus becomes less sensitive to uncertainty in process-specific learning curves for electricity consumption. As a result, the next main contributors, the frame (see Fig. S19) becomes the largest contributors to uncertainty in the particulate matter formation footprint of producing a PERC panel. The uncertainty in projected particulate matter formation footprint of PERC panel production might then be reduced by directing research efforts towards reducing uncertainty for the process-specific learning curve of the mass of the frame, e.g. by collecting more datapoints or by further disaggregating this learning curve into multiple process-specific learning curves for producing a frame.

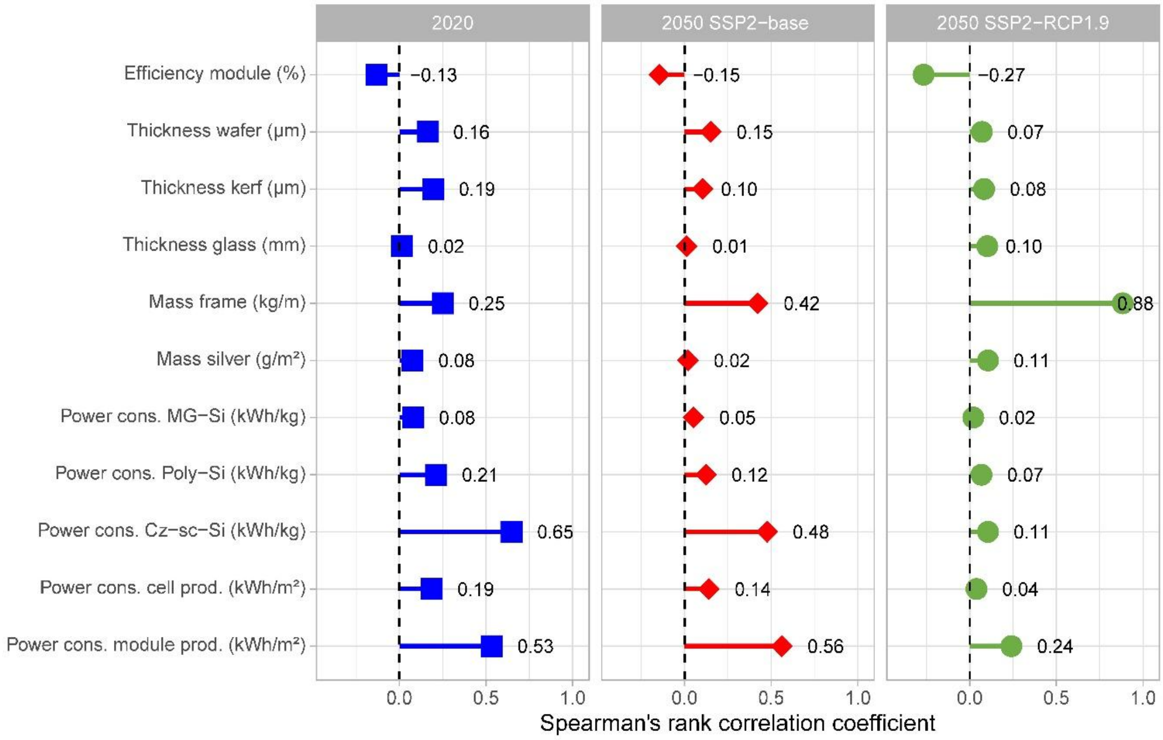

Fig. S20. *Uncertainty analyses showing the Spearman's rank correlation coefficients relating the 1,000 particulate matter formation footprints obtained for each scenario against the eleven process parameters adapted in the foreground system using learning curves. F: foreground; B: background; MG-Si: metallurgical grade silicon; poly-Si: poly-silicon; Cz-sc-Si: Czochralski single-crystalline silicon; cons.: consumption; prod.: production.*

2.2.6. Energy Resources: Non-renewable, Fossil

The output of the *print_recursive_calculation* is as follows:

```
Fraction of score | Absolute score | Amount | Activity
0001 | 0.1345 |     1 | 'glass-backsheet PERC module production' (watt peak, CN, None)
*0001 | 0.1345 | 0.005052 | 'glass-backsheet PERC module production' (square meter, CN, None)
**0.63 | 0.08474 | 0.004537 | 'PERC cell production, mono Si M6 wafer' (square meter, CN, None)
***0.53 | 0.0713 | 0.004627 | '170 µm mono M6 bricking and wafer production, photovoltaic' (square m
****00.5 | 0.06718 | 0.003276 | 'p-type silicon production, single crystal, Czochralski process' (kilo
*****0.313 | 0.0421 | 0.002093 | 'silicon production, solar grade, modified Siemens process' (kilogram,
******0.0559 | 0.007515 | 0.002365 | 'silicon production, metallurgical grade' (kilogram, CN, None)
*******0.0383 | 0.005149 | 0.02602 | 'market group for electricity, medium voltage' (kilowatt hour, CN, Non
******0.0283 | 0.003809 | 0.1465 | 'heat production, natural gas, at industrial furnace >100kW' (megajoul
******0.222 | 0.02982 | 0.1507 | 'market group for electricity, medium voltage' (kilowatt hour, CN, Non
*******0.196 | 0.02629 | 0.1247 | 'market group for electricity, medium voltage' (kilowatt hour, CN-SGCC
*******0.0263 | 0.003536 | 0.02605 | 'market for electricity, medium voltage' (kilowatt hour, CN-CSG, None)
*****0.185 | 0.02489 | 0.1258 | 'market group for electricity, medium voltage' (kilowatt hour, CN, Non
******0.163 | 0.02194 | 0.104 | 'market group for electricity, medium voltage' (kilowatt hour, CN-SGCC
*******0.044 | 0.005921 | 0.02382 | 'market for electricity, medium voltage' (kilowatt hour, CN-NCGC, None
*******0.0384 | 0.005167 | 0.02828 | 'market for electricity, medium voltage' (kilowatt hour, CN-ECGC, None
*******0.0337 | 0.00453 | 0.01542 | 'market for electricity, medium voltage' (kilowatt hour, CN-NECG, None
***0.0403 | 0.005414 | 0.02736 | 'market group for electricity, medium voltage' (kilowatt hour, CN, Non
****0.0355 | 0.004772 | 0.02263 | 'market group for electricity, medium voltage' (kilowatt hour, CN-SGCC
**0.0947 | 0.01274 | 0.007125 | 'aluminium alloy production, AlMg3' (kilogram, CN, None)
***0.0862 | 0.01159 | 0.007125 | 'aluminium alloy production, AlMg3' (kilogram, RER, None)
****0.0633 | 0.008507 | 0.006875 | 'market for aluminium, cast alloy' (kilogram, GLO, None)
*****0.0558 | 0.007504 | 0.001797 | 'aluminium ingot, primary, to aluminium, cast alloy market' (kilogram,
******0.0487 | 0.006546 | 0.001396 | 'market for aluminium, primary, ingot' (kilogram, RoW, None)
*******0.034 | 0.004568 | 0.0009819 | 'aluminium production, primary, ingot' (kilogram, CN, None)
**0.0753 | 0.01013 | 0.04042 | 'flat glass production, uncoated' (kilogram, CN, None)
***0.072 | 0.009679 | 0.04042 | 'flat glass production, uncoated' (kilogram, RER, None)
****0.0337 | 0.004531 | 0.004692 | 'market group for natural gas, high pressure' (cubic meter, Europe wit
**0.0549 | 0.007385 | 0.004006 | 'market for ethylvinylacetate, foil' (kilogram, GLO, None)
***0.0371 | 0.004982 | 0.002682 | 'ethylvinylacetate production, foil' (kilogram, RoW, None)
****0.0342 | 0.004599 | 0.002735 | 'market for ethylene vinyl acetate copolymer' (kilogram, RoW, None)
*****0.0336 | 0.004524 | 0.002735 | 'ethylene vinyl acetate copolymer production' (kilogram, RoW, None)
```

The Sankey diagram for this output is provided Fig. S21.

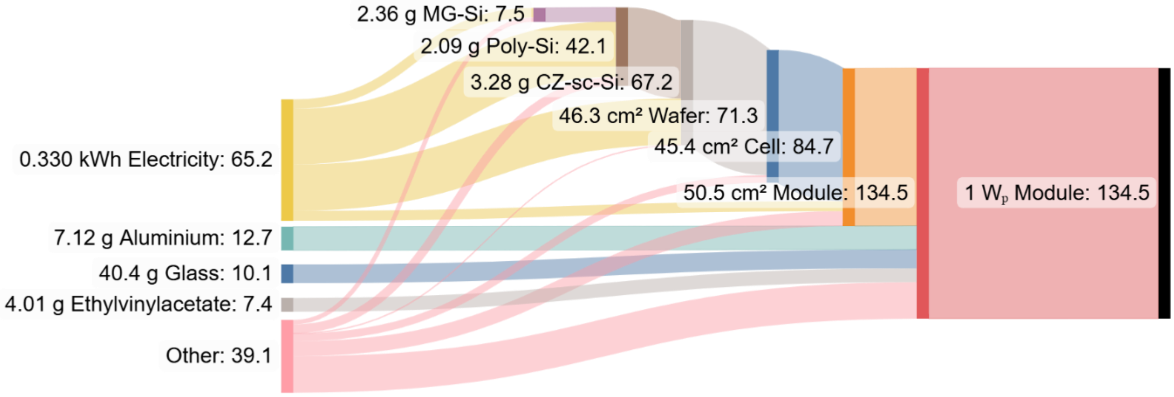

Fig. S21. *Sankey diagram of the supply chain for the production of 1 Watt-peak (W_p) of of PERC solar panel capacity. Values behind each colon represent the non-renewable fossil energy resource footprint of that product in g oil-Eq./W_p. MG-Si: metallurgical grade silicon; poly-Si: poly-silicon; Cz-sc-Si: Czochralski single-crystalline silicon.*

Results for the OAT sensitivity analyses of the ReCiPe 2016 (I) non-renewable fossil energy resources midpoint impact category are provided in Tab. S15. The values in rows labelled F, B, and F+B correspond with impact reductions due to developments in the foreground, background, or both, respectively. The percentage change in non-renewable fossil energy resources footprint is calculated between 2020 and 2050 ($\Delta_{2020\rightarrow2050}$). The final eleven rows represent OAT sensitivity values when excluding a single learning curve in the foreground system, where the percentage change in non-renewable fossil energy resources footprint is calculated between "F+B" and "F+B, excl. [a single learning curve (e.g. efficiency)]". From Tab. S15 it becomes apparent that exclusion of learning in panel efficiency results in the largest increase in non-renewable fossil energy resources footprint and, therefore, the non-renewable fossil energy resources footprint is most sensitive to this process parameter.

Tab. S15. *One-at-a-time sensitivity analyses for the ReCiPe 2016 (I) non-renewable fossil energy resources midpoint impact category showing how sensitive the percentage impact reductions between 2020 and 2050 are to modelled developments in only the foreground system (F), only the background system (B), or both (F+B), and to exclusion of individual learning curves for the foreground system.*

	2020			
**Baseline**	**kg oil-eq.**			
	0.09639567			
	**2050 SSP2-base**		**2050 SSP2-RCP1.9**	
	**kg oil-eq.**	**$\Delta_{2020\rightarrow2050}$**	**kg oil-eq.**	**$\Delta_{2020\rightarrow2050}$**
**F**	0.071628	−26%	0.065913	−32%
**B**	0.088693	−8%	0.048759	−49%
**F+B**	0.066482	−31%	0.037014	−62%
	**kg oil-eq.**	**$\Delta_{FB\rightarrow FB\ excl.}$**	**kg oil-eq.**	**$\Delta_{2020\rightarrow2050}$**
**F+B, excluding efficiency**	0.071488	7.5%	0.040701	10.0%
**F+B, excluding thickness wafer**	0.069607	4.7%	0.038585	4.2%
**F+B, excluding thickness kerf**	0.069743	4.9%	0.038605	4.3%
**F+B, excluding thickness glass**	0.067245	1.1%	0.037900	2.4%
**F+B, excluding mass frame**	0.066688	0.3%	0.037225	0.6%
**F+B, excluding mass silver**	0.066991	0.8%	0.037333	0.9%
**F+B, excluding power consumption MG-Si**	0.066706	0.3%	0.037086	0.2%
**F+B, excluding power consumption poly-Si**	0.067892	2.1%	0.037472	1.2%
**F+B, excluding power consumption Cz-sc-Si**	0.068731	3.4%	0.037716	1.9%
**F+B, excluding power consumption cell production**	0.069319	4.3%	0.037958	2.6%
**F+B, excluding power consumption module production**	0.066917	0.7%	0.037163	0.4%

Fig. S22 displays the Spearman's rank correlation coefficients between the non-renewable fossil energy resources footprint for PERC panel production and each of the eleven parameters for which process-specific learning curves were created. The higher the Spearman's rank correlation coefficient of a process parameter, the more it contributes to the uncertainty in the non-renewable fossil energy resources footprint of PERC panel production. Module efficiency has a negative correlation coefficient, meaning that higher module efficiency correlate with lower non-renewable fossil energy resources footprints. The other process parameters all have positive correlation coefficients, meaning that an increase in these parameter values result in a higher non-renewable fossil energy resources footprints.

In the SSP2-base scenarios, the uncertainty in the non-renewable fossil energy resources footprint for PERC panel production is most affected by uncertainty in power consumption in module and Czochralski single-crystal silicon production. In a decarbonized economy (i.e. SSP2-RCP19), one would expect the non-renewable fossil energy resources footprint of the consumed electricity to diminish as more electricity is generated with renewable resources. As the consumption of fossil resources in electricity generation decreases, the non-renewable fossil energy resources footprint of producing a PERC panel would become less sensitive to uncertainty in process-specific learning curves for electricity consumption. A slight reduction is visible, but not as strong as e.g. in the categories of climate change and particulate matter formation. A reason for this can be found in the scenario files that are exported by premise (see the Supporting Information). Contributions of various sources of electricity are displayed in Tab. S9. In 2020, the major energy sources for electricity are pulverized coal and hydro. In the SSP2-RCP1.9 scenario for 2050, the main contributors are onshore wind, solar, hydro and natural gas with carbon capture and storage (CCS). The use of natural gas with CCS results in fewer emissions of greenhouse gasses and particulate matter, thus explaining the reduced contribution of electricity to the uncertainty in the GHG footprint and particulate matter formation footprints in the 20205 SSP2-RCP1.9 scenario. However, natural gas with CC still results in consumption of natural gas, thus contributing to the non-renewable fossil energy resources footprints. This is why electricity consumption remains a relatively large contributor to uncertainty in the non-renewable fossil energy resources footprints.

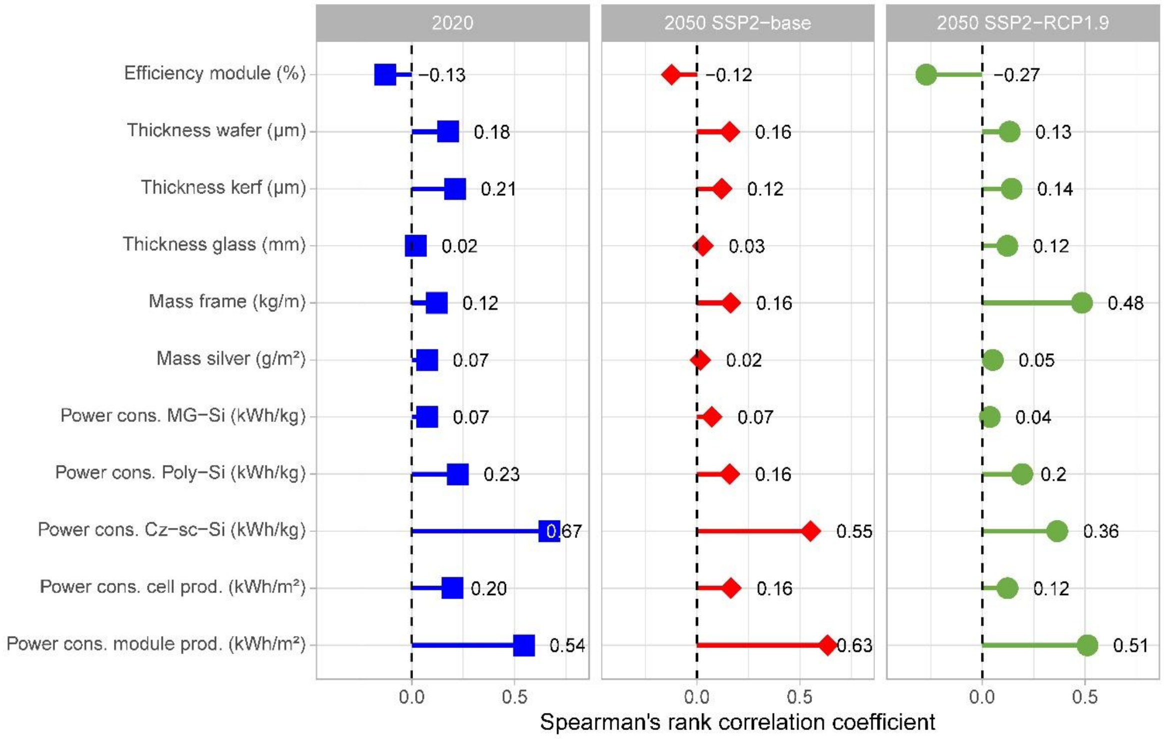

Fig. S22. *Uncertainty analyses showing the Spearman's rank correlation coefficients relating the 1,000 non-renewable fossil energy resource footprints obtained for each scenario against the eleven process parameters adapted in the foreground system using learning curves. F: foreground; B: background; MG-Si: metallurgical grade silicon; poly-Si: poly-silicon; Cz-sc-Si: Czochralski single-crystalline silicon; cons.: consumption; prod.: production.*

2.3. ReCiPe 2016 midpoint egalitarian (E)

2.3.1. Midpoint-to-Endpoint contribution analysis

A midpoint-to-endpoint contribution analysis for the production of 1 W_p of PERC solar panel was conducted using the ReCiPe 2016 endpoint life cycle impact assessment method [59, 60]. Adjustment to the characterization factors of carbon dioxide, methane, and hydrogen in the impact category of Climate Change were made in line with van der Hulst et. al 2024 [61]. Tab. S16 displays contributions of the eighteen midpoint categories to the three end-point categories for the egalitarian perspective. Midpoint categories contributing 15% or more to the total impact of either of the three endpoint categories are highlighted in gray and were included in further assessments at the midpoint level.

Tab. S16. *Midpoint-to-endpoint contribution analysis for producing 1 Watt-peak (W_p) of PERC solar panel capacity using the ReCiPe 2016 egalitarian (E) impact assessment method.*

**Endpoint**, midpoint	**Unit**	**Impact per W_p**	**Contribution**
**Ecosystem quality**	**species*yr**	**4.47E-08**	**100%**
Acidification: terrestrial, terrestrial acidification potential (TAP)	species*yr	5.30E-10	1%
Climate change: freshwater, global warming potential (GWP)	species*yr	3.49E-13	0%
Climate change: terrestrial, global warming potential (GWP)	species*yr	1.28E-08	29%
Ecotoxicity: freshwater, freshwater ecotoxicity potential (FETP)	species*yr	5.29E-11	0%
Ecotoxicity: marine, marine ecotoxicity potential (METP)	species*yr	3.06E-08	68%
Ecotoxicity: terrestrial, terrestrial ecotoxicity potential (TETP)	species*yr	4.55E-11	0%
Eutrophication: freshwater, freshwater eutrophication potential (FEP)	species*yr	1.16E-10	0%
Eutrophication: marine, marine eutrophication potential (MEP)	species*yr	7.11E-14	0%
Land use, agricultural land occupation (LOP)	species*yr	2.20E-10	0%
Photochemical oxidant formation: terrestrial ecosystems, photochemical oxidant formation potential: ecosystems (EOFP)	species*yr	2.57E-10	1%
Water use, water consumption potential (WCP)	species*yr	3.98E-15	0%
**Human Health**	**DALY**	**6.98E-05**	**100%**
Climate change, global warming potential (GWP)	DALY	6.39E-06	9%
Human toxicity: carcinogenic, human toxicity potential (HTPc)	DALY	8.55E-06	12%
Human toxicity: non-carcinogenic, human toxicity potential (HTPnc)	DALY	5.42E-05	78%
Ionising radiation, ionising radiation potential (IRP)	DALY	4.79E-10	0%
Ozone depletion, ozone depletion potential (ODPinfinite)	DALY	3.86E-10	0%
Particulate matter formation, particulate matter formation potential (PMFP)	DALY	6.73E-07	1%
Photochemical oxidant formation: human health, photochemical oxidant formation potential: humans (HOFP)	DALY	1.69E-09	0%
Water use, water consumption potential (WCP)	DALY	1.46E-08	0%
**Natural resources**	**USD2013**	**0.031**	**100%**
Energy resources: non-renewable, fossil, fossil fuel potential (FFP)	USD2013	0.027	86%
Material resources: metals/minerals, surplus ore potential (SOP)	USD2013	0.0044	14%

2.3.2. Climate change

The output of the *print_recursive_calculation* is as follows:

```
Fraction of score | Absolute score | Amount | Activity
0001 | 0.5112 |     1 | 'glass-backsheet PERC module production' (watt peak, CN, None)
*0001 | 0.5112 | 0.005052 | 'glass-backsheet PERC module production' (square meter, CN, None)
**0.703 | 0.3596 | 0.004537 | 'PERC cell production, mono Si M6 wafer' (square meter, CN, None)
***0.563 | 0.2877 | 0.004627 | '170 µm mono M6 bricking and wafer production, photovoltaic' (square m
****0.536 | 0.2741 | 0.003276 | 'p-type silicon production, single crystal, Czochralski process' (kilo
*****0.332 | 0.1696 | 0.002093 | 'silicon production, solar grade, modified Siemens process' (kilogram,
******0.0638 | 0.03262 | 0.002365 | 'silicon production, metallurgical grade' (kilogram, CN, None)
*******0.042 | 0.02146 | 0.02602 | 'market group for electricity, medium voltage' (kilowatt hour, CN, Non
******0.243 | 0.1243 | 0.1507 | 'market group for electricity, medium voltage' (kilowatt hour, CN, Non
*******0.215 | 0.1101 | 0.1247 | 'market group for electricity, medium voltage' (kilowatt hour, CN-SGCC
*******0.0277 | 0.01416 | 0.02605 | 'market for electricity, medium voltage' (kilowatt hour, CN-CSG, None)
*****0.203 | 0.1038 | 0.1258 | 'market group for electricity, medium voltage' (kilowatt hour, CN, Non
******0.18 | 0.09193 | 0.104 | 'market group for electricity, medium voltage' (kilowatt hour, CN-SGCC
*******0.0484 | 0.02476 | 0.02382 | 'market for electricity, medium voltage' (kilowatt hour, CN-NCGC, None
*******0.0412 | 0.02105 | 0.02828 | 'market for electricity, medium voltage' (kilowatt hour, CN-ECGC, None
*******0.0377 | 0.01927 | 0.01542 | 'market for electricity, medium voltage' (kilowatt hour, CN-NECG, None
*******0.0263 | 0.01347 | 0.01536 | 'market for electricity, medium voltage' (kilowatt hour, CN-NWG, None)
***0.0441 | 0.02256 | 0.02736 | 'market group for electricity, medium voltage' (kilowatt hour, CN, Non
****0.0391 | 0.01999 | 0.02263 | 'market group for electricity, medium voltage' (kilowatt hour, CN-SGCC
**0.101 | 0.05171 | 0.007125 | 'aluminium alloy production, AlMg3' (kilogram, CN, None)
***0.0904 | 0.04622 | 0.007125 | 'aluminium alloy production, AlMg3' (kilogram, RER, None)
****0.0706 | 0.03607 | 0.006875 | 'market for aluminium, cast alloy' (kilogram, GLO, None)
*****0.0631 | 0.03224 | 0.001797 | 'aluminium ingot, primary, to aluminium, cast alloy market' (kilogram,
******0.0554 | 0.0283 | 0.001396 | 'market for aluminium, primary, ingot' (kilogram, RoW, None)
*******0.0406 | 0.02076 | 0.0009819 | 'aluminium production, primary, ingot' (kilogram, CN, None)
**0.0791 | 0.04044 | 0.04042 | 'flat glass production, uncoated' (kilogram, CN, None)
***0.0748 | 0.03826 | 0.04042 | 'flat glass production, uncoated' (kilogram, RER, None)
**0.0271 | 0.01383 | 0.01677 | 'market group for electricity, medium voltage' (kilowatt hour, CN, Non
```

The Sankey diagram for this output is provided Fig. S23.

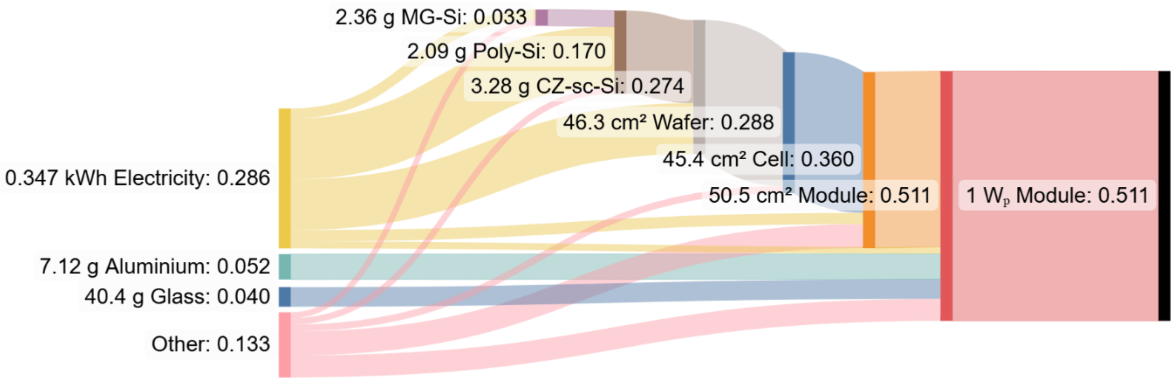

Fig. S23. *Sankey diagram of the supply chain for the production of 1 Watt-peak (W_p) of PERC solar panel capacity. Values behind each colon represent the GHG footprint of that product in kg CO_2-eq./W_p. MG-Si: metallurgical grade silicon; poly-Si: poly-silicon; Cz-sc-Si: Czochralski single-crystalline silicon.*

Results for the OAT sensitivity analyses of the ReCiPe 2016 (E) climate change midpoint impact category are provided in Tab. S17. The values in rows labelled F, B, and F+B correspond with impact reductions due to developments in the foreground, background, or both, respectively. The percentage change in GHG footprint is calculated between 2020 and 2050 ($\Delta_{2020\rightarrow2050}$). The final eleven rows represent OAT sensitivity values when excluding a single learning curve in the foreground system, where the percentage change in GHG footprint is calculated between "F+B" and "F+B, excl. [a single learning curve (e.g. efficiency)]". From Tab. S17 it becomes apparent that exclusion of learning in panel efficiency results in the largest increase in GHG footprint and, therefore, the GHG footprint is most sensitive to this process parameter.

Tab. S17. *One-at-a-time sensitivity analyses for the ReCiPe 2016 (E) climate change midpoint impact category showing how sensitive the percentage impact reductions between 2020 and 2050 are to modelled developments in only the foreground system (F), only the background system (B), or both (F+B), and to exclusion of individual learning curves for the foreground system.*

	**2020**			
**Baseline**	**kg CO_2-eq.**			
	0.401726463			
	**2050 SSP2-base**		**2050 SSP2-RCP1.9**	
	**kg CO_2-eq.**	**$\Delta_{2020\rightarrow2050}$**	**kg CO_2-eq.**	**$\Delta_{2020\rightarrow2050}$**
**F**	0.289633	−28%	0.264061	−34%
**B**	0.356252	−11%	0.100593	−75%
**F+B**	0.259681	−35%	0.082144	−80%
	**kg CO_2-eq.**	**$\Delta_{FB\rightarrow FB\ excl.}$**	**kg CO_2-eq.**	**$\Delta_{2020\rightarrow2050}$**
**F+B, excluding efficiency**	0.279232	7.5%	0.090327	10.0%
**F+B, excluding thickness wafer**	0.273382	5.3%	0.084537	2.9%
**F+B, excluding thickness kerf**	0.273977	5.5%	0.084569	3.0%
**F+B, excluding thickness glass**	0.262692	1.2%	0.085456	4.0%
**F+B, excluding mass frame**	0.260583	0.3%	0.082972	1.0%
**F+B, excluding mass silver**	0.26153	0.7%	0.0826	0.6%
**F+B, excluding power consumption MG-Si**	0.260722	0.4%	0.082142	0.0%
**F+B, excluding power consumption poly-Si**	0.266241	2.5%	0.082131	0.0%
**F+B, excluding power consumption Cz-sc-Si**	0.270148	4.0%	0.082124	0.0%
**F+B, excluding power consumption cell production**	0.272882	5.1%	0.082117	0.0%
**F+B, excluding power consumption module production**	0.261706	0.8%	0.082139	0.0%

Fig. S24 displays the Spearman's rank correlation coefficients between the GHG footprint of PERC panel production and each of the eleven parameters for which process-specific learning curves were created. The higher the Spearman's rank correlation coefficient of a process parameter, the more it contributes to the uncertainty in the GHG footprint of PERC panel production. Module efficiency has a negative correlation coefficient, meaning that higher module efficiency correlate with lower GHG footprints. The other process parameters all have positive correlation coefficients, meaning that an increase in these parameter values result in a higher GHG footprints.

In the SSP2-base scenarios, the uncertainty in the GHG footprints is most affected by uncertainty in power consumption in module and Czochralski single-crystal silicon production. Carbon in fossil fuels is released as carbon dioxide during combustion of said fossil fuels. Furthermore, use of natural gas results in fugitive emissions of methane. Carbon dioxide and methane are a greenhouse gasses that increase the radiative forcing of the atmosphere, thus contributing to climate change. However, in a decarbonized economy (i.e. SSP2-RCP19), fewer fossil fuels are used in energy generation and, therefore, the GHG footprint of the consumed electricity diminishes. When fossil fuels are used, this is in combination with carbon capture and storage (see Tab. S9). This in turn makes the GHG footprint of producing a PERC panel less sensitive to uncertainty in process-specific learning curves for electricity consumption. As a result, the next two main contributors, glass and the frame (see Fig. S23) become the largest contributors to uncertainty in the GHG footprint of producing a PERC panel. The uncertainty in projected GHG footprints from PERC panel production might then be reduced by directing research efforts towards reducing uncertainty for the process-specific learning curve of the thickness of the glass and the mass of the frame, e.g. by collecting more datapoints or by further disaggregating this learning curve into multiple process-specific learning curves for producing a frame.

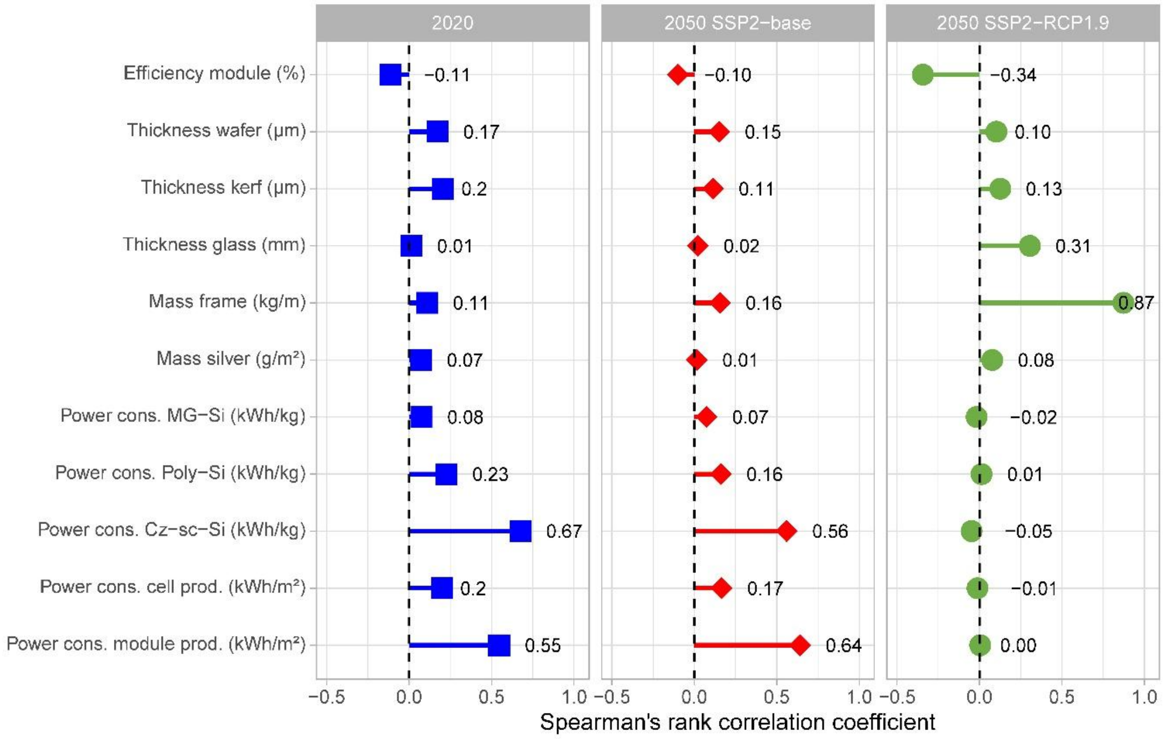

Fig. S24. *Uncertainty analyses showing the Spearman's rank correlation coefficients relating the 1,000 GHG footprints obtained for each scenario against the eleven process parameters adapted in the foreground system using learning curves. F: foreground; B: background; MG-Si: metallurgical grade silicon; poly-Si: poly-silicon; Cz-sc-Si: Czochralski single-crystalline silicon; cons.: consumption; prod.: production.*

2.3.3. Marine ecotoxicity

The output of the *print_recursive_calculation* is as follows:

```
Fraction of score | Absolute score | Amount | Activity
0001 | 237.7 |      1 | 'glass-backsheet PERC module production' (watt peak, CN, None)
*0001 | 237.7 | 0.005052 | 'glass-backsheet PERC module production' (square meter, CN, None)
**0.501 | 119.2 | 0.004537 | 'PERC cell production, mono Si M6 wafer' (square meter, CN, None)
***0.125 | 29.75 | 0.004627 | '170 µm mono M6 bricking and wafer production, photovoltaic' (square m
****0.098 |  23.3 | 0.003276 | 'p-type silicon production, single crystal, Czochralski process' (kilo
*****0.0601 | 14.27 | 0.002093 | 'silicon production, solar grade, modified Siemens process' (kilogram,
******0.0445 | 10.58 | 0.1507 | 'market group for electricity, medium voltage' (kilowatt hour, CN, Non
*******0.0394 | 9.361 | 0.1247 | 'market group for electricity, medium voltage' (kilowatt hour, CN-SGCC
*****0.0371 | 8.829 | 0.1258 | 'market group for electricity, medium voltage' (kilowatt hour, CN, Non
******0.0329 | 7.813 | 0.104 | 'market group for electricity, medium voltage' (kilowatt hour, CN-SGCC
***0.0517 | 12.28 | 4.627e-06 | 'metallization paste production, back side' (kilogram, CN, None)
****0.0517 | 12.28 | 2.314e-06 | 'market for silver' (kilogram, GLO, None)
*****0.0461 | 10.96 | 1.74e-06 | 'silver-gold mine operation with refinery' (kilogram, RoW, None)
***0.296 | 70.27 | 1.579e-05 | 'market for metallization paste, front side' (kilogram, RER, None)
****0.296 | 70.27 | 1.579e-05 | 'metallization paste production, front side' (kilogram, RER, None)
*****0.296 | 70.26 | 1.323e-05 | 'market for silver' (kilogram, GLO, None)
******0.264 | 62.68 | 9.953e-06 | 'silver-gold mine operation with refinery' (kilogram, RoW, None)
*******0.0399 | 9.483 | -0.01394 | 'market for sulfidic tailings, from silver mine operation' (kilogram,
*******0.129 | 30.59 | -0.04498 | 'market for sulfidic tailings, from silver mine operation' (kilogram,
**0.0643 | 15.27 | 0.007125 | 'aluminium alloy production, AlMg3' (kilogram, CN, None)
***0.0651 | 15.49 | 0.007125 | 'aluminium alloy production, AlMg3' (kilogram, RER, None)
****0.0518 | 12.32 | 0.006875 | 'market for aluminium, cast alloy' (kilogram, GLO, None)
**0.333 |  79.1 | 0.0007477 | 'market for copper, cathode' (kilogram, GLO, None)
***0.146 | 34.72 | 0.0001275 | 'copper production, cathode, solvent extraction and electrowinning pro
****0.0377 |  8.97 | -0.0181 | 'market for sulfidic tailings, from copper mine operation' (kilogram,
*****-0.0377 | -8.97 | 0.0181 | 'treatment of sulfidic tailings, from copper mine operation, tailings
****0.0293 | 6.956 | -0.01395 | 'market for sulfidic tailings, from copper mine operation' (kilogram,
*****-0.0293 | -6.956 | 0.01395 | 'treatment of sulfidic tailings, from copper mine operation, tailings
***0.179 | 42.55 | 0.0004523 | 'electrorefining of copper, anode' (kilogram, GLO, None)
****0.178 | 42.27 | 0.000449 | 'market for copper, anode' (kilogram, GLO, None)
*****0.0636 | 15.12 | 0.0001675 | 'smelting of copper concentrate, sulfide ore' (kilogram, RoW, None)
******0.0557 | 13.25 | 0.0005578 | 'market for copper concentrate, sulfide ore' (kilogram, GLO, None)
*****0.0752 | 17.86 | 0.0001708 | 'smelting of copper concentrate, sulfide ore' (kilogram, CN, None)
******0.0661 | 15.72 | 0.0006618 | 'market for copper concentrate, sulfide ore' (kilogram, GLO, None)
```

The Sankey diagram for this output is provided in Fig. S25.

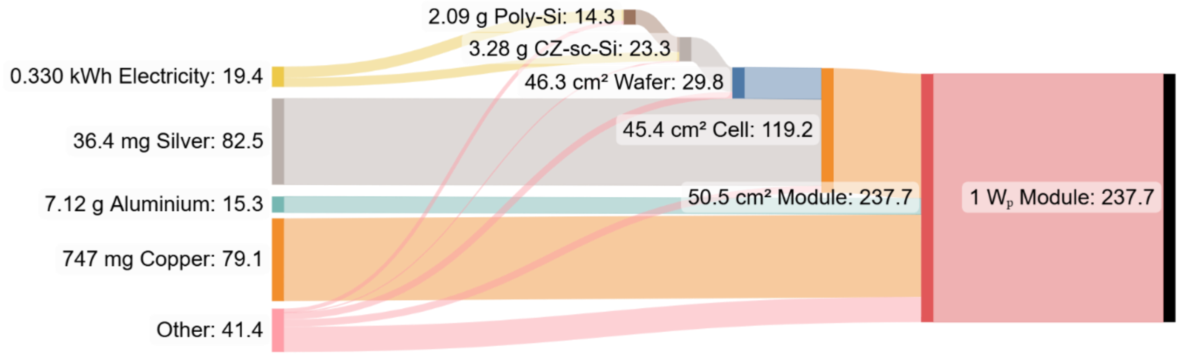

Fig. S25. *Sankey diagram of the supply chain for the production of 1 Watt-peak (W_p) of PERC solar panel capacity. Values behind each colon represent the non-carcinogenic human toxicity footprint of that product in kg 1,4-DCB-Eq./W_p. poly-Si: poly-silicon; Cz-sc-Si: Czochralski single-crystalline silicon.*

Results for the OAT sensitivity analyses of the ReCiPe 2016 (E) marine ecotoxicity midpoint impact category are provided in Tab. S18. The values in rows labelled F, B, and F+B correspond with impact reductions due to developments in the foreground, background, or both, respectively. The percentage change in marine ecotoxicity footprint is calculated between 2020 and 2050 ($\Delta_{2020\rightarrow2050}$). The final eleven rows represent OAT sensitivity values when excluding a single learning curve in the foreground system, where the percentage change in marine ecotoxicity footprint is calculated between "F+B" and "F+B, excl. [a single learning curve (e.g. efficiency)]". From Tab. S18 it becomes apparent that exclusion of learning in silver consumption results in the largest increase in the marine ecotoxicity footprint and, therefore, the marine ecotoxicity footprint is most sensitive to this process parameter.

Tab. S18. *One-at-a-time sensitivity analyses for the ReCiPe 2016 (E) marine ecotoxicity midpoint impact category showing how sensitive the percentage impact reductions between 2020 and 2050 are to modelled developments in only the foreground system (F), only the background system (B), or both (F+B), and to exclusion of individual learning curves for the foreground system.*

	**2020**			
**Baseline**	**kg 1,4-DCB-eq.**			
	229.1921			
	**2050 SSP2-base**		**2050 SSP2-RCP1.9**	
	**kg 1,4-DCB-eq.**	**$\Delta_{2020\rightarrow2050}$**	**kg 1,4-DCB-eq.**	**$\Delta_{2020\rightarrow2050}$**
**F**	173.6386	−24%	161.1958	−30%
**B**	229.7428	0%	208.6201	−9%
**F+B**	174.2763	−24%	148.0903	−35%
	**kg 1,4-DCB-eq.**	**$\Delta_{FB\rightarrow FB\ excl.}$**	**kg 1,4-DCB-eq.**	**$\Delta_{2020\rightarrow2050}$**
**F+B, excluding efficiency**	187.3972	7.5%	162.8432	10.0%
**F+B, excluding thickness wafer**	175.7739	0.9%	148.8852	0.5%
**F+B, excluding thickness kerf**	175.839	0.9%	148.8956	0.5%
**F+B, excluding thickness glass**	174.6353	0.2%	148.4902	0.3%
**F+B, excluding mass frame**	174.7767	0.3%	148.695	0.4%
**F+B, excluding mass silver**	205.5963	18.0%	185.2101	25.1%
**F+B, excluding power consumption MG-Si**	174.3901	0.1%	148.1407	0.0%
**F+B, excluding power consumption poly-Si**	174.9935	0.4%	148.408	0.2%
**F+B, excluding power consumption Cz-sc-Si**	175.4208	0.7%	148.5772	0.3%
**F+B, excluding power consumption cell production**	175.7197	0.8%	148.7452	0.4%
**F+B, excluding power consumption module production**	174.4976	0.1%	148.1937	0.1%

Fig. S26 displays the Spearman's rank correlation coefficients between the marine ecotoxicity footprint of PERC panel production and each of the eleven parameters for which process-specific learning curves were created. The higher the Spearman's rank correlation coefficient of a process parameter, the more it contributes to the uncertainty in the marine ecotoxicity footprint of PERC panel production. Module efficiency has a negative correlation coefficient, meaning that higher module efficiency correlate with lower marine ecotoxicity footprints. The other process parameters all have positive correlation coefficients, meaning that an increase in these parameter values result in a higher marine ecotoxicity footprints.

In the SSP2-base scenarios, the uncertainty in the marine ecotoxicity footprint is most affected by uncertainty in the main contributors (see Fig. S27), which is silver consumption in wafer production. Release of heavy metals during silver mining contribute to non-carcinogenic human toxicity. The uncertainty in the non-carcinogenic human toxicity footprint might be reduced by directing research efforts towards reducing uncertainty for the process-specific learning curve of the mass of silver, e.g. by collecting more datapoints or by further disaggregating the learning curve of the mass of silver into multiple process-specific learning curves for producing metallization paste or a frame.

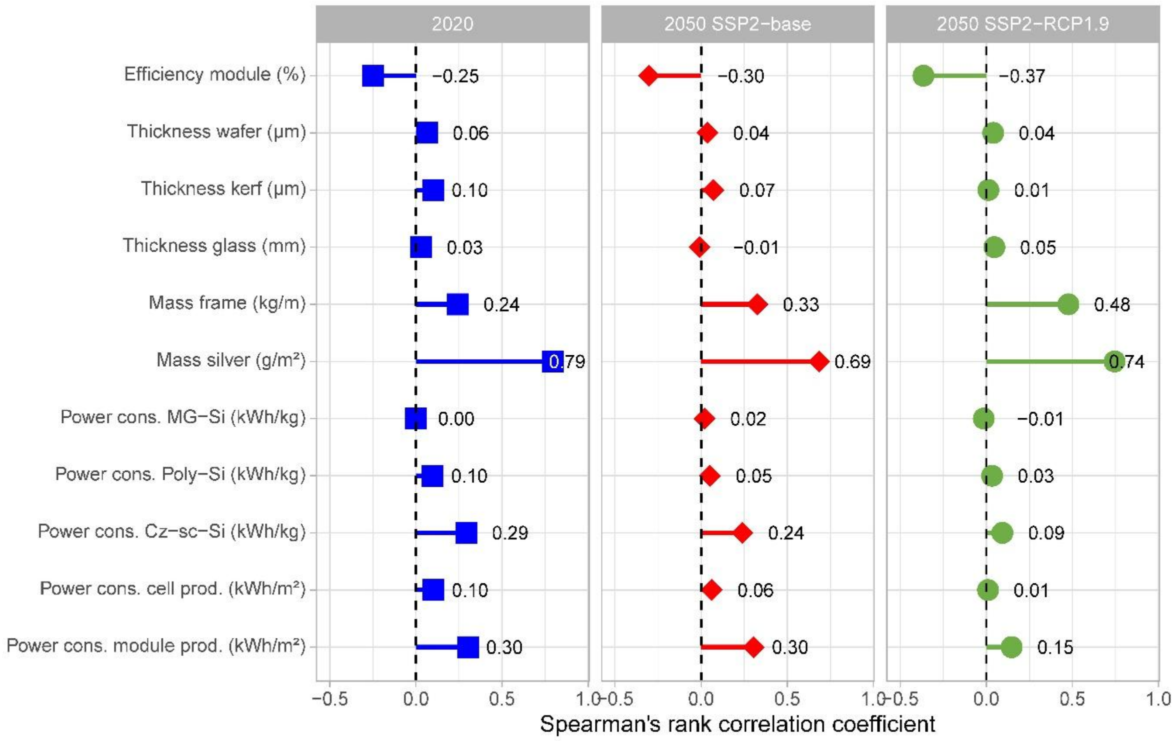

Fig. S26. *Uncertainty analyses showing the Spearman's rank correlation coefficients relating the 1,000 marine ecotoxicity footprints obtained for each scenario against the eleven process parameters adapted in the foreground system using learning curves. F: foreground; B: background; MG-Si: metallurgical grade silicon; poly-Si: poly-silicon; Cz-sc-Si: Czochralski single-crystalline silicon; cons.: consumption; prod.: production.*

2.3.4. Human Toxicity: Non-Carcinogenic

The output of the *print_recursive_calculation* is as follows:

```
Fraction of score | Absolute score | Amount | Activity
0001 | 291.2 |     1 | 'glass-backsheet PERC module production' (watt peak, CN, None)
*0001 | 291.2 | 0.005052 | 'glass-backsheet PERC module production' (square meter, CN, None)
**0.489 | 142.4 | 0.004537 | 'PERC cell production, mono Si M6 wafer' (square meter, CN, None)
***0.119 | 34.69 | 0.004627 | '170 µm mono M6 bricking and wafer production, photovoltaic' (square m
****0.0926 | 26.97 | 0.003276 | 'p-type silicon production, single crystal, Czochralski process' (kilo
*****0.0568 | 16.54 | 0.002093 | 'silicon production, solar grade, modified Siemens process' (kilogram,
******0.0419 | 12.21 | 0.1507 | 'market group for electricity, medium voltage' (kilowatt hour, CN, Non
*******0.0371 |  10.8 | 0.1247 | 'market group for electricity, medium voltage' (kilowatt hour, CN-SGCC
*****0.035 | 10.19 | 0.1258 | 'market group for electricity, medium voltage' (kilowatt hour, CN, Non
******0.0309 | 9.012 | 0.104 | 'market group for electricity, medium voltage' (kilowatt hour, CN-SGCC
***0.0508 | 14.79 | 4.627e-06 | 'metallization paste production, back side' (kilogram, CN, None)
****0.0508 | 14.79 | 2.314e-06 | 'market for silver' (kilogram, GLO, None)
*****0.0456 | 13.27 | 1.74e-06 | 'silver-gold mine operation with refinery' (kilogram, RoW, None)
***0.291 | 84.61 | 1.579e-05 | 'market for metallization paste, front side' (kilogram, RER, None)
****0.291 | 84.61 | 1.579e-05 | 'metallization paste production, front side' (kilogram, RER, None)
*****0.291 |  84.6 | 1.323e-05 | 'market for silver' (kilogram, GLO, None)
******0.261 | 75.91 | 9.953e-06 | 'silver-gold mine operation with refinery' (kilogram, RoW, None)
*******0.0395 | 11.51 | -0.01394 | 'market for sulfidic tailings, from silver mine operation' (kilogram,
*******0.128 | 37.13 | -0.04498 | 'market for sulfidic tailings, from silver mine operation' (kilogram,
**0.0915 | 26.65 | 0.007125 | 'aluminium alloy production, AlMg3' (kilogram, CN, None)
***0.0923 | 26.89 | 0.007125 | 'aluminium alloy production, AlMg3' (kilogram, RER, None)
****0.0787 | 22.92 | 0.006875 | 'market for aluminium, cast alloy' (kilogram, GLO, None)
*****0.0399 | 11.62 | 0.001693 | 'treatment of aluminium scrap, post-consumer, prepared for recycling,
******0.0304 | 8.839 | 0.001744 | 'market for aluminium scrap, post-consumer, prepared for melting' (kil
**0.323 | 94.18 | 0.0007477 | 'market for copper, cathode' (kilogram, GLO, None)
***0.15 | 43.66 | 0.0001275 | 'copper production, cathode, solvent extraction and electrowinning pro
****0.0388 |  11.3 | -0.0181 | 'market for sulfidic tailings, from copper mine operation' (kilogram,
*****-0.0388 | -11.3 | 0.0181 | 'treatment of sulfidic tailings, from copper mine operation, tailings
****0.03 | 8.748 | -0.01395 | 'market for sulfidic tailings, from copper mine operation' (kilogram,
*****-0.03 | -8.748 | 0.01395 | 'treatment of sulfidic tailings, from copper mine operation, tailings
***0.166 | 48.46 | 0.0004523 | 'electrorefining of copper, anode' (kilogram, GLO, None)
****0.165 | 48.14 | 0.000449 | 'market for copper, anode' (kilogram, GLO, None)
*****0.0589 | 17.15 | 0.0001675 | 'smelting of copper concentrate, sulfide ore' (kilogram, RoW, None)
******0.057 | 16.59 | 0.0005578 | 'market for copper concentrate, sulfide ore' (kilogram, GLO, None)
*****0.0697 | 20.28 | 0.0001708 | 'smelting of copper concentrate, sulfide ore' (kilogram, CN, None)
******0.0676 | 19.68 | 0.0006618 | 'market for copper concentrate, sulfide ore' (kilogram, GLO, None)
```

The Sankey diagram for this output is provided in Fig. S27.

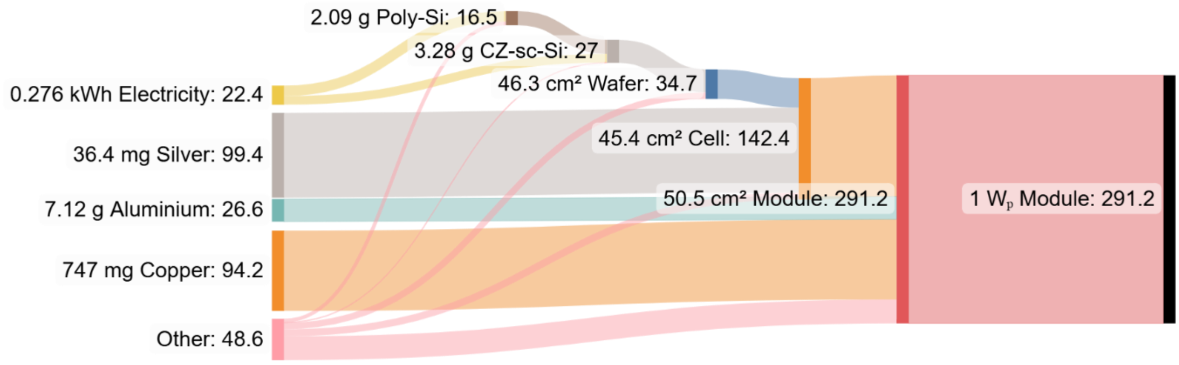

Fig. S27. *Sankey diagram of the supply chain for the production of 1 Watt-peak (W_p) of PERC solar panel capacity. Values behind each colon represent the marine ecotoxicity footprint of that product in kg 1,4-DCB-Eq./W_p. poly-Si: poly-silicon; Cz-sc-Si: Czochralski single-crystalline silicon.*

Results for the OAT sensitivity analyses of the ReCiPe 2016 (E) non-carcinogenic human toxicity midpoint impact category are provided in Tab. S19. The values in rows labelled F, B, and F+B correspond with impact reductions due to developments in the foreground, background, or both, respectively. The percentage change in non-carcinogenic human toxicity footprint is calculated between 2020 and 2050 ($\Delta_{2020 \rightarrow 2050}$). The final eleven rows represent OAT sensitivity values when excluding a single learning curve in the foreground system, where the percentage change in non-carcinogenic human toxicity footprint is calculated between "F+B" and "F+B, excl. [a single learning curve (e.g. efficiency)]". From Tab. S19 it becomes apparent that exclusion of learning in silver consumption results in the largest increase in the non-carcinogenic human toxicity footprint and, therefore, the non-carcinogenic human toxicity footprint is most sensitive to this process parameter.

Tab. S19. *One-at-a-time sensitivity analyses for the ReCiPe 2016 (E) human non-carcinogenic toxicity midpoint impact category showing how sensitive the percentage impact reductions between 2020 and 2050 are to modelled developments in only the foreground system (F), only the background system (B), or both (F+B), and to exclusion of individual learning curves for the foreground system.*

**Baseline**	**2020**
	**kg 1,4-DCB-eq.**
	190.0039

	**2050 SSP2-base**		**2050 SSP2-RCP1.9**	
	**kg 1,4-DCB-eq.**	**$\Delta_{2020 \rightarrow 2050}$**	**kg 1,4-DCB-eq.**	**$\Delta_{2020 \rightarrow 2050}$**
**F**	143.8001	−24%	133.4637	−30%
**B**	190.275	0%	170.802	−10%
**F+B**	144.2331	−24%	121.292	−36%
	**kg 1,4-DCB-eq.**	**$\Delta_{FB \rightarrow FB\ excl.}$**	**kg 1,4-DCB-eq.**	**$\Delta_{2020 \rightarrow 2050}$**
**F+B, excluding efficiency**	155.0922	7.5%	133.3752	10.0%
**F+B, excluding thickness wafer**	145.5059	0.9%	121.8872	0.5%
**F+B, excluding thickness kerf**	145.5612	0.9%	121.8949	0.5%
**F+B, excluding thickness glass**	144.5313	0.2%	121.62	0.3%
**F+B, excluding mass frame**	144.5172	0.2%	121.6251	0.3%
**F+B, excluding mass silver**	170.2408	18.0%	152.1002	25.4%
**F+B, excluding power consumption MG-Si**	144.3301	0.1%	121.3282	0.0%
**F+B, excluding power consumption poly-Si**	144.8444	0.4%	121.5202	0.2%
**F+B, excluding power consumption Cz-sc-Si**	145.2085	0.7%	121.6417	0.3%
**F+B, excluding power consumption cell production**	145.4632	0.9%	121.7624	0.4%
**F+B, excluding power consumption module production**	144.4218	0.1%	121.3663	0.1%

Fig. S28 displays the Spearman's rank correlation coefficients between the non-carcinogenic human toxicity footprint of PERC panel production and each of the eleven parameters for which process-specific learning curves were created. The higher the Spearman's rank correlation coefficient of a process parameter, the more it contributes to the uncertainty in the non-carcinogenic human toxicity footprint of PERC panel production. Module efficiency has a negative correlation coefficient, meaning that higher module efficiency correlate with lower non-carcinogenic human toxicity footprints. The other process parameters all have positive correlation coefficients, meaning that an increase in these parameter values result in a higher non-carcinogenic human toxicity footprints.

In the SSP2-base scenarios, the uncertainty in the non-carcinogenic human toxicity footprint is most affected by uncertainty in the main contributors (see Fig. S27), which is silver consumption in wafer production. Release of heavy metals during silver mining contribute to non-carcinogenic human toxicity. The uncertainty in the non-carcinogenic human toxicity footprint might be reduced by directing research efforts towards reducing uncertainty for the process-specific learning curve of the mass of silver, e.g. by collecting more datapoints or by further disaggregating the learning curve of the mass of silver into multiple process-specific learning curves for producing metallization paste or a frame.

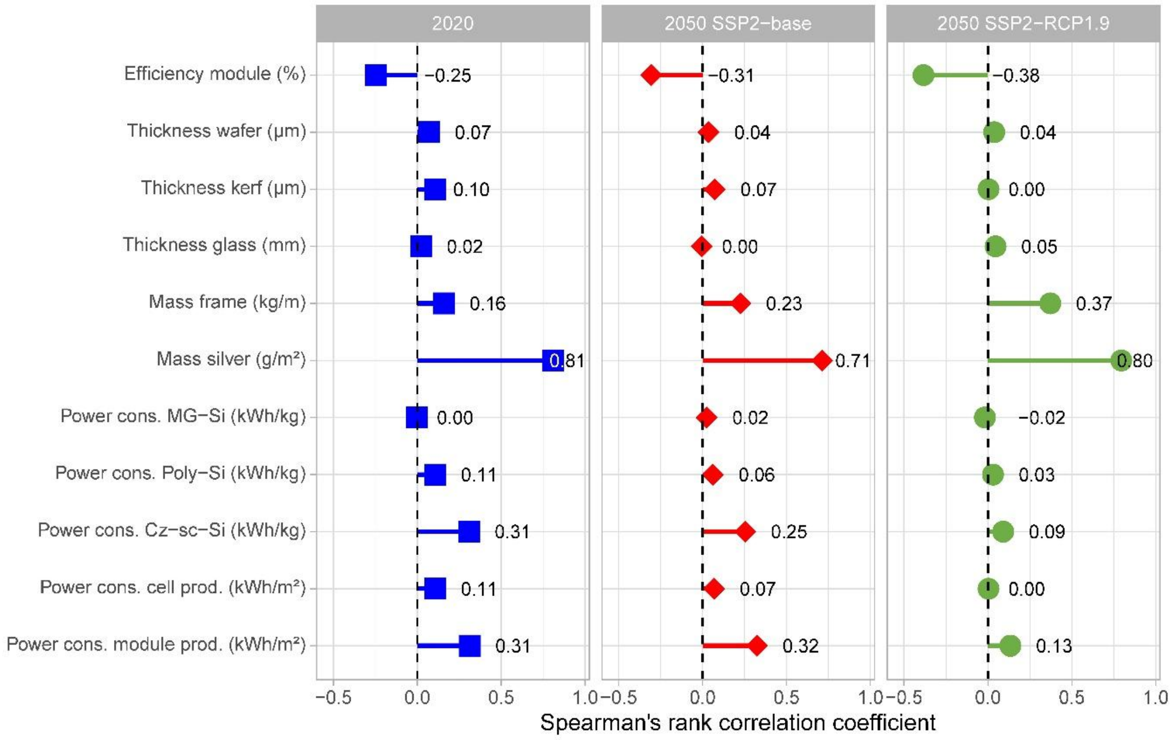

Fig. S28. *Uncertainty analyses showing the Spearman's rank correlation coefficients relating the 1,000 non-carcinogenic human toxicity footprints obtained for each scenario against the eleven process parameters adapted in the foreground system using learning curves. F: foreground; B: background; MG-Si: metallurgical grade silicon; poly-Si: poly-silicon; Cz-sc-Si: Czochralski single-crystalline silicon; cons.: consumption; prod.: production.*

2.3.1. Energy Resources: Non-renewable, Fossil

The output of the *print_recursive_calculation* is as follows:

```
Fraction of score | Absolute score | Amount | Activity
0001 | 0.1345 |     1 | 'glass-backsheet PERC module production' (watt peak, CN, None)
*0001 | 0.1345 | 0.005052 | 'glass-backsheet PERC module production' (square meter, CN, None)
**0.63 | 0.08474 | 0.004537 | 'PERC cell production, mono Si M6 wafer' (square meter, CN, None)
***0.53 | 0.0713 | 0.004627 | '170 µm mono M6 bricking and wafer production, photovoltaic' (square m
****00.5 | 0.06718 | 0.003276 | 'p-type silicon production, single crystal, Czochralski process' (kilo
*****0.313 | 0.0421 | 0.002093 | 'silicon production, solar grade, modified Siemens process' (kilogram,
******0.0559 | 0.007515 | 0.002365 | 'silicon production, metallurgical grade' (kilogram, CN, None)
*******0.0383 | 0.005149 | 0.02602 | 'market group for electricity, medium voltage' (kilowatt hour, CN, Non
******0.0283 | 0.003809 | 0.1465 | 'heat production, natural gas, at industrial furnace >100kW' (megajoul
******0.222 | 0.02982 | 0.1507 | 'market group for electricity, medium voltage' (kilowatt hour, CN, Non
*******0.196 | 0.02629 | 0.1247 | 'market group for electricity, medium voltage' (kilowatt hour, CN-SGCC
*******0.0263 | 0.003536 | 0.02605 | 'market for electricity, medium voltage' (kilowatt hour, CN-CSG, None)
*****0.185 | 0.02489 | 0.1258 | 'market group for electricity, medium voltage' (kilowatt hour, CN, Non
******0.163 | 0.02194 | 0.104 | 'market group for electricity, medium voltage' (kilowatt hour, CN-SGCC
*******0.044 | 0.005921 | 0.02382 | 'market for electricity, medium voltage' (kilowatt hour, CN-NCGC, None
*******0.0384 | 0.005167 | 0.02828 | 'market for electricity, medium voltage' (kilowatt hour, CN-ECGC, None
*******0.0337 | 0.00453 | 0.01542 | 'market for electricity, medium voltage' (kilowatt hour, CN-NECG, None
***0.0403 | 0.005414 | 0.02736 | 'market group for electricity, medium voltage' (kilowatt hour, CN, Non
****0.0355 | 0.004772 | 0.02263 | 'market group for electricity, medium voltage' (kilowatt hour, CN-SGCC
**0.0947 | 0.01274 | 0.007125 | 'aluminium alloy production, AlMg3' (kilogram, CN, None)
***0.0862 | 0.01159 | 0.007125 | 'aluminium alloy production, AlMg3' (kilogram, RER, None)
****0.0633 | 0.008507 | 0.006875 | 'market for aluminium, cast alloy' (kilogram, GLO, None)
*****0.0558 | 0.007504 | 0.001797 | 'aluminium ingot, primary, to aluminium, cast alloy market' (kilogram,
******0.0487 | 0.006546 | 0.001396 | 'market for aluminium, primary, ingot' (kilogram, RoW, None)
*******0.034 | 0.004568 | 0.0009819 | 'aluminium production, primary, ingot' (kilogram, CN, None)
**0.0753 | 0.01013 | 0.04042 | 'flat glass production, uncoated' (kilogram, CN, None)
***0.072 | 0.009679 | 0.04042 | 'flat glass production, uncoated' (kilogram, RER, None)
****0.0337 | 0.004531 | 0.004692 | 'market group for natural gas, high pressure' (cubic meter, Europe wit
**0.0549 | 0.007385 | 0.004006 | 'market for ethylvinylacetate, foil' (kilogram, GLO, None)
***0.0371 | 0.004982 | 0.002682 | 'ethylvinylacetate production, foil' (kilogram, RoW, None)
****0.0342 | 0.004599 | 0.002735 | 'market for ethylene vinyl acetate copolymer' (kilogram, RoW, None)
*****0.0336 | 0.004524 | 0.002735 | 'ethylene vinyl acetate copolymer production' (kilogram, RoW, None)
```

The Sankey diagram for this output is provided Fig. S29.

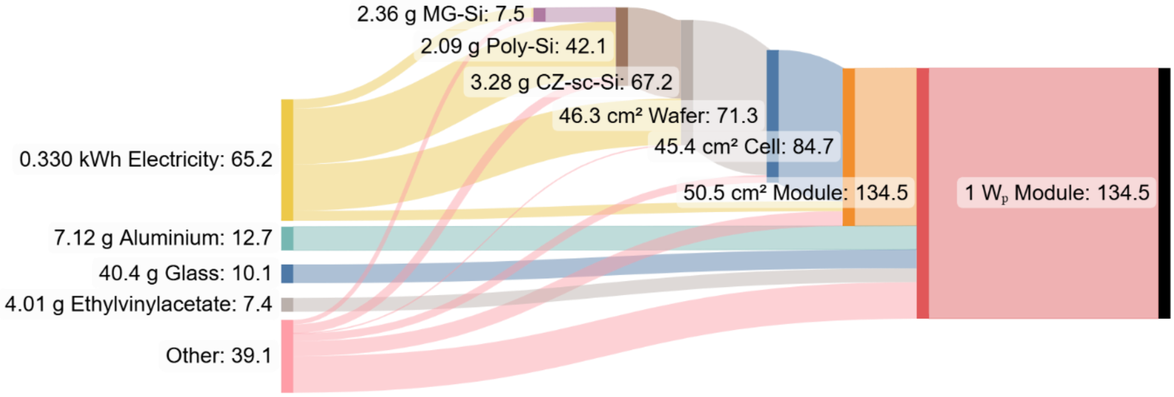

Fig. S29. *Sankey diagram of the supply chain for the production of 1 Watt-peak (W_p) of PERC solar panel capacity. Values behind each colon represent the non-renewable fossil energy resource footprint of that product in g oil-Eq./W_p. MG-Si: metallurgical grade silicon; poly-Si: poly-silicon; Cz-sc-Si: Czochralski single-crystalline silicon.*

Results for the OAT sensitivity analyses of the ReCiPe 2016 (E) non-renewable fossil energy resources midpoint impact category are provided in Tab. S20. The values in rows labelled F, B, and F+B correspond with impact reductions due to developments in the foreground, background, or both, respectively. The percentage change in non-renewable fossil energy resources footprint is calculated between 2020 and 2050 ($\Delta_{2020\rightarrow2050}$). The final eleven rows represent OAT sensitivity values when excluding a single learning curve in the foreground system, where the percentage change in non-renewable fossil energy resources footprint is calculated between "F+B" and "F+B, excl. [a single learning curve (e.g. efficiency)]". From Tab. S20 it becomes apparent that exclusion of learning in panel efficiency results in the largest increase in non-renewable fossil energy resources footprint and, therefore, the non-renewable fossil energy resources footprint is most sensitive to this process parameter.

Tab. S20. *One-at-a-time sensitivity analyses for the ReCiPe 2016 (E) non-renewable fossil energy resources midpoint impact category showing how sensitive the percentage impact reductions between 2020 and 2050 are to modelled developments in only the foreground system (F), only the background system (B), or both (F+B), and to exclusion of individual learning curves for the foreground system.*

	**2020**
**Baseline**	**kg oil-eq.**
	0.09639567

	**2050 SSP2-base**		**2050 SSP2-RCP1.9**	
	**kg oil-eq.**	**$\Delta_{2020\rightarrow2050}$**	**kg oil-eq.**	**$\Delta_{2020\rightarrow2050}$**
**F**	0.071628	−26%	0.065913	−32%
**B**	0.088693	−8%	0.048759	−49%
**F+B**	0.066482	−31%	0.037014	−62%
	**kg oil-eq.**	**$\Delta_{FB\rightarrow FB\ excl.}$**	**kg oil-eq.**	**$\Delta_{2020\rightarrow2050}$**
**F+B, excluding efficiency**	0.071488	7.5%	0.040701	10.0%
**F+B, excluding thickness wafer**	0.069607	4.7%	0.038585	4.2%
**F+B, excluding thickness kerf**	0.069743	4.9%	0.038605	4.3%
**F+B, excluding thickness glass**	0.067245	1.1%	0.037900	2.4%
**F+B, excluding mass frame**	0.066688	0.3%	0.037225	0.6%
**F+B, excluding mass silver**	0.066991	0.8%	0.037333	0.9%
**F+B, excluding power consumption MG-Si**	0.066706	0.3%	0.037086	0.2%
**F+B, excluding power consumption poly-Si**	0.067892	2.1%	0.037472	1.2%
**F+B, excluding power consumption Cz-sc-Si**	0.068731	3.4%	0.037716	1.9%
**F+B, excluding power consumption cell production**	0.069319	4.3%	0.037958	2.6%
**F+B, excluding power consumption module production**	0.066917	0.7%	0.037163	0.4%

Fig. S30 displays the Spearman's rank correlation coefficients between the non-renewable fossil energy resources footprint for PERC panel production and each of the eleven parameters for which process-specific learning curves were created. The higher the Spearman's rank correlation coefficient of a process parameter, the more it contributes to the uncertainty in the non-renewable fossil energy resources footprint of PERC panel production. Module efficiency has a negative correlation coefficient, meaning that higher module efficiency correlate with lower non-renewable fossil energy resources footprints. The other process parameters all have positive correlation coefficients, meaning that an increase in these parameter values result in a higher non-renewable fossil energy resources footprints.

In the SSP2-base scenarios, the uncertainty in the non-renewable fossil energy resources footprint for PERC panel production is most affected by uncertainty in power consumption in module and Czochralski single-crystal silicon production. In a decarbonized economy (i.e. SSP2-RCP19), one would expect the non-renewable fossil energy resources footprint of the consumed electricity to diminish as more electricity is generated with renewable resources. As the consumption of fossil resources in electricity generation decreases, the non-renewable fossil energy resources footprint of producing a PERC panel would become less sensitive to uncertainty in process-specific learning curves for electricity consumption. A slight reduction is visible, but not as strong as e.g. in the categories of climate change and particulate matter formation. A reason for this can be found in the scenario files that are exported by premise (see the Supporting Information). Contributions of various sources of electricity are displayed in Tab. S9. In 2020, the major energy sources for electricity are pulverized coal and hydro. In the SSP2-RCP1.9 scenario for 2050, the main contributors are onshore wind, solar, hydro and natural gas with carbon capture and storage (CCS). The use of natural gas with CCS results in fewer emissions of greenhouse gasses and particulate matter, thus explaining the reduced contribution of electricity to the uncertainty in the GHG footprint and particulate matter formation footprints in the 20205 SSP2-RCP1.9 scenario. However, natural gas with CC still results in consumption of natural gas, thus contributing to the non-renewable fossil energy resources footprints. This is why electricity consumption remains a relatively large contributor to uncertainty in the non-renewable fossil energy resources footprints.

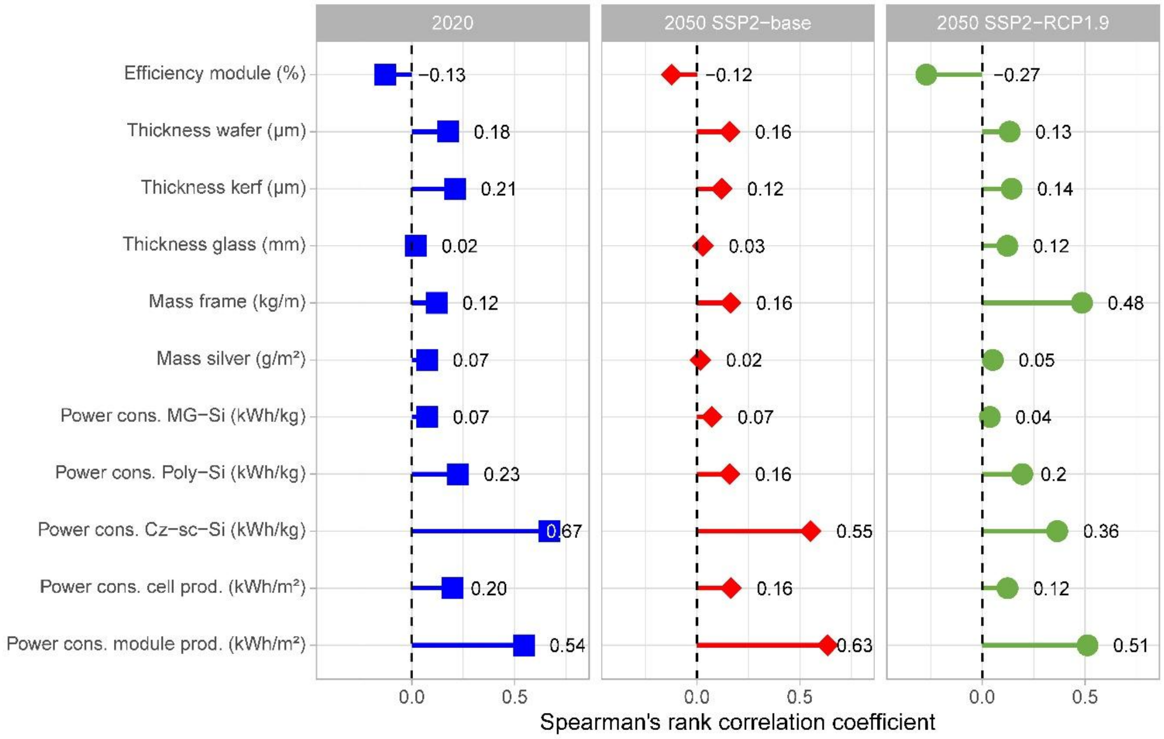

Fig. S30. *Uncertainty analyses showing the Spearman's rank correlation coefficients relating the 1,000 non-renewable fossil energy resource footprints obtained for each scenario against the eleven process parameters adapted in the foreground system using learning curves. F: foreground; B: background; MG-Si: metallurgical grade silicon; poly-Si: poly-silicon; Cz-sc-Si: Czochralski single-crystalline silicon; cons.: consumption; prod.: production.*

3. REFERENCES

[1] Müller A, Friedrich L, Reichel C, Herceg S, Mittag M, Neuhaus DH. A comparative life cycle assessment of silicon PV modules: Impact of module design, manufacturing location and inventory. Sol Energy Mater Sol Cells. 2021;230:111277.
[2] Wernet G, Bauer C, Steubing B, Reinhard J, Moreno-Ruiz E, Weidema B. The ecoinvent database version 3 (part I): overview and methodology. Int J Life Cycle Assess. 2016;21:1218–30.
[3] ecoinvent. ecoinvent database (Version 3.9.1) [Cut-off system model]. ecoinvent; 2022.
[4] Sacchi R, Terlouw T, Siala K, Dirnaichner A, Bauer C, Cox B, et al. PRospective EnvironMental Impact asSEment (premise): A streamlined approach to producing databases for prospective life cycle assessment using integrated assessment models. Renew Sustain Energy Rev. 2022;160:112311.
[5] Sacchi R, Dirnaichner A, Terlouw TM, Vandepaer L, Mutel C, Rossi M. premise. 2.0.2 ed. GitHub2024.
[6] Mutel C. Brightway: An open source framework for Life Cycle Assessment. J Open Source Softw. 2017;2:236.
[7] NREL. Solar module efficiency table (31-05-2024). 2024.
[8] Bollens U, Bosshart S, Ciot M, Ciseri L, Doka G, Frischknecht R, et al. Ökoinventare von Energiesystemen - Grundlagen für den ökologischen Vergleich von Energiesystemen und den Einbezug von Energiesystemen in Ökobilanzen für die Schweiz. 1996.
[9] Jungbluth N, Stucki M, Flury K, Frischknecht R, Büsser S. Life Cycle Inventories of Photovoltaics - Version: 2012. ESU-services Ltd.; 2012.
[10] ITRPV. International Technology Roadmap for Photovoltaics (ITRPV) Results 2012. 2013.
[11] ITRPV. International Technology Roadmap for Photovoltaics (ITRPV) - 2013 Results. 2014.
[12] ITRPV. International Technology Roadmap for Photovoltaics (ITRPV) Results 2011. 2012.
[13] ITRPV. International Technology Roadmap for Photovoltaics (ITRPV) - 2017 Results. 2018.
[14] ITRPV. International Technology Roadmap for Photovoltaics (ITRPV) - 2018 Results. 2019.
[15] ITRPV. International Technology Roadmap for Photovoltaics (ITRPV) - 2019 Results. 2020.
[16] ITRPV. International Technology Roadmap for Photovoltaics (ITRPV) - 2020 Results. Frankfurt am Main, Germany: VDMA e. V.; 2021.
[17] ITRPV. International Technology Roadmap for Photovoltaics (ITRPV) - 2021 Results. Frankfurt am Main, Germany: VDMA e. V.; 2022.
[18] ITRPV. International Technology Roadmap for Photovoltaics (ITRPV) - 2022 Results. Fourteenth Edition, April 2023 ed. Frankfurt am Main, Germany: VDMA e. V.; 2023.
[19] ITRPV. International Technology Roadmap for Photovoltaics (ITRPV) - 2023 Results. Fifteenth Edition, May 2024 ed. Frankfurt am Main, Germany: VDMA e. V.; 2024.
[20] Woodhouse M, Goodrich A, Margolis R, James TL, Lokanc M, Eggert R. Supply-Chain Dynamics of Tellurium, Indium, and Gallium Within the Context of PV Manufacturing Costs. IEEE Journal of Photovoltaics. 2013;3:833–7.
[21] ITRPV. International Technology Roadmap for Photovoltaics (ITRPV.net) Results 2010. 2011.
[22] ITRPV. International Technology Roadmap for Photovoltaics (ITRPV) - 2014 Results. 2015.
[23] ITRPV. International Technology Roadmap for Photovoltaics (ITRPV) - 2015 Results. 2016.
[24] ITRPV. International Technology Roadmap for Photovoltaics (ITRPV) - 2016 Results. 2017.
[25] Fraunhofer ISE. Photovoltaics Report - 21 February 2023. 2023.
[26] ITRPV. CTM Group* PV Roadmap for Crystalline Silicon. 2010.
[27] Jester TL. Crystalline silicon manufacturing progress. Prog Photovolt: Res Appl. 2002;10:99-106.
[28] Vedde J, Clausen T, Borregaard J, Kringhøj P. Float-Zone Crystal Growth For PV – Where Is The Future? 23rd EU PVSEC. Valencia, Spain2008. p. 3.
[29] IEA PVPS. Trends 2019 In Photovoltaic Applications. 2019.
[30] IEA PVPS. Trends 2020 In Photovoltaic Applications. 2020.
[31] IEA PVPS. Trends 2021 In Photovoltaic Applications. 2021.
[32] IEA PVPS. Trends 2022 In Photovoltaic Applications. 2022.
[33] IEA PVPS. Trends 2023 In Photovoltaic Applications. 2023.

[34] Mints P. Chapter Four - Overview of Photovoltaic Production, Markets, and Perspectives. In: Willeke GP, Weber ER, editors. Semiconductors and Semimetals: Elsevier; 2012. p. 49-84.
[35] Nemet GF. Interim monitoring of cost dynamics for publicly supported energy technologies. Energy Policy. 2009;37:825–35.
[36] Nemet GF. Beyond the learning curve: factors influencing cost reductions in photovoltaics. Energy Policy. 2006;34:3218–32.
[37] Maycock PD. PV review: World Solar PV market continues explosive growth. Refocus. 2005;6:18-22.
[38] IRENA. Statistics Time Series. In: IRENA, editor.2024.
[39] Lafond F, Gotway Bailey A, Bakker JD, Rebois D, Zadourian R, McSharry P, et al. How well do experience curves predict technological progress? A method for making distributional forecasts. 2017.
[40] bp. bp Statistical Review of World Energy 2022 | 71st edition. 2022.
[41] bp. bp Statistical Review of World Energy 2021 | 70th edition. 2021.
[42] Energy Institute. Statistical Review of World Energy 2023 | 72nd edition. 2023.
[43] EPIA. Global Market Outlook For Photovoltaics 2014-2018. 2014.
[44] EPIA. Global Market Outlook For Photovoltaics 2013-2017. 2013.
[45] EPIA. Global Market Outlook For Photovoltaics until 2015. 2011.
[46] EPIA. Global Market Outlook For Photovoltaics until 2014. 2010.
[47] SolarPower Europe. Global Market Outlook For Solar Power 2023 - 2027. 2023.
[48] EIA. International. 2024.
[49] IEA PVPS. Snapshot Reports. 2024.
[50] NREL. Cell efficiency data table (31-08-2023). 2023.
[51] NREL. Solar module efficiency table (23-05-2023). 2023.
[52] DS New Energy. Bigger Wafers, Half-Cut Technology, Multi Bus-Bar (MBB) Make Higher Power Solar Panel. 2021.
[53] DS New Energy. Solar Wafer M12(G12) M10 M9 M6 G1 M4 M2. 2020.
[54] Woodhouse MA, Smith B, Ramdas A, Margolis RM. Crystalline Silicon Photovoltaic Module Manufacturing Costs and Sustainable Pricing: 1H 2018 Benchmark and Cost Reduction Road Map. Office of Scientific and Technical Information (OSTI); 2019.
[55] Our World in Data. Solar (photovoltaic) panel prices vs. cumulative capacity. 2022.
[56] Nagy B, Swenson B, Gonzales JP, Bui QM, Lee I, Bennett N, et al. Performance Curve Database. 2024.
[57] Nagy B, Farmer JD, Bui QM, Trancik JE. Statistical Basis for Predicting Technological Progress. PLOS ONE. 2013;8:e52669.
[58] International Energy Agency. World Energy Outlook 2023. 2023.
[59] Huijbregts MAJ, Steinmann ZJN, Elshout PMF, Stam G, Verones F, Vieira M, et al. ReCiPe 2016 v1.1 A harmonized life cycle impact assessment method at midpoint and endpoint level Report I: Characterization. Bilthoven, The Netherlands: RIVM; 2017.
[60] Huijbregts MAJ, Steinmann ZJN, Elshout PMF, Stam G, Verones F, Vieira M, et al. ReCiPe2016: a harmonised life cycle impact assessment method at midpoint and endpoint level. Int J Life Cycle Assess. 2017;22:138–47.
[61] van der Hulst MK, Magoss D, Massop Y, Veenstra S, van Loon N, Dogan I, et al. Comparing Environmental Impacts of Single-Junction Silicon and Silicon/Perovskite Tandem Photovoltaics–A Prospective Life Cycle Assessment. ACS Sustain Chem Eng. 2024.
[62] Louwen A, van Sark WGJHM, Faaij APC, Schropp REI. Re-assessment of net energy production and greenhouse gas emissions avoidance after 40 years of photovoltaics development. Nat Commun. 2016;7:13728.
[63] Boyce J, Sacchi R, Goetheer E, Steubing B. A prospective life cycle assessment of global ammonia decarbonisation scenarios. Heliyon. 2024;10:e27547.
